# Supplementary figures and images for: Impact of MED12 mutation and CDK8 activity on uterine leiomyoma growth and response to gonadotropin-releasing hormone agonist treatment
Source: PLoS One. 2026 Jan 6;21(1):e0338485. doi: 10.1371/journal.pone.0338485 (PMC12773822; doi:10.1371/journal.pone.0338485)

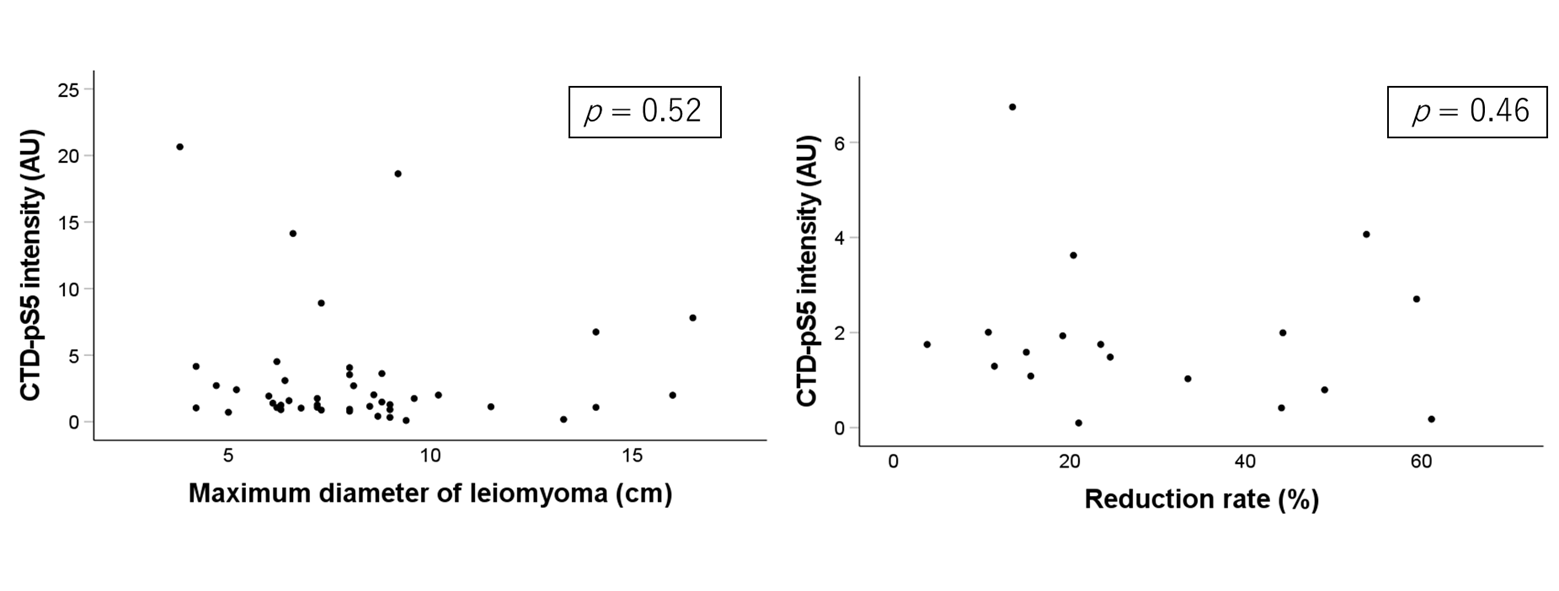

Supplement: S1 Fig — Spearman’s rank correlation coefficient was used for the analysis. (A) Correlation analysis between the amount of CTD-pS5 and maximum tumor diameter of the leiomyoma. (B) Correlation analysis between the rate of leiomyoma size reduction before and after GnRH agonist use and the amount of CTD-pS5. CTD-pS5, C-terminal domain with phosphorylated 5th serine residue; GnRH, gonadotropin-releasing hormone. (TIF) [file pone.0338485.s001.tif]

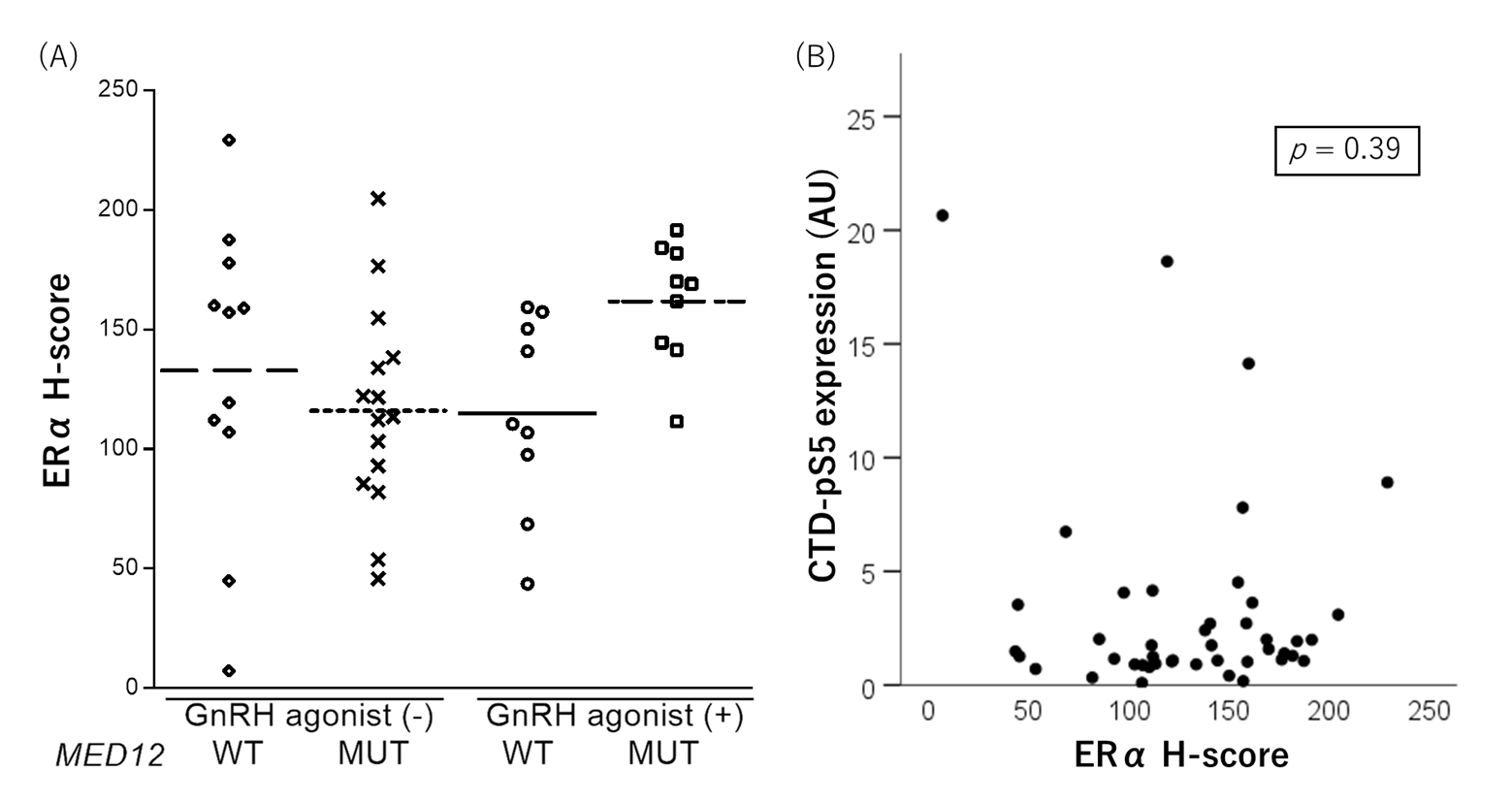

Supplement: S2 Fig — The H-score for ERα in immunostaining was calculated. (A) Comparison of ERα expression between MED12 status (WT or MUT) in GnRH agonist-treated (GnRH agonist+) and -untreated (GnRH agonist–) groups. (B) Correlation analysis between ERα expression and the amount of CTD-pS5. Spearman’s rank correlation coefficient was used for the analysis. CTD-pS5, C-terminal domain with phosphorylated 5th serine residue; ERα, estrogen receptor alpha; GnRH, gonadotropin-releasing hormone. MUT, mutant; WT, wild type. (TIF) [file pone.0338485.s002.tif]

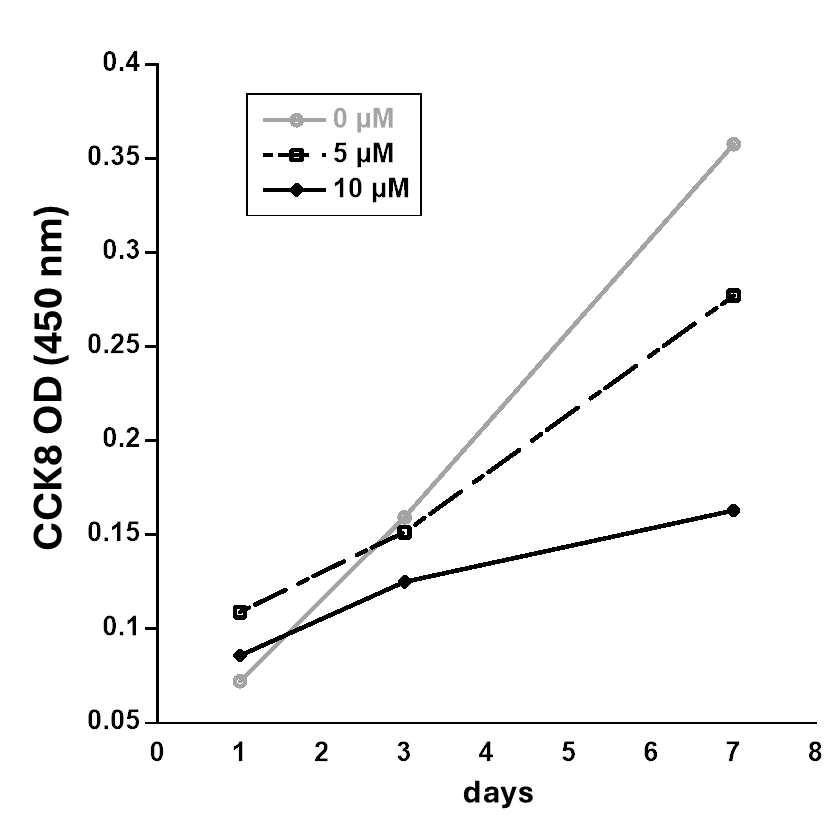

Supplement: S3 Fig — Results of CCK8 cell proliferation assay when Senexin A was added at 5 and 10 µM. (TIF) [file pone.0338485.s003.tif]

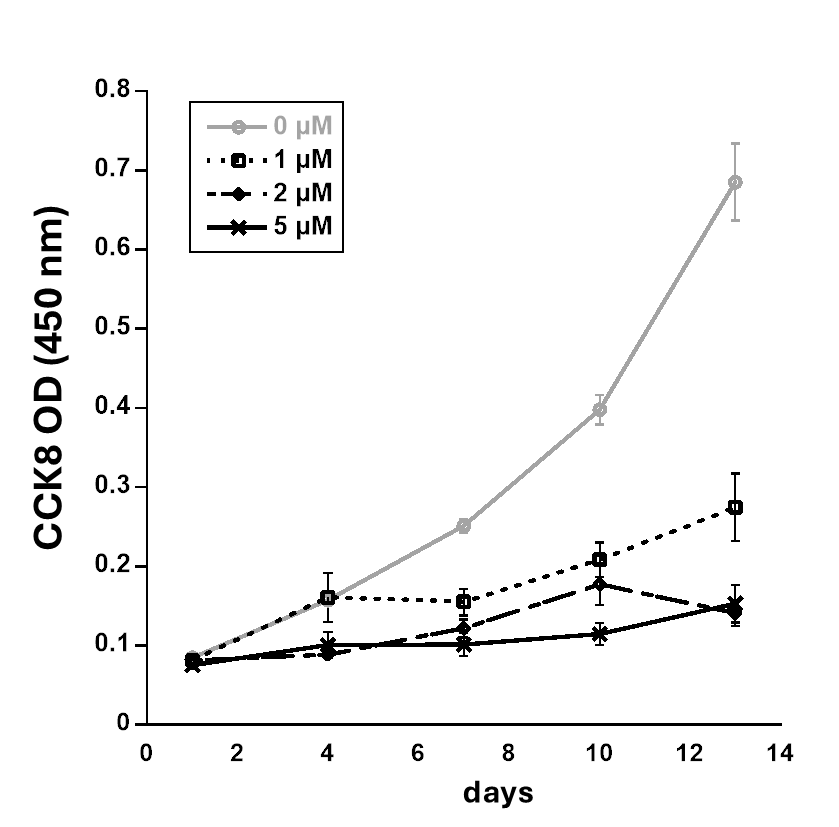

Supplement: S4 Fig — Cell proliferation was measured by the CCK8 assay by adding various concentrations of Senexin B to primary cultured leiomyoma cells. Two-way ANOVA revealed significant effects of dose, time, and their interaction (p < 0.001 for all). Post hoc analyses using Tukey’s test showed that 2 µM and 5 µM significantly inhibited cell growth compared with 1 µM (p = 0.001, p < 0.001), while no further suppression was observed between 2 and 5 µM (p = 0.76). (TIF) [file pone.0338485.s004.tif]

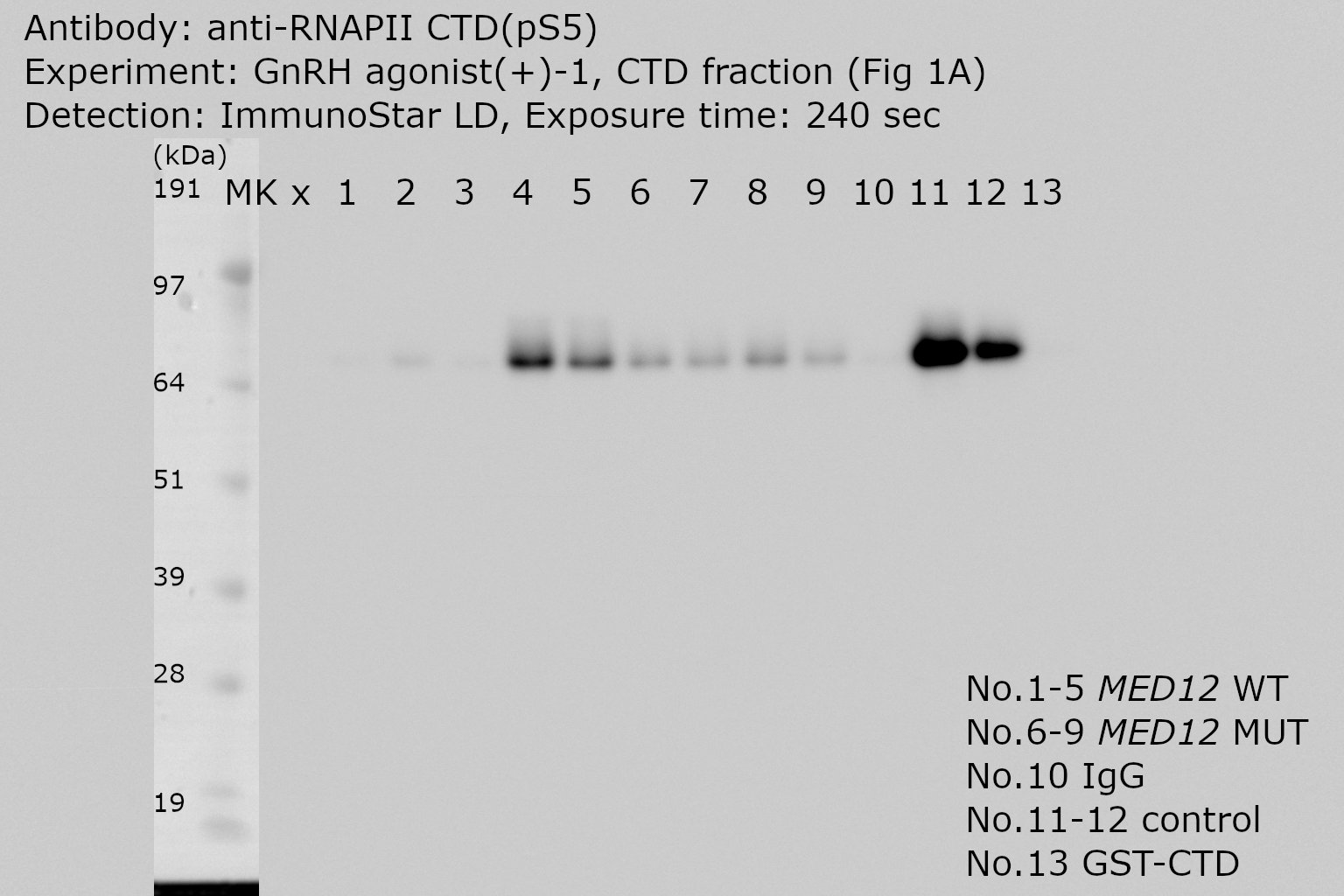

Supplement: S1 Raw Images — (ZIP) [file pone.0338485.s005.zip › CTD-pS5 GnRH+1.tif]

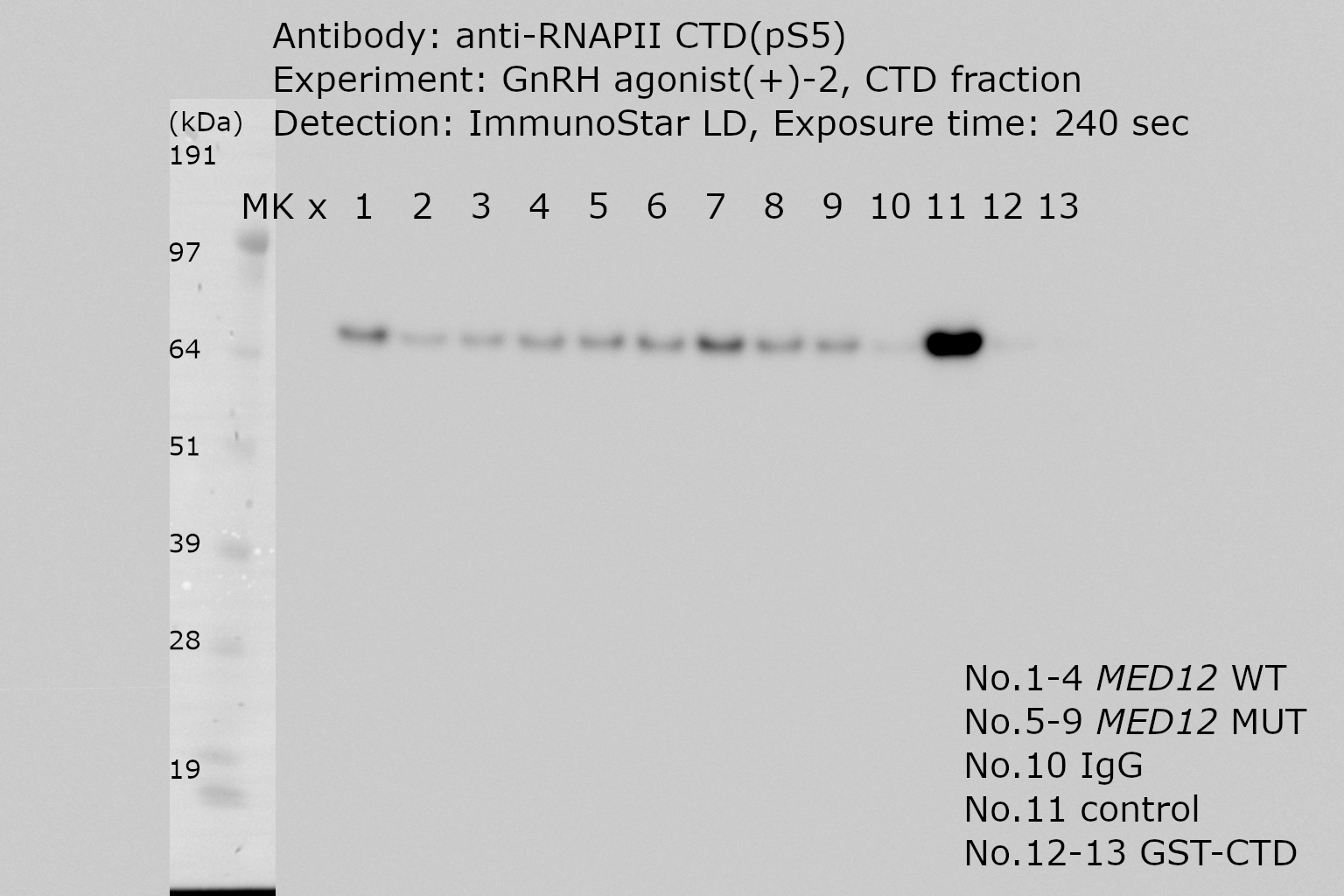

Supplement: S1 Raw Images — (ZIP) [file pone.0338485.s005.zip › CTD-pS5 GnRH+2.tif]

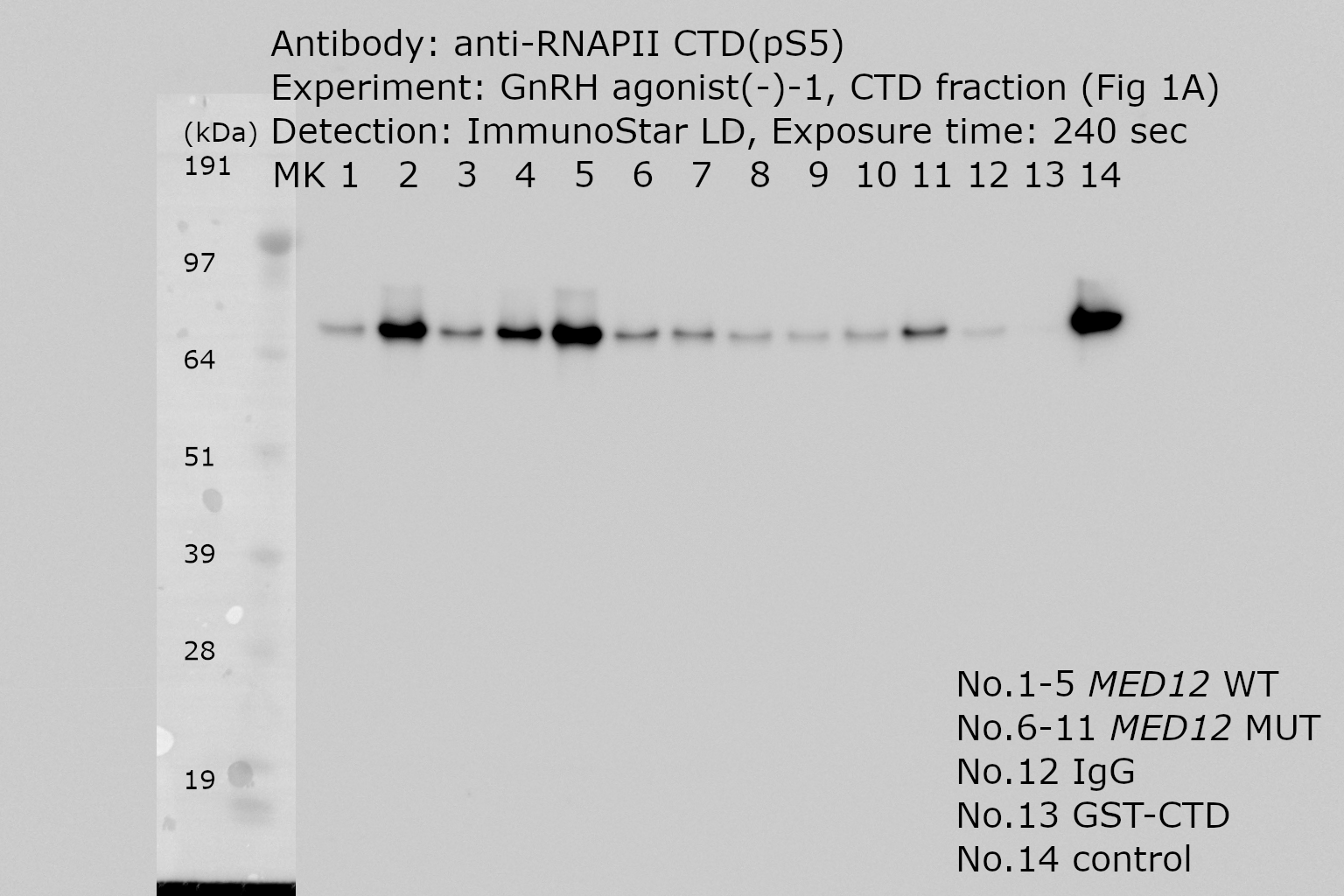

Supplement: S1 Raw Images — (ZIP) [file pone.0338485.s005.zip › CTD-pS5 GnRH-1.tif]

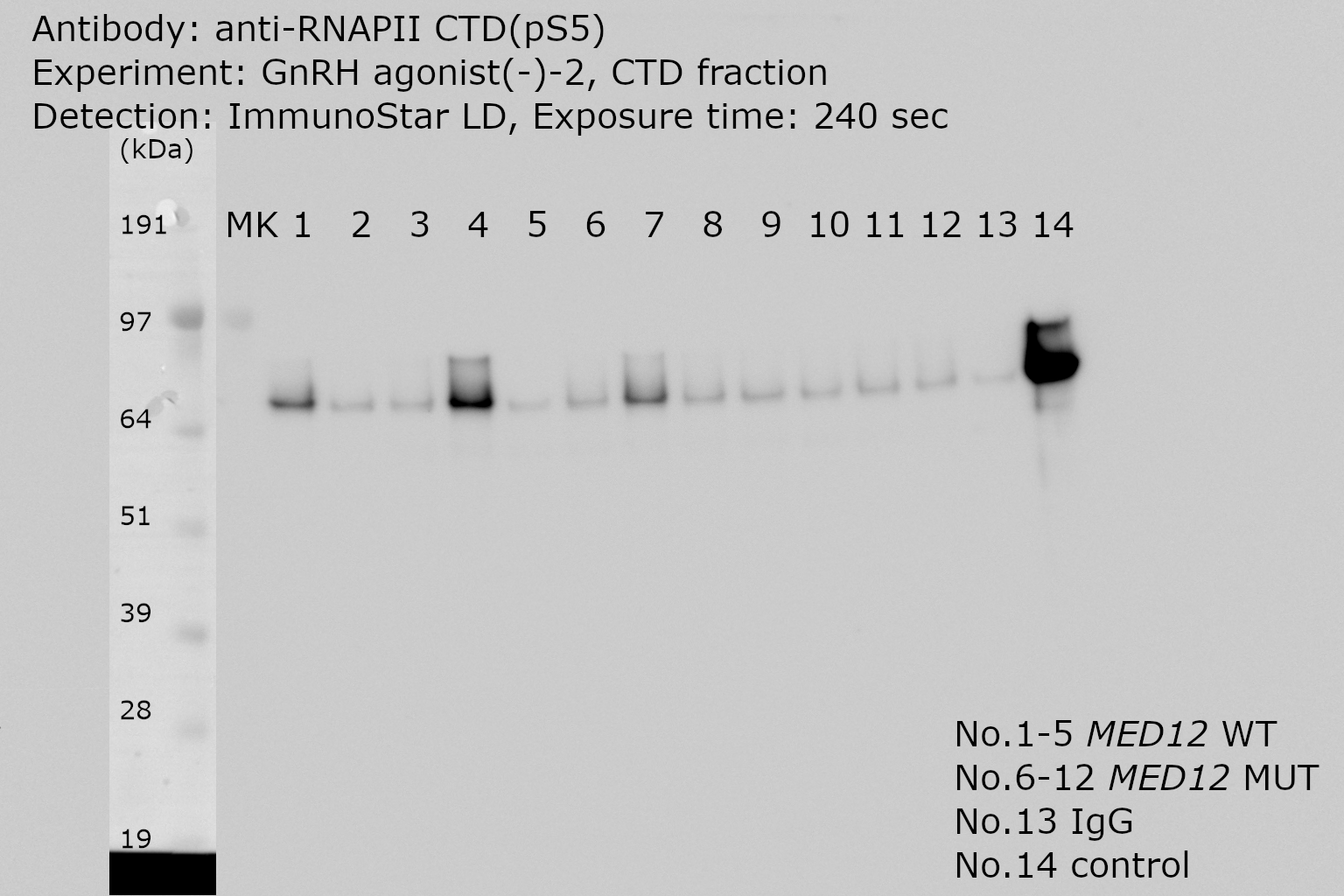

Supplement: S1 Raw Images — (ZIP) [file pone.0338485.s005.zip › CTD-pS5 GnRH-2.tif]

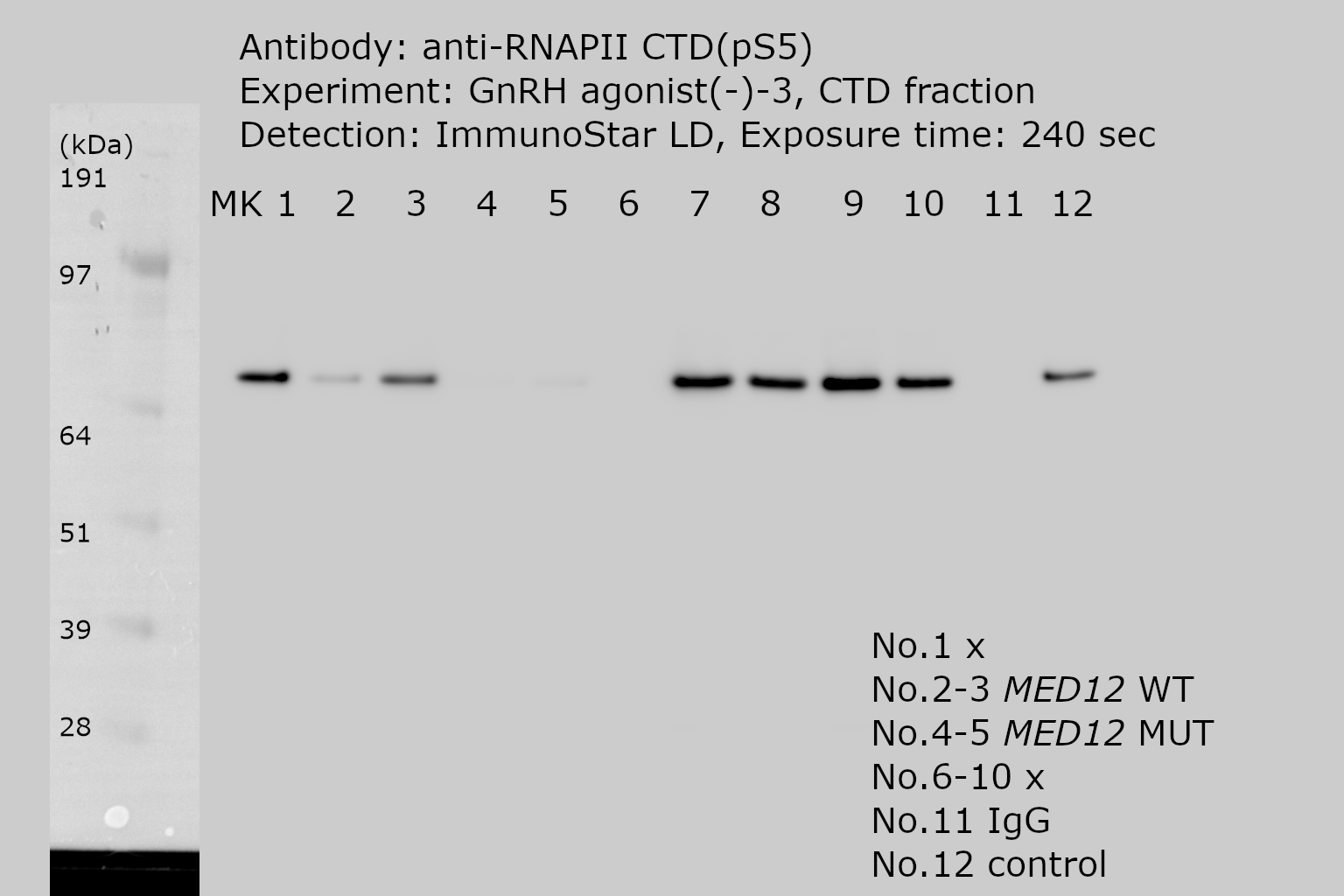

Supplement: S1 Raw Images — (ZIP) [file pone.0338485.s005.zip › CTD-pS5 GnRH-3.tif]

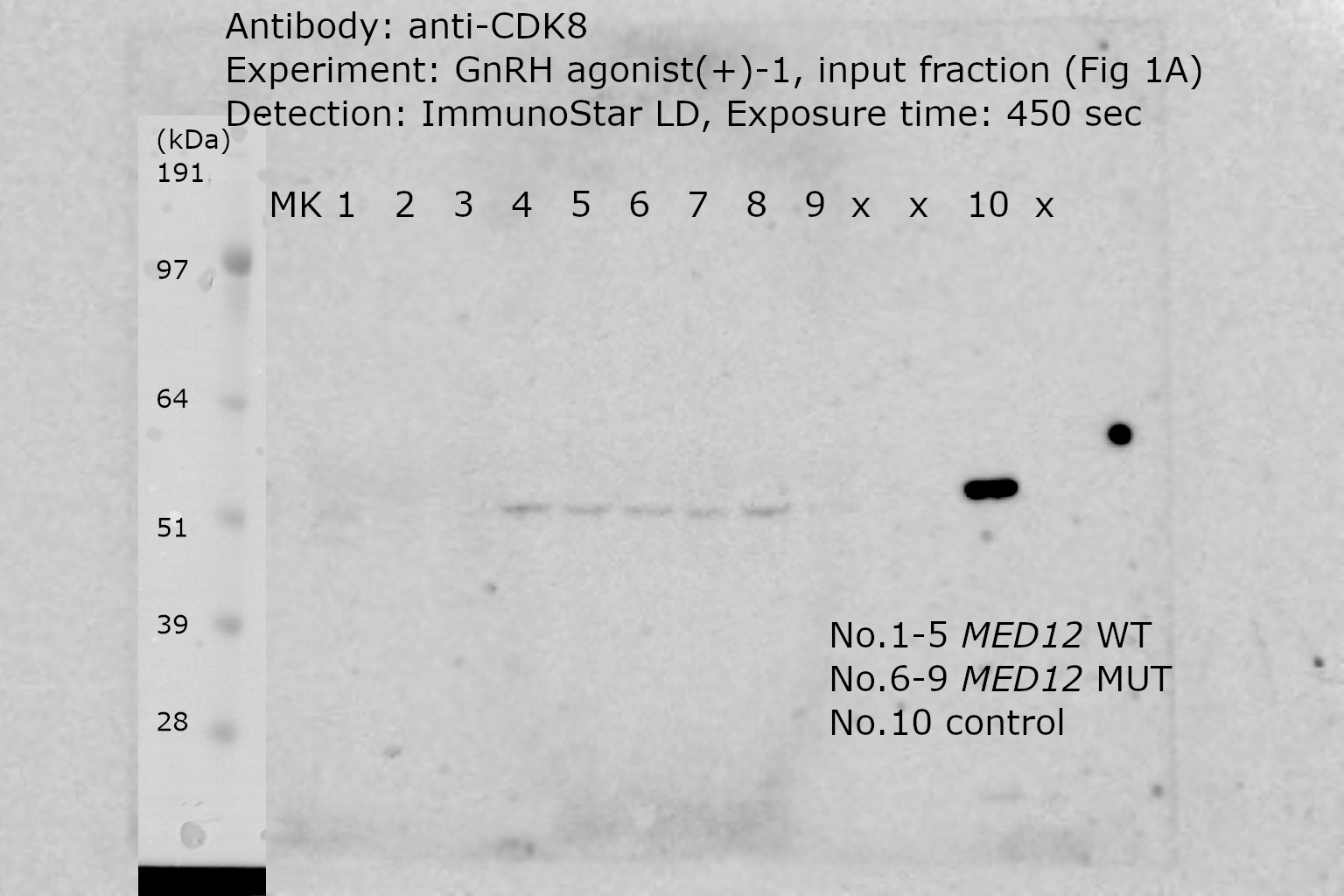

Supplement: S1 Raw Images — (ZIP) [file pone.0338485.s005.zip › input-CDK8 GnRH+1.tif]

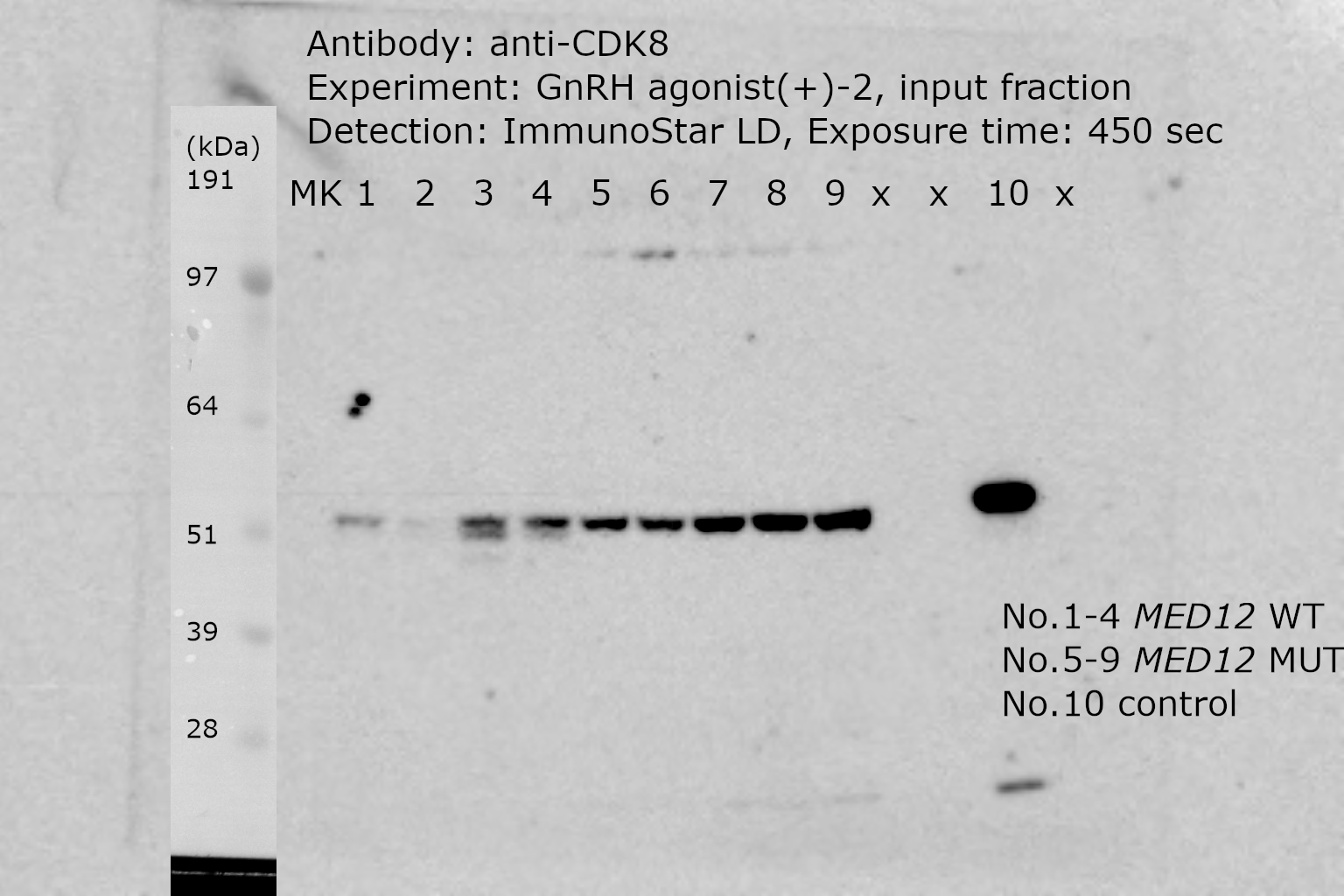

Supplement: S1 Raw Images — (ZIP) [file pone.0338485.s005.zip › input-CDK8 GnRH+2.tif]

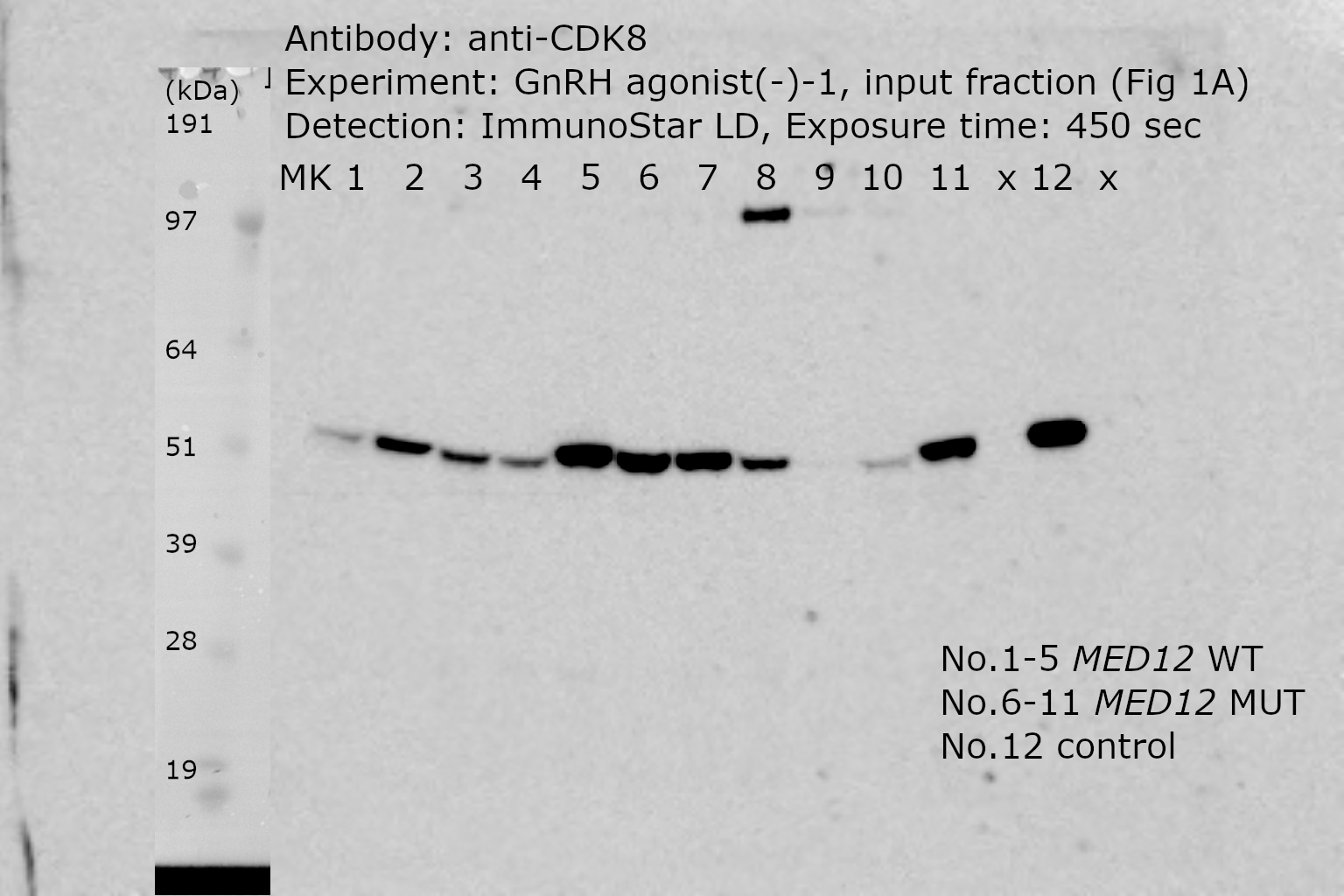

Supplement: S1 Raw Images — (ZIP) [file pone.0338485.s005.zip › input-CDK8 GnRH-1.tif]

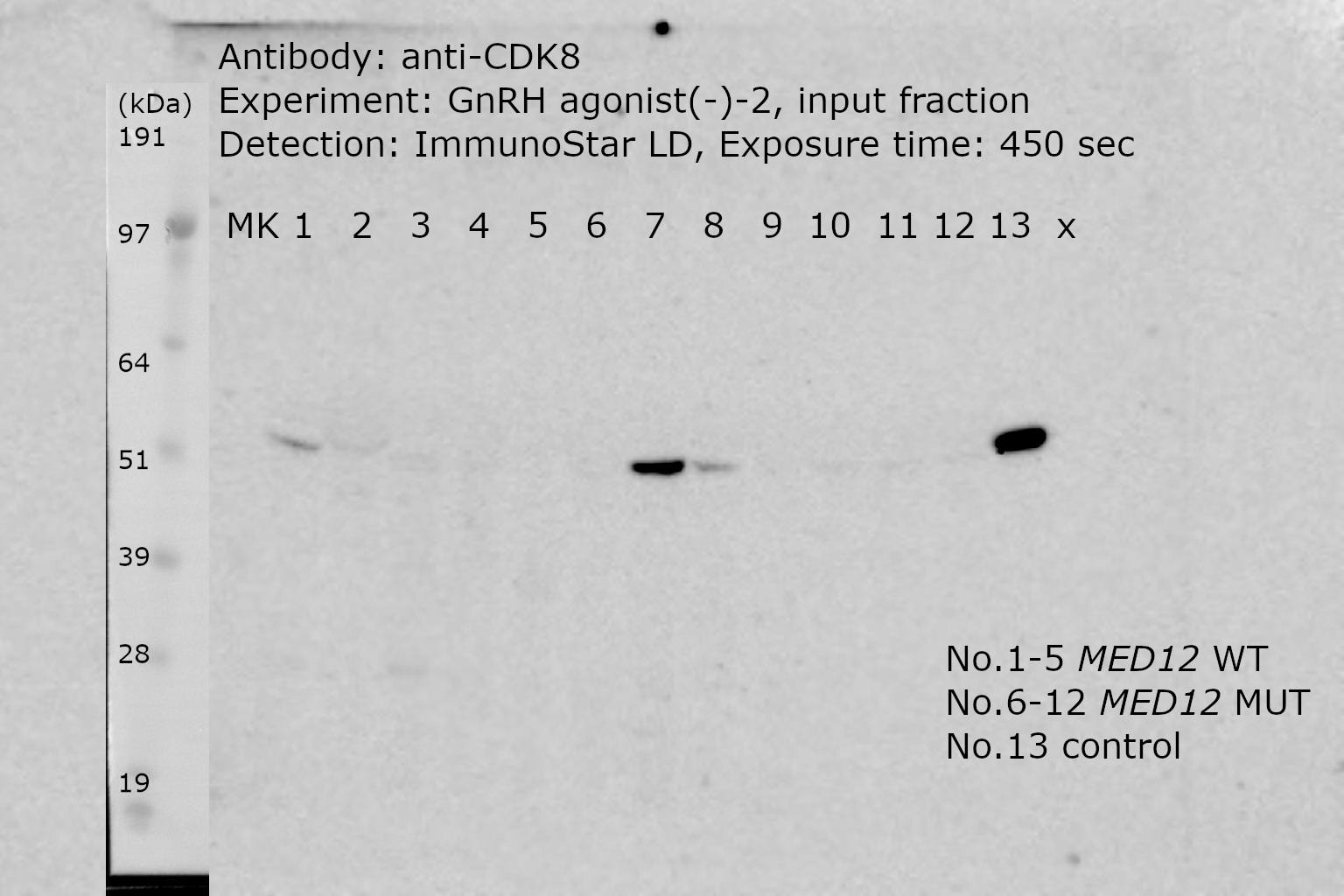

Supplement: S1 Raw Images — (ZIP) [file pone.0338485.s005.zip › input-CDK8 GnRH-2.tif]

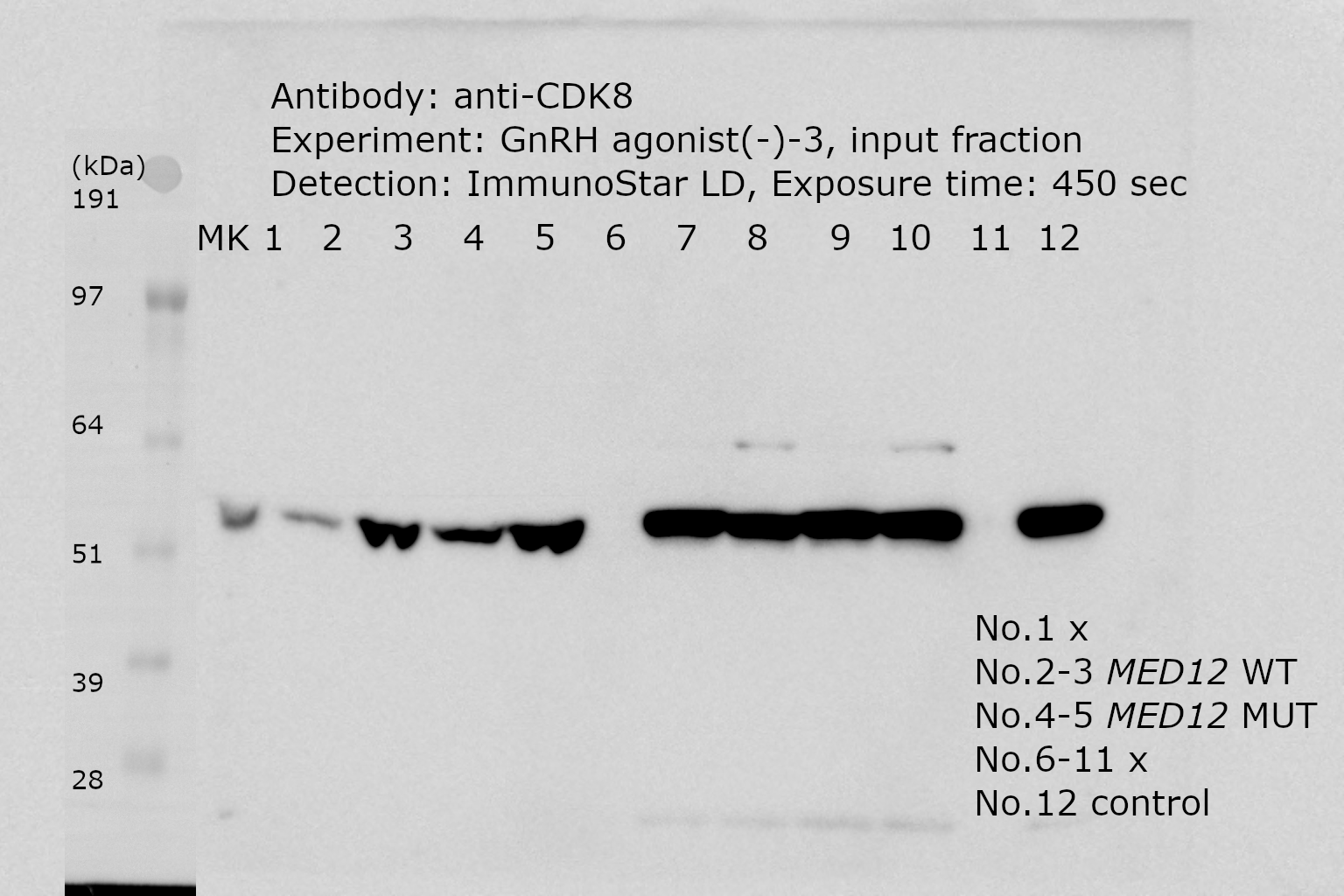

Supplement: S1 Raw Images — (ZIP) [file pone.0338485.s005.zip › input-CDK8 GnRH-3.tif]

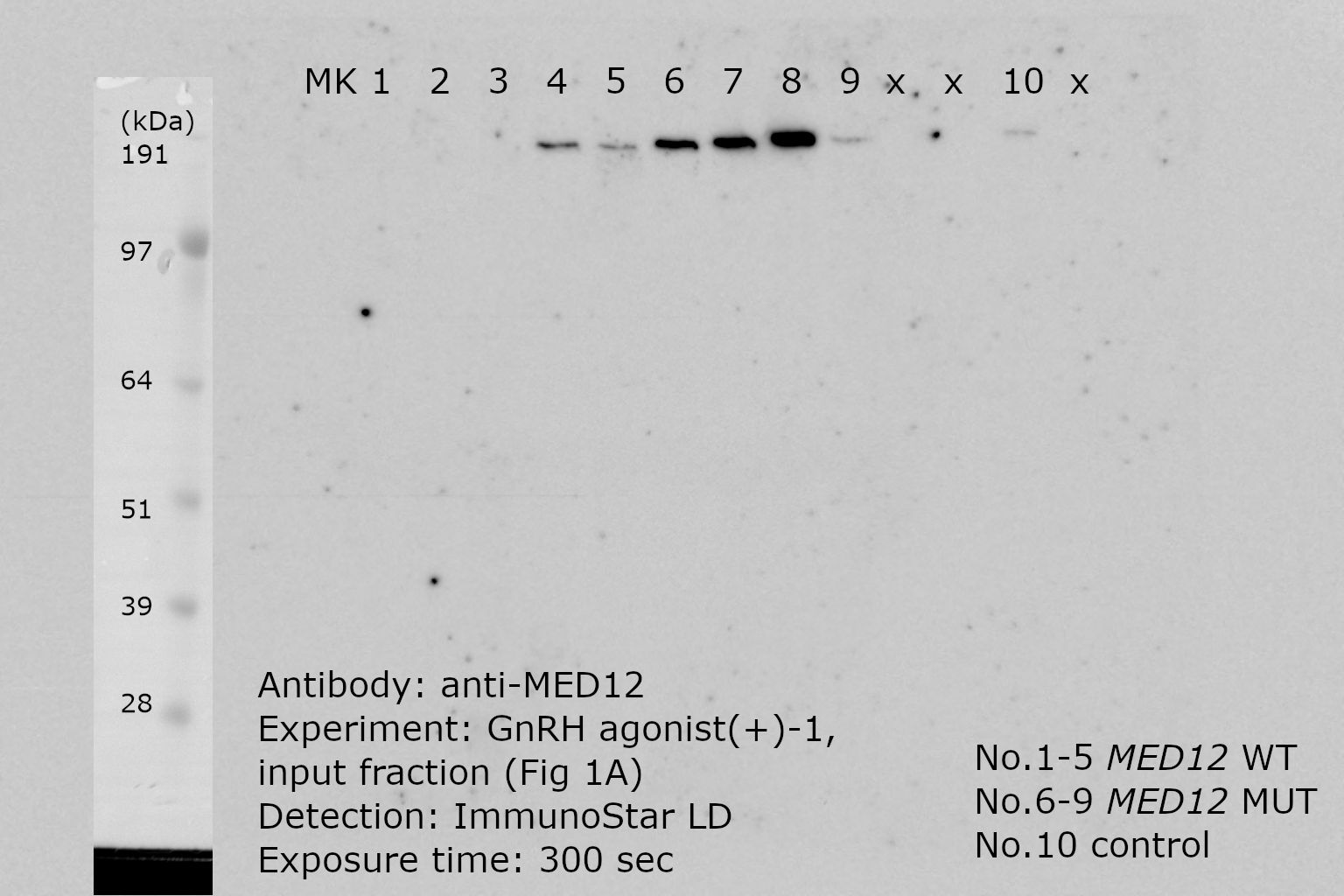

Supplement: S1 Raw Images — (ZIP) [file pone.0338485.s005.zip › input-MED12 GnRH+1.tif]

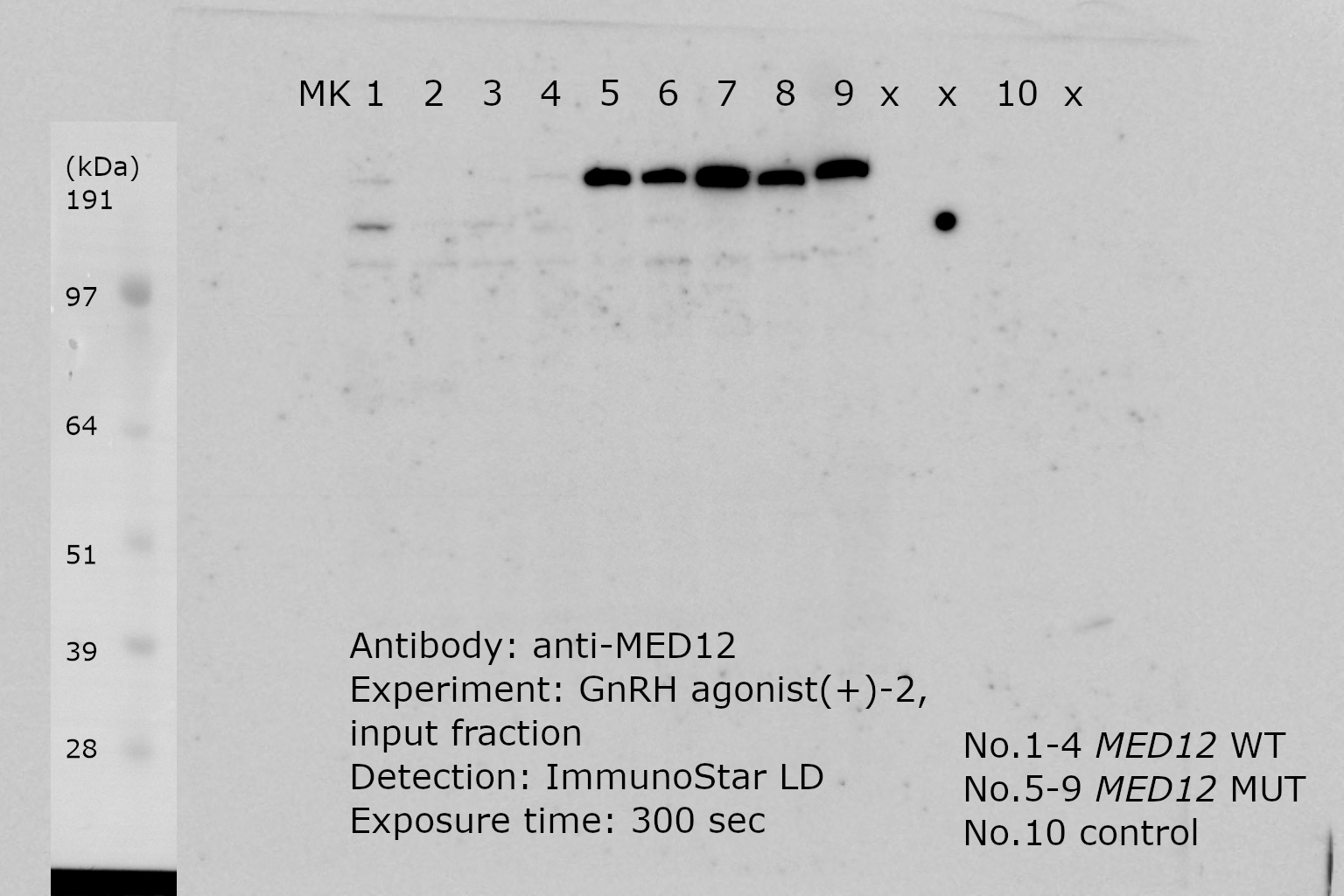

Supplement: S1 Raw Images — (ZIP) [file pone.0338485.s005.zip › input-MED12 GnRH+2.tif]

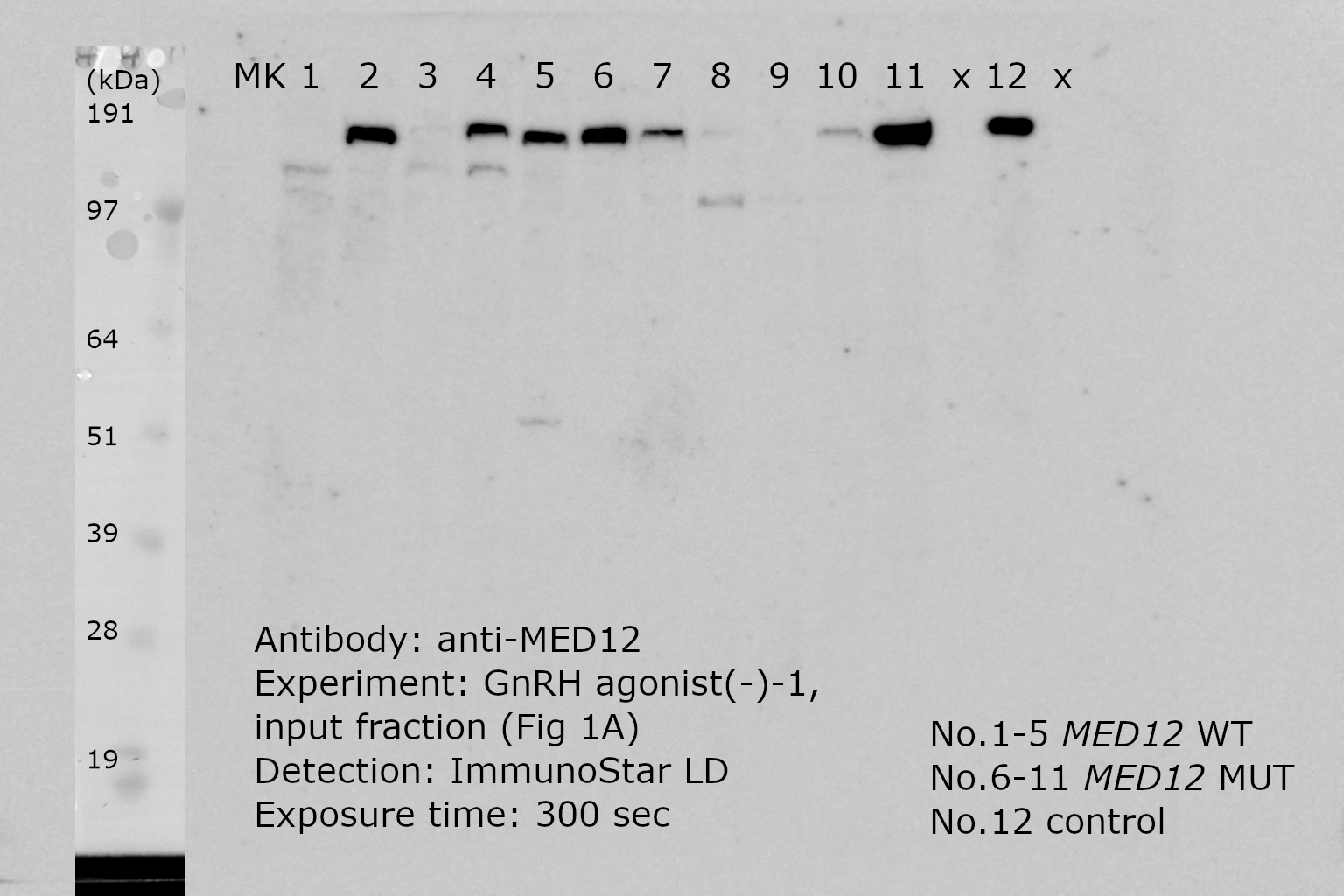

Supplement: S1 Raw Images — (ZIP) [file pone.0338485.s005.zip › input-MED12 GnRH-1.tif]

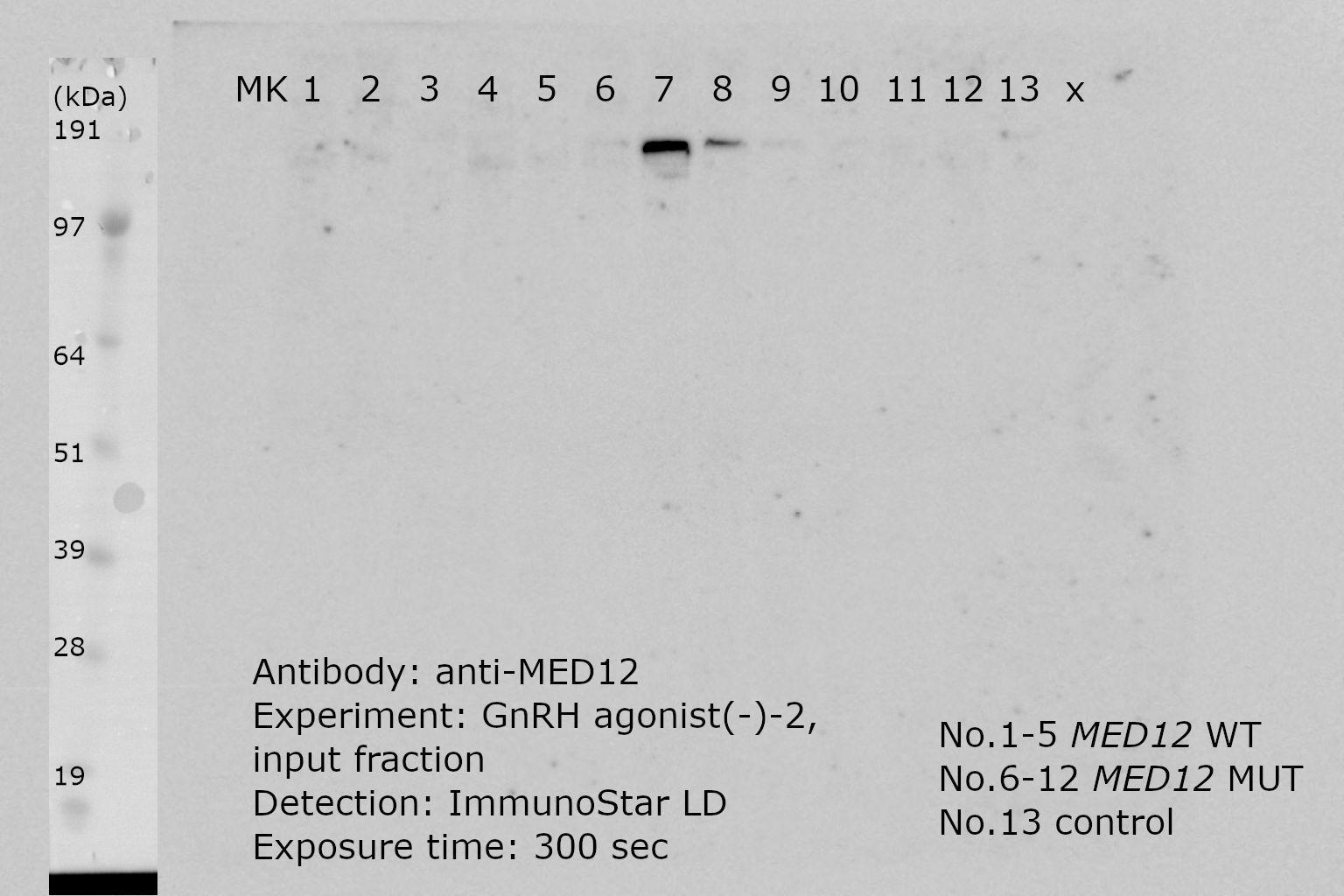

Supplement: S1 Raw Images — (ZIP) [file pone.0338485.s005.zip › input-MED12 GnRH-2.tif]

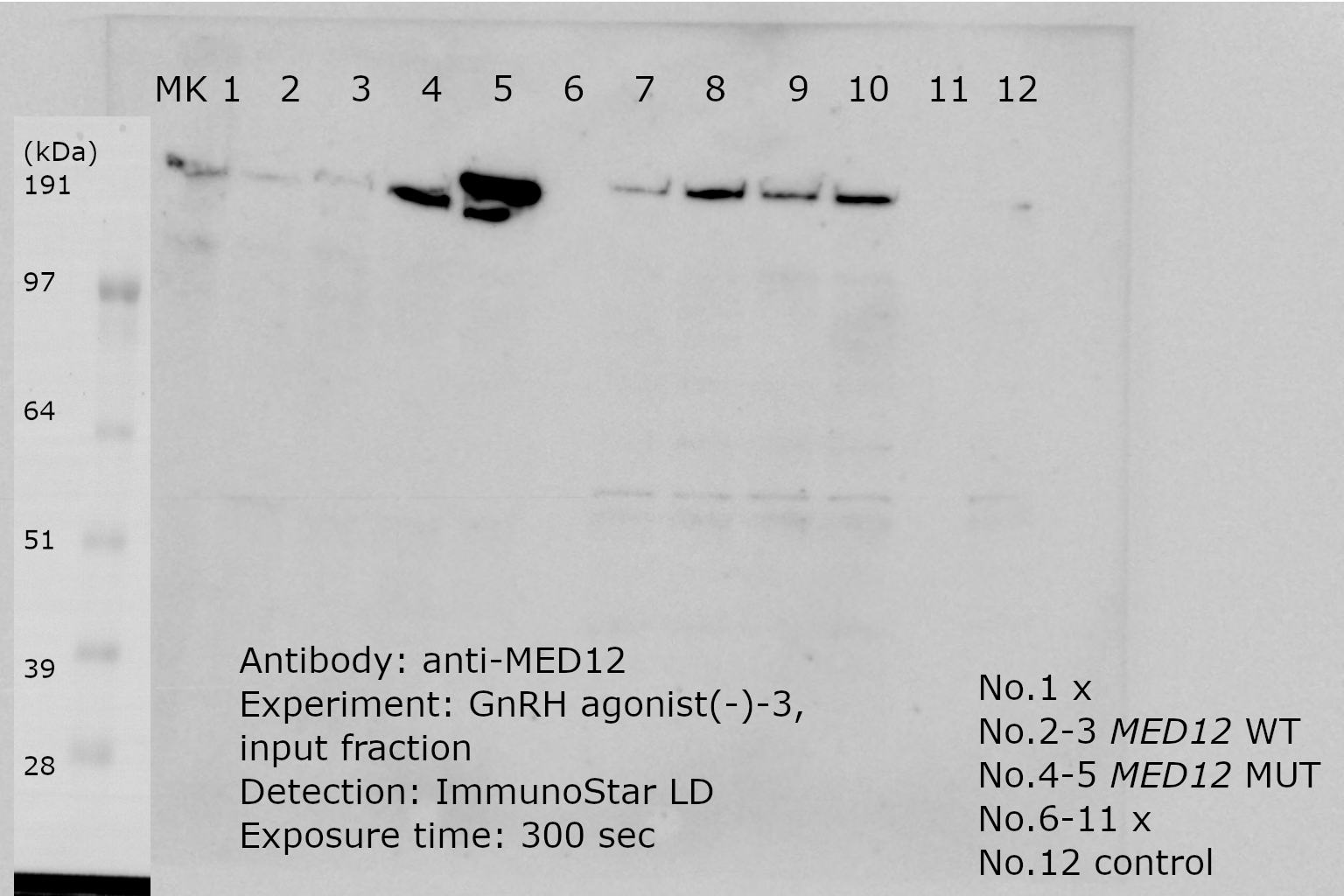

Supplement: S1 Raw Images — (ZIP) [file pone.0338485.s005.zip › input-MED12 GnRH-3.tif]

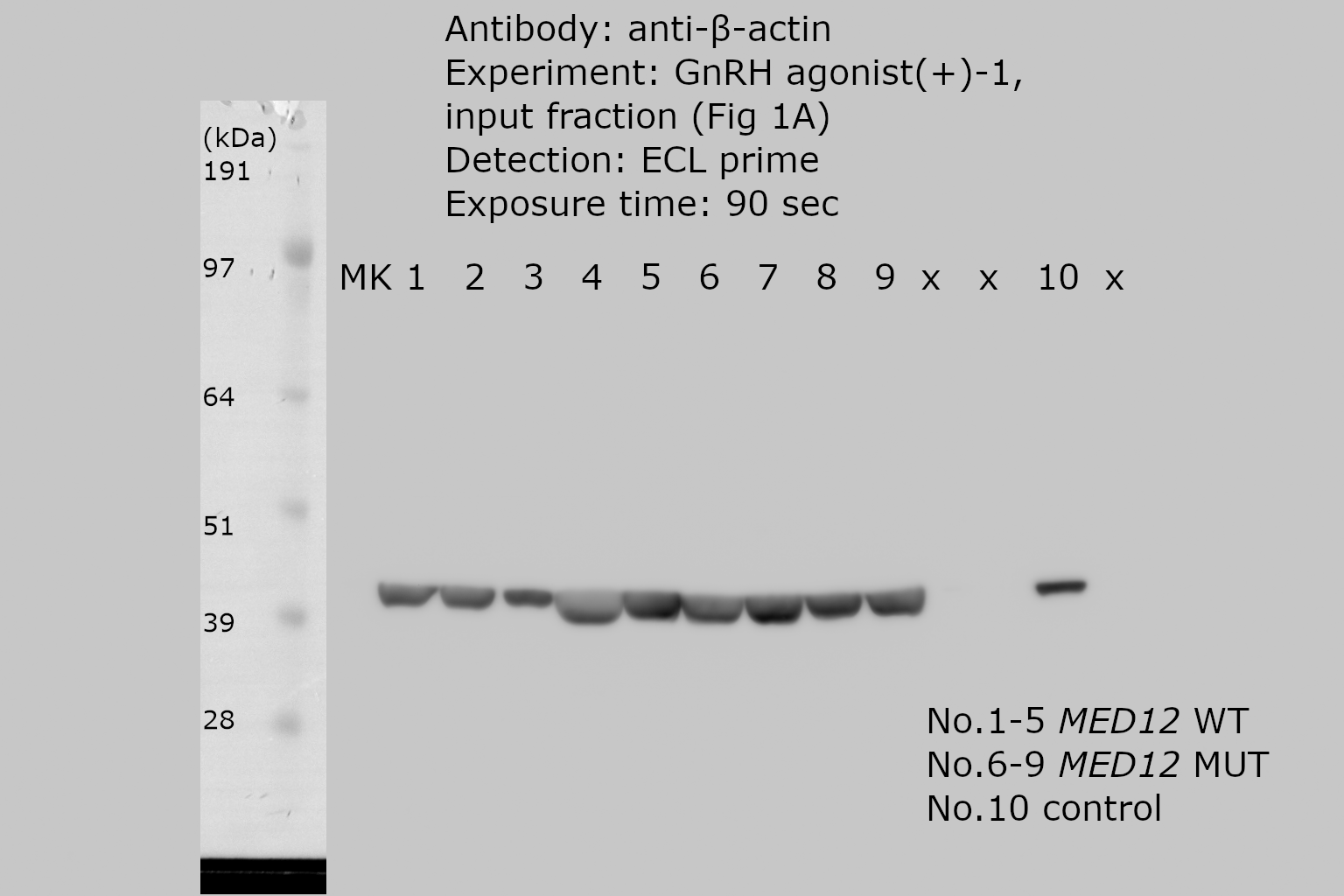

Supplement: S1 Raw Images — (ZIP) [file pone.0338485.s005.zip › input-βactin GnRH+1.tif]

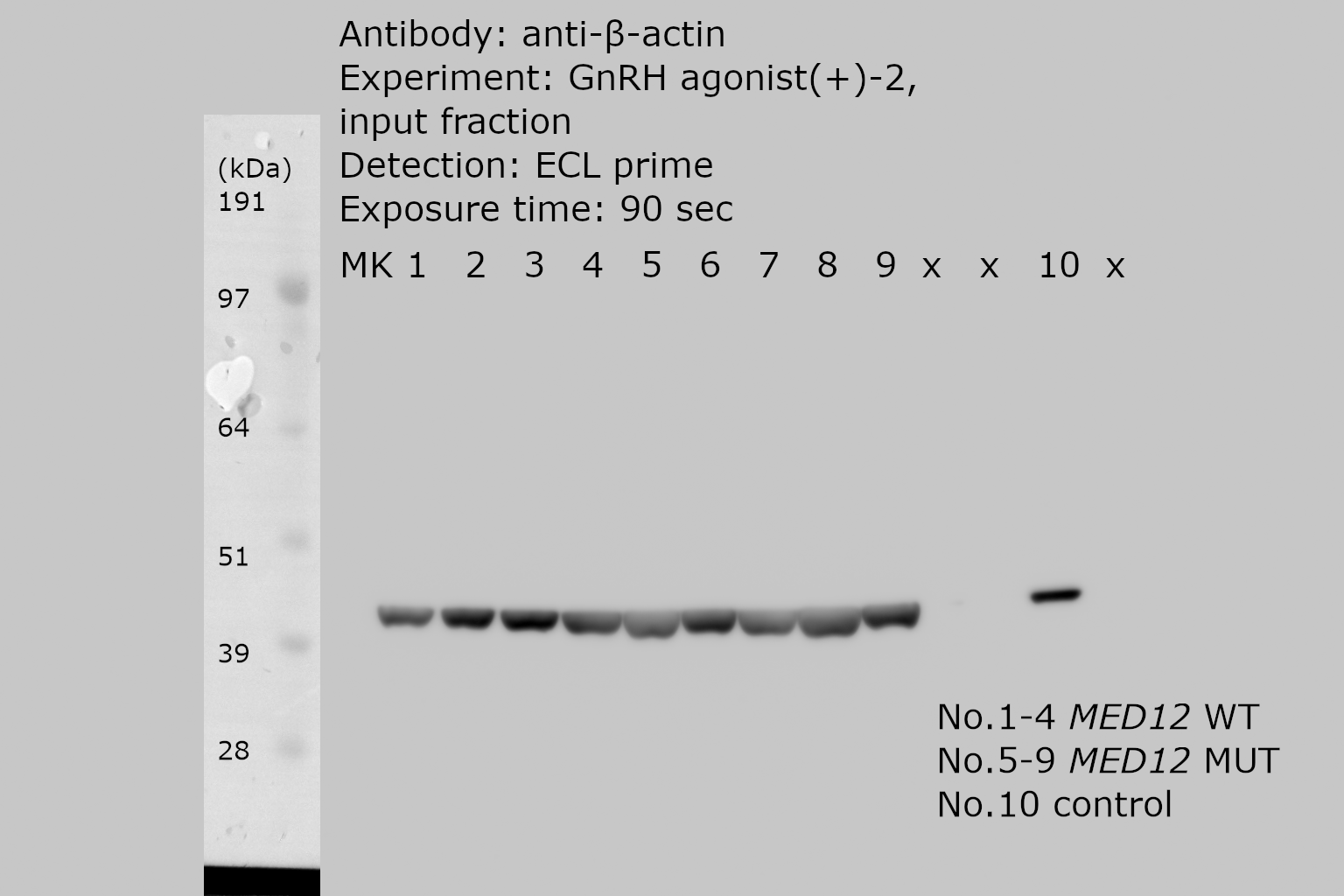

Supplement: S1 Raw Images — (ZIP) [file pone.0338485.s005.zip › input-βactin GnRH+2.tif]

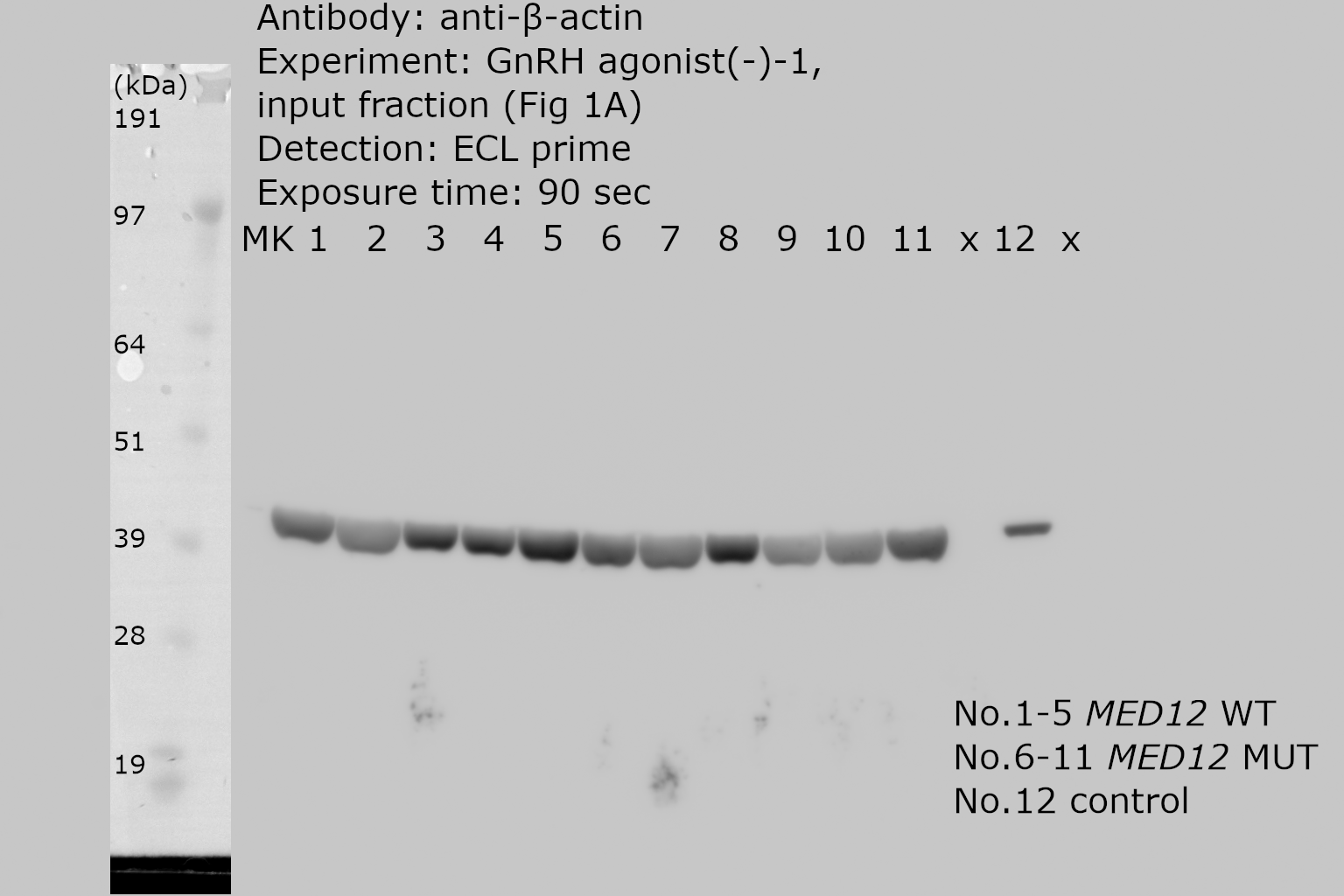

Supplement: S1 Raw Images — (ZIP) [file pone.0338485.s005.zip › input-βactin GnRH-1.tif]

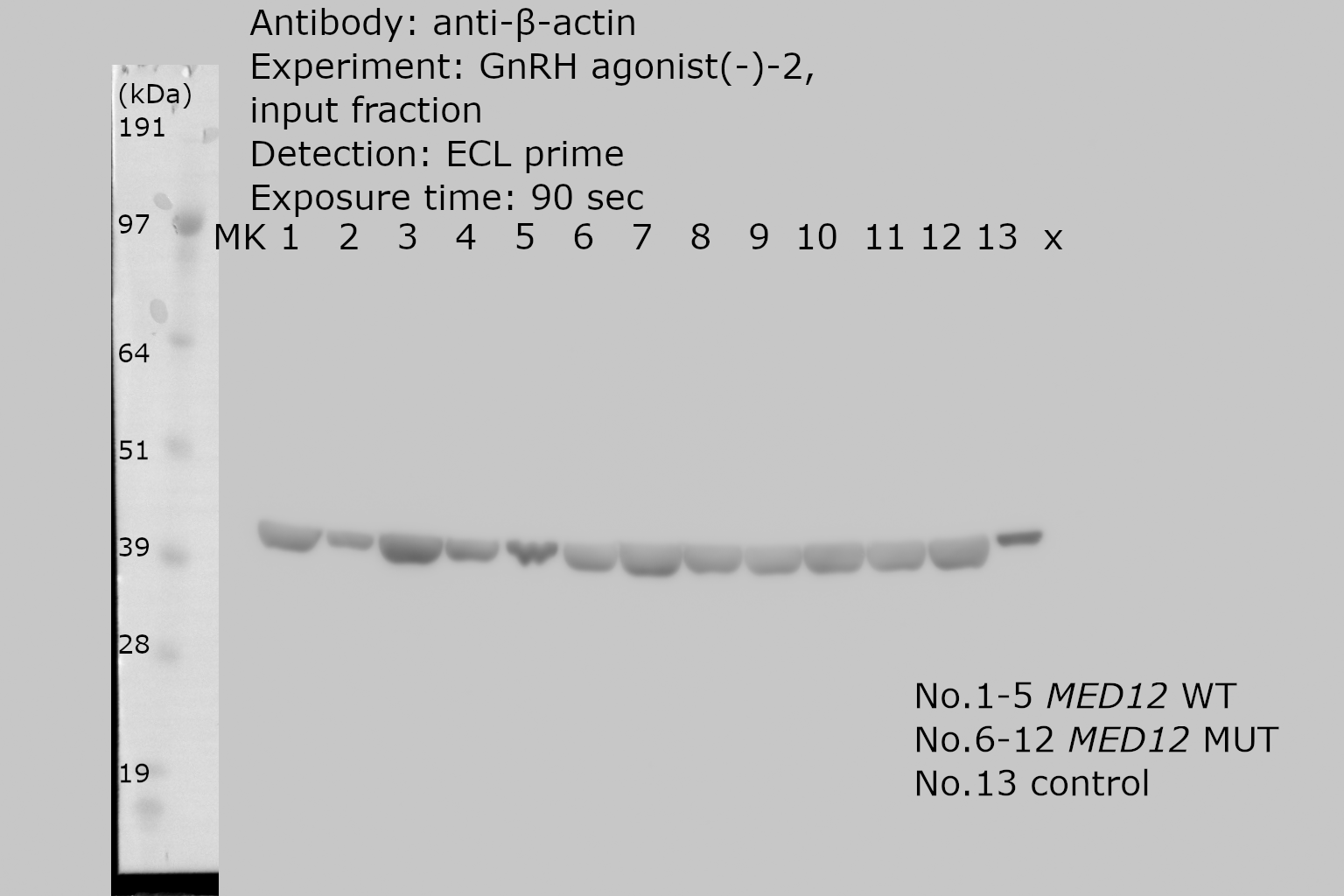

Supplement: S1 Raw Images — (ZIP) [file pone.0338485.s005.zip › input-βactin GnRH-2.tif]

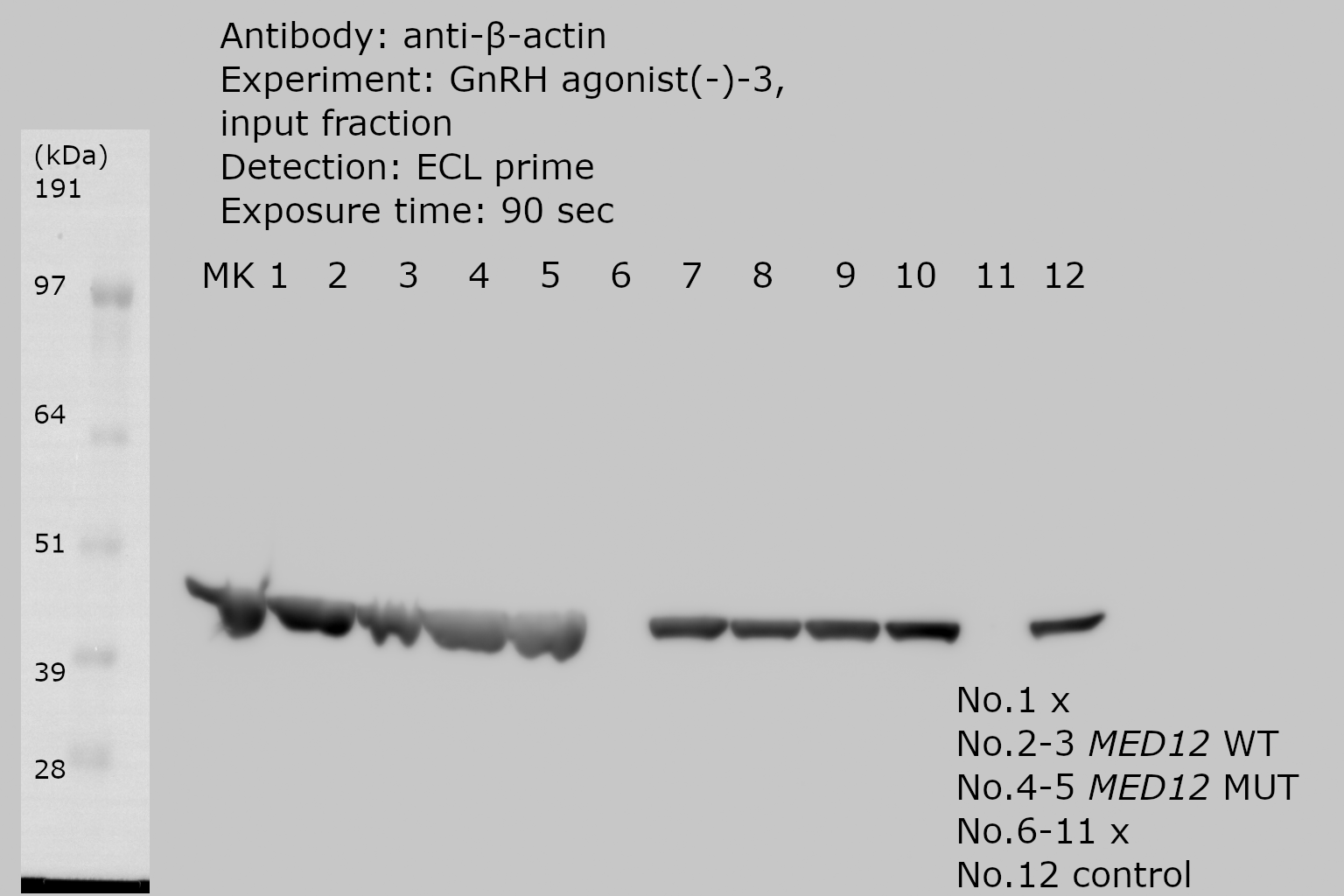

Supplement: S1 Raw Images — (ZIP) [file pone.0338485.s005.zip › input-βactin GnRH-3.tif]

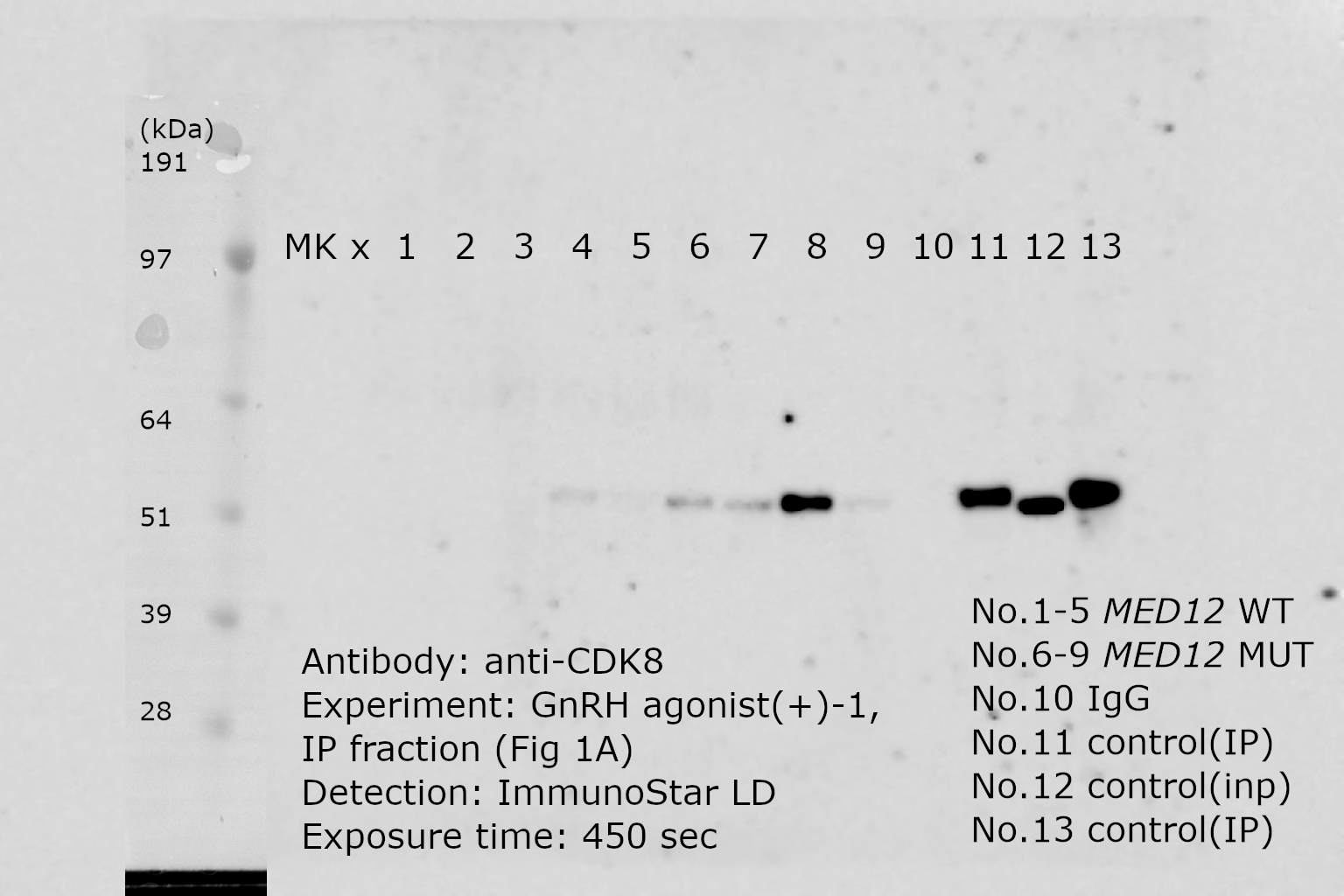

Supplement: S1 Raw Images — (ZIP) [file pone.0338485.s005.zip › IP-CDK8 GnRH+1.tif]

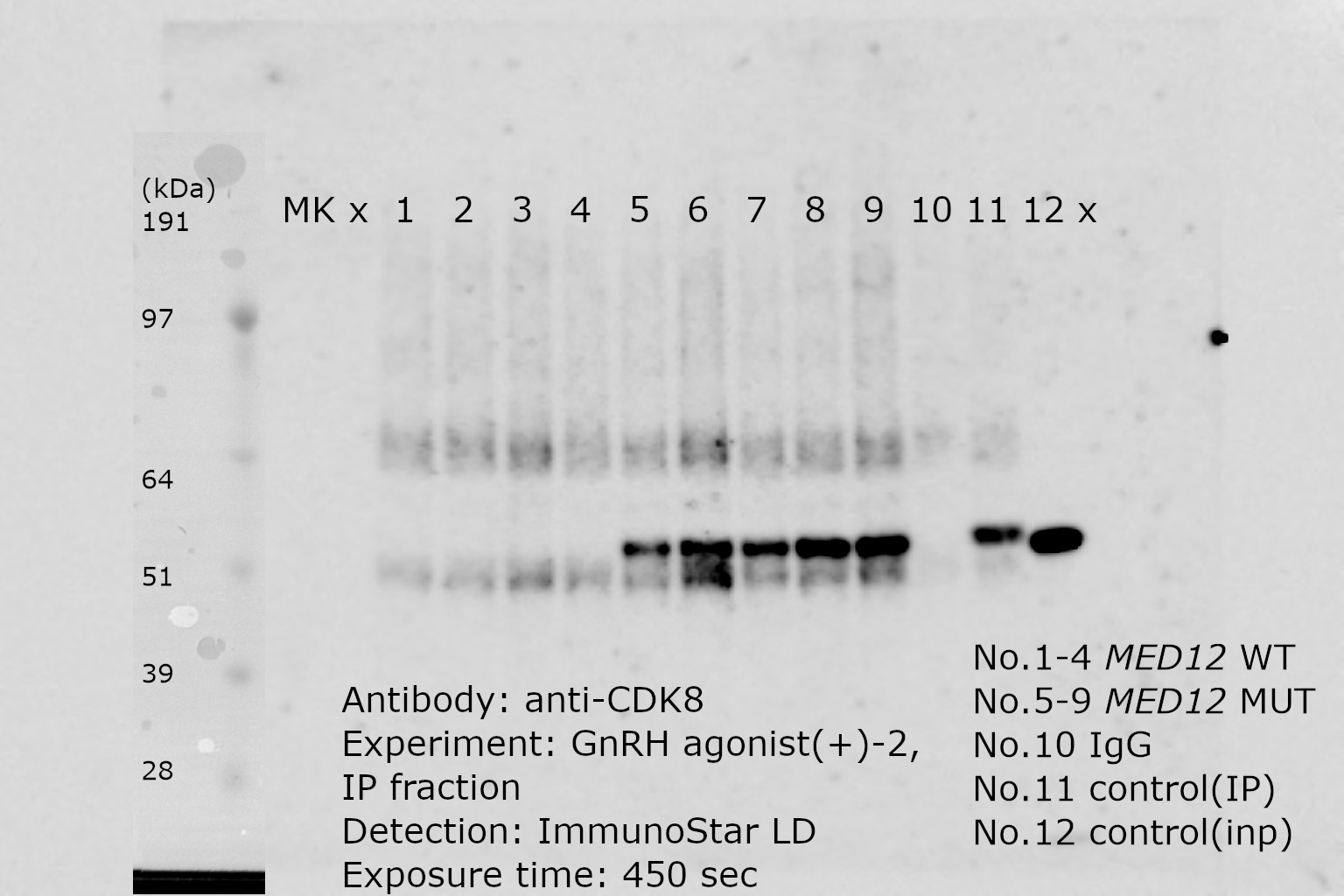

Supplement: S1 Raw Images — (ZIP) [file pone.0338485.s005.zip › IP-CDK8 GnRH+2.tif]

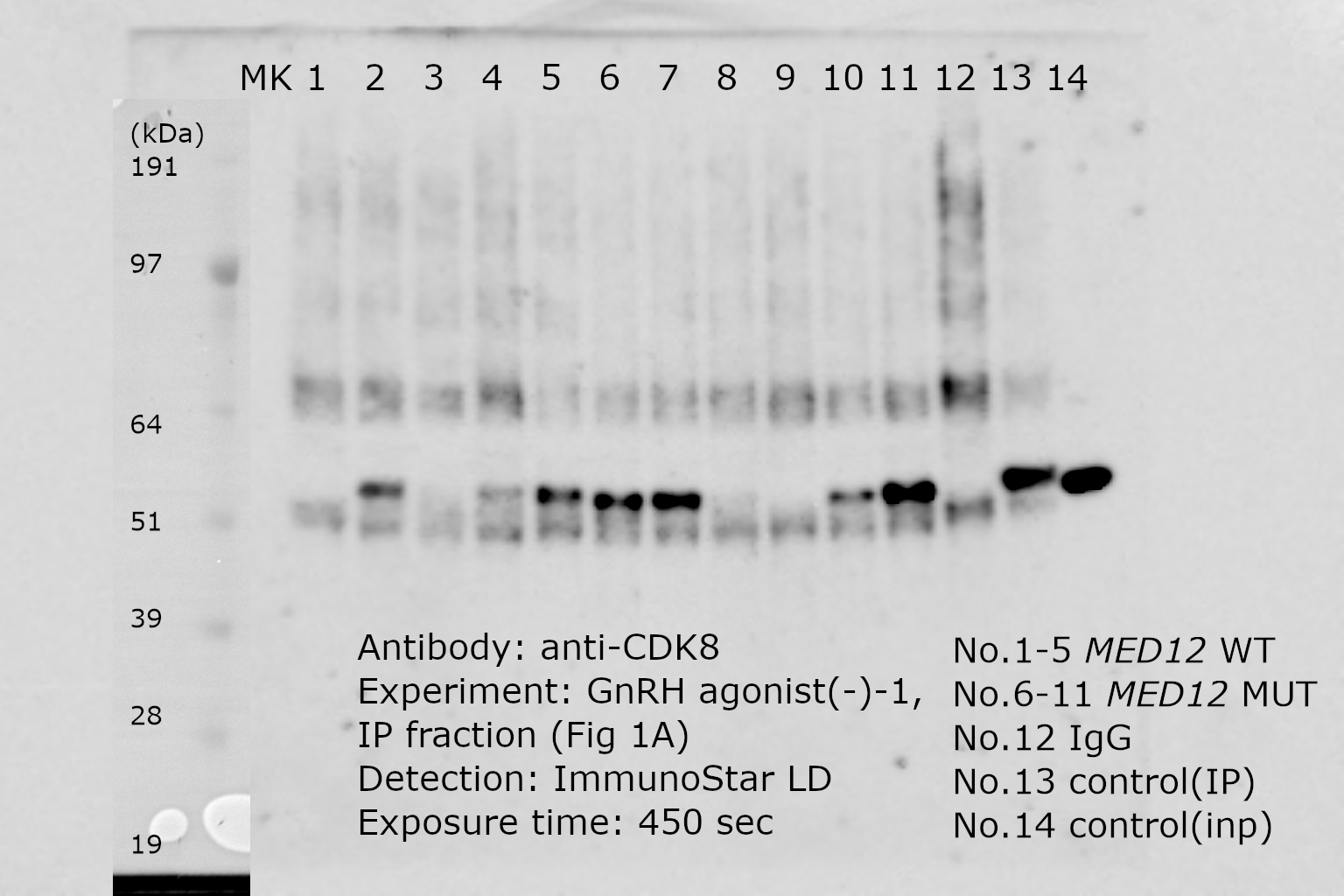

Supplement: S1 Raw Images — (ZIP) [file pone.0338485.s005.zip › IP-CDK8 GnRH-1.tif]

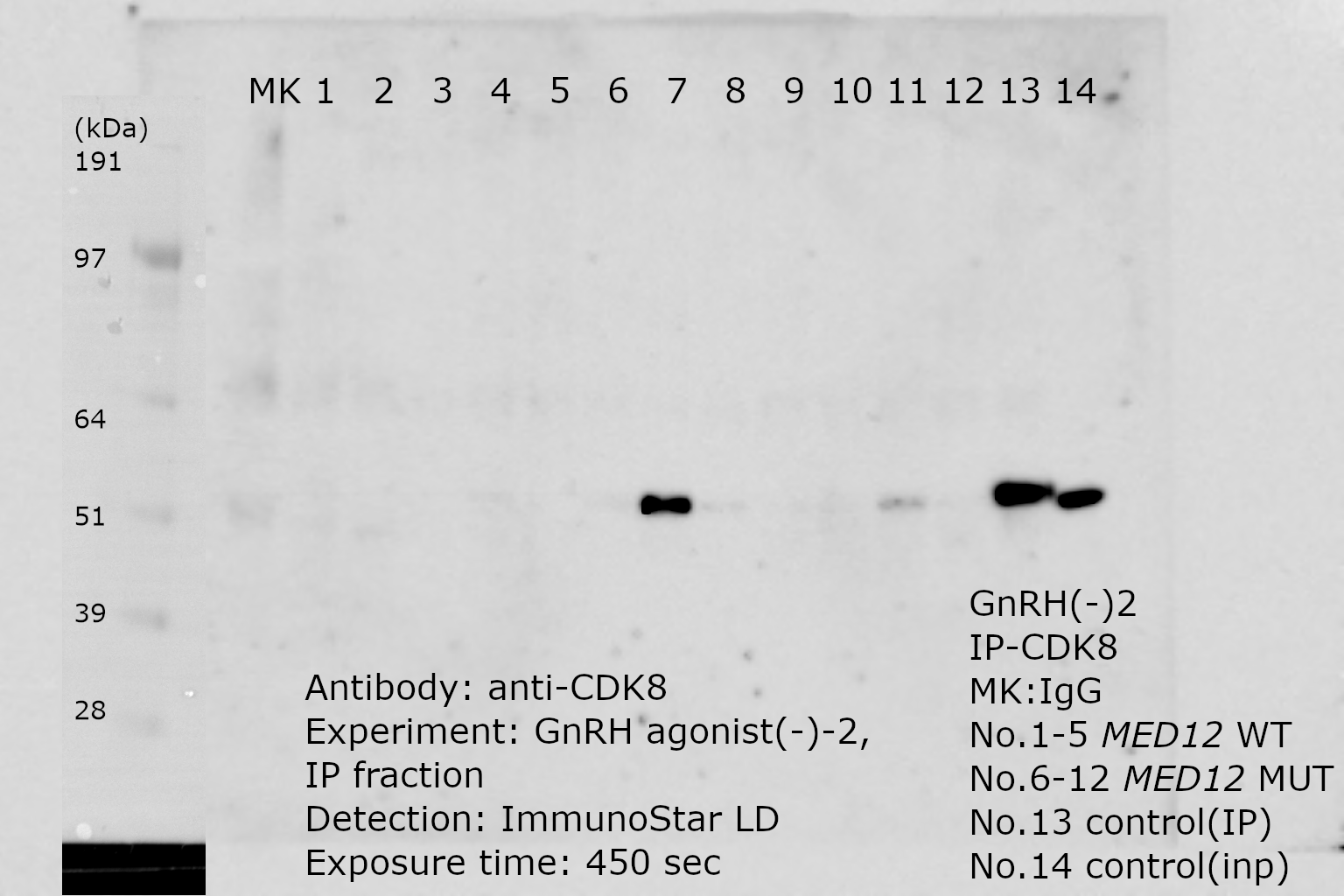

Supplement: S1 Raw Images — (ZIP) [file pone.0338485.s005.zip › IP-CDK8 GnRH-2.tif]

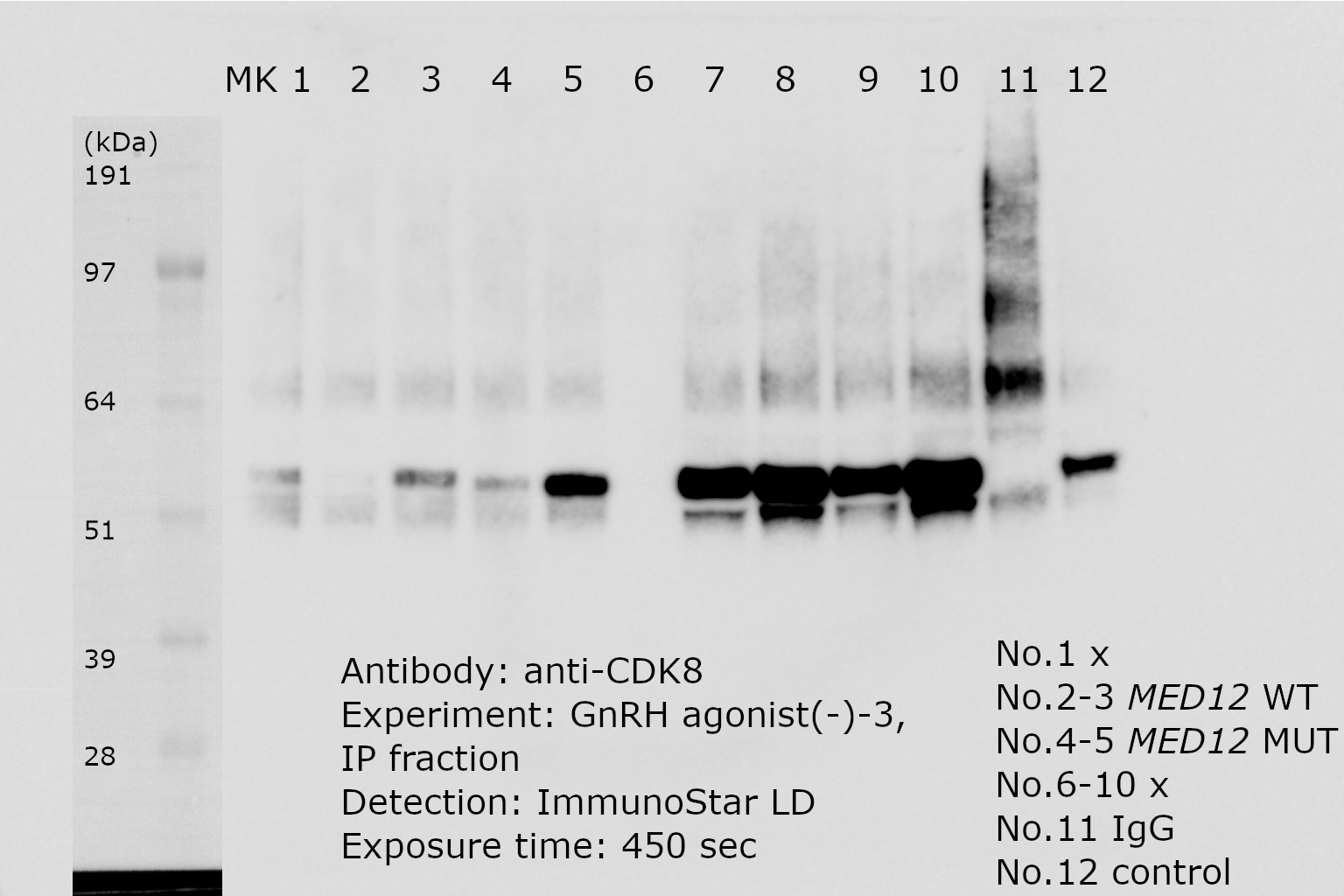

Supplement: S1 Raw Images — (ZIP) [file pone.0338485.s005.zip › IP-CDK8 GnRH-3.tif]

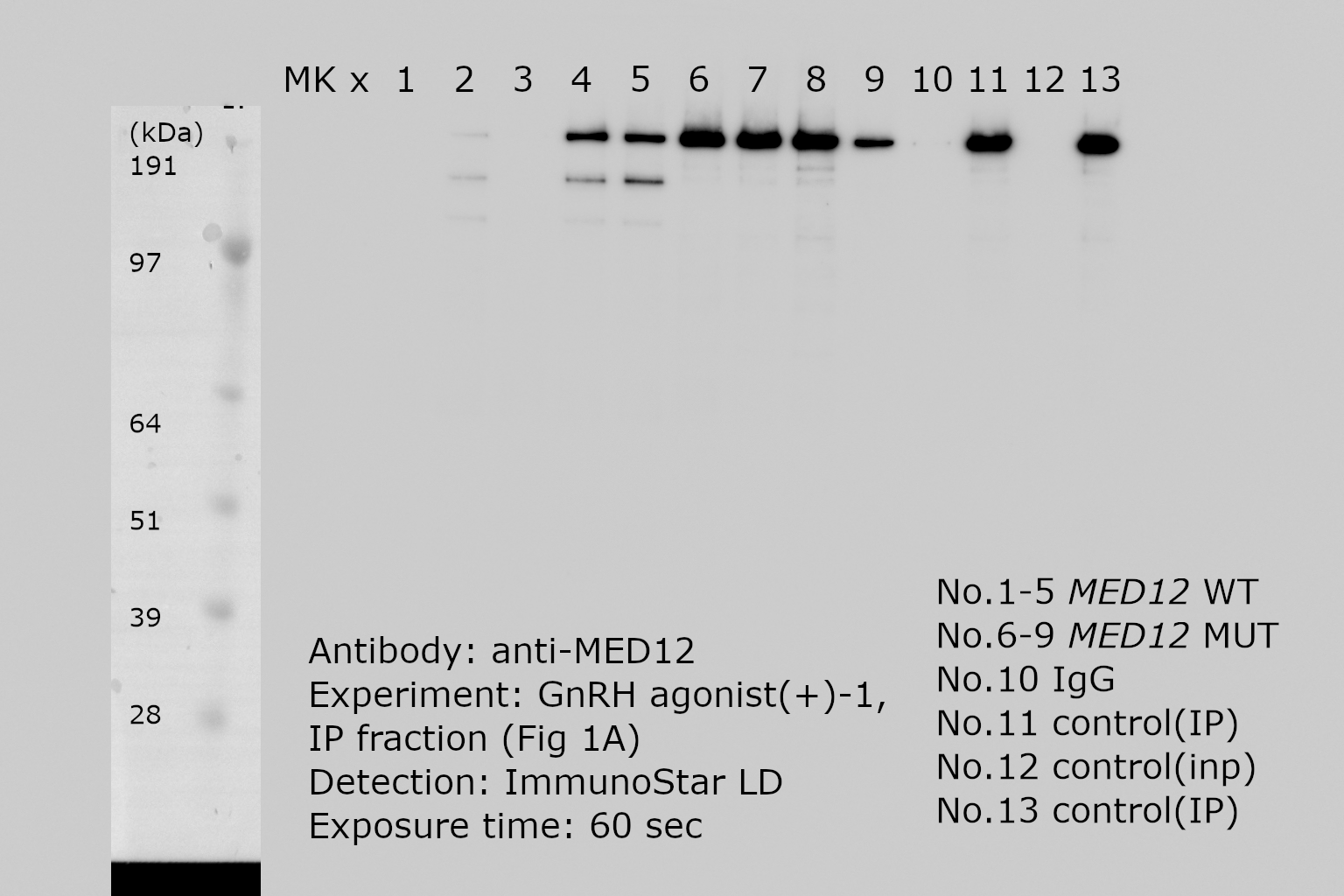

Supplement: S1 Raw Images — (ZIP) [file pone.0338485.s005.zip › IP-MED12 GnRH+1.tif]

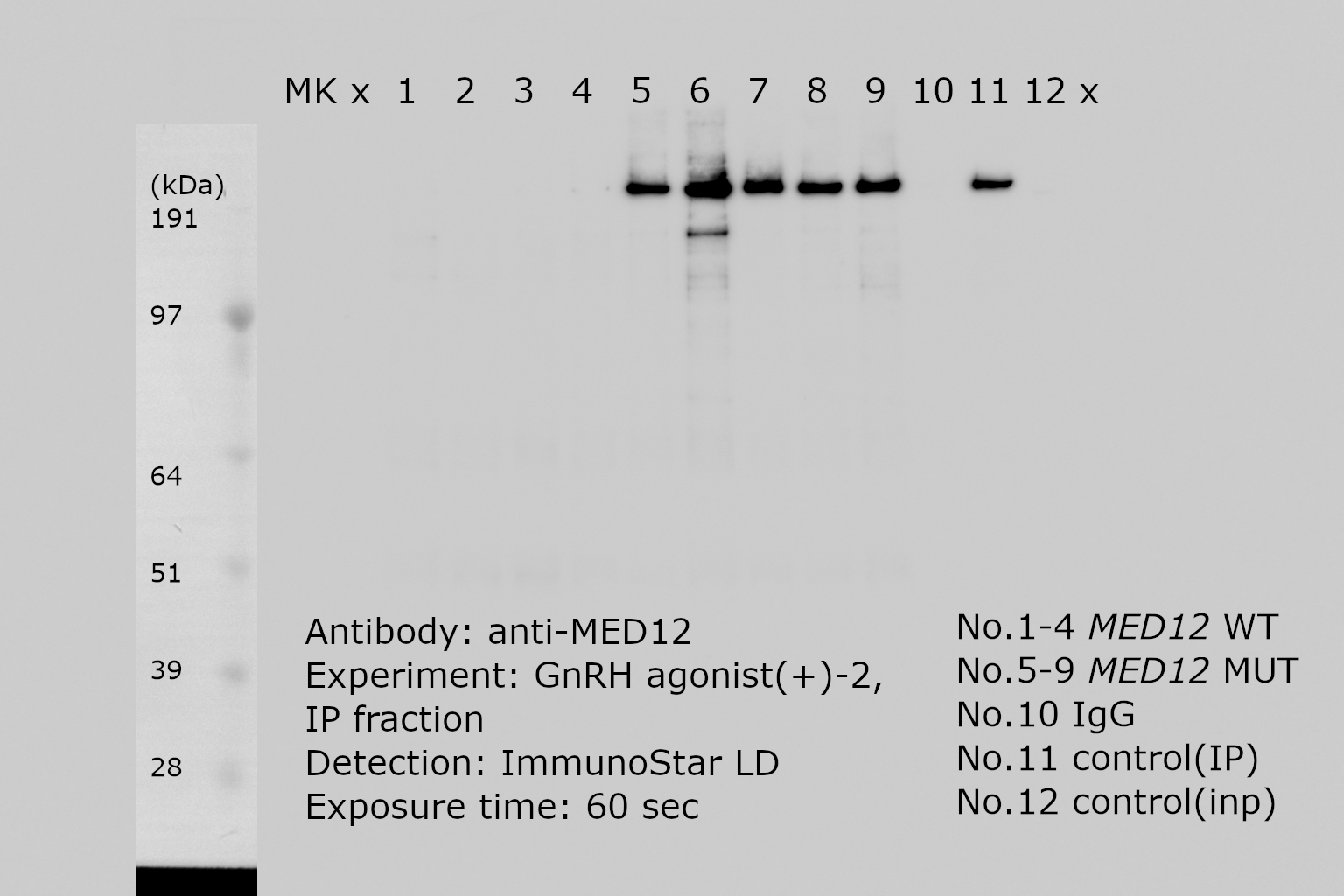

Supplement: S1 Raw Images — (ZIP) [file pone.0338485.s005.zip › IP-MED12 GnRH+2.tif]

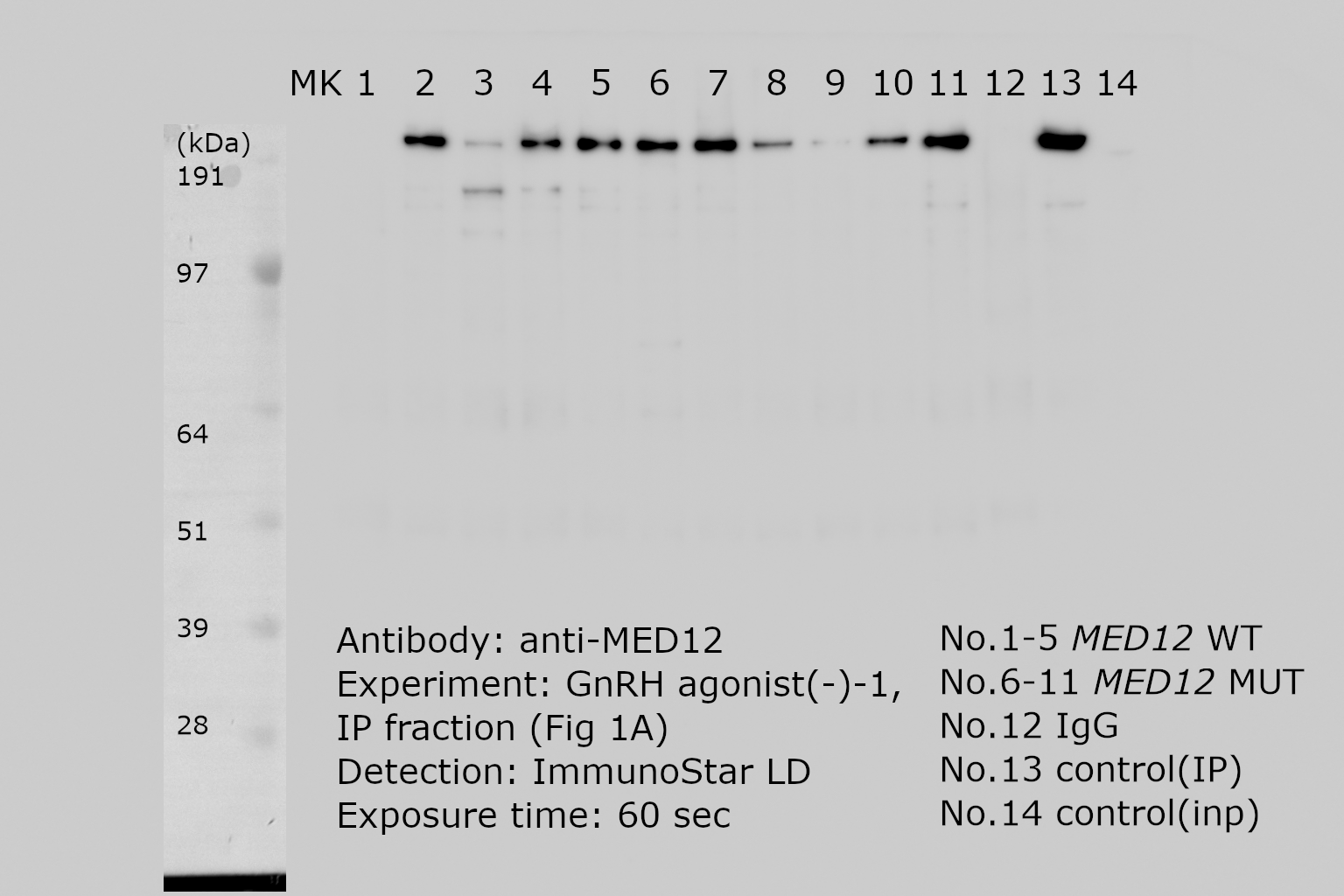

Supplement: S1 Raw Images — (ZIP) [file pone.0338485.s005.zip › IP-MED12 GnRH-1.tif]

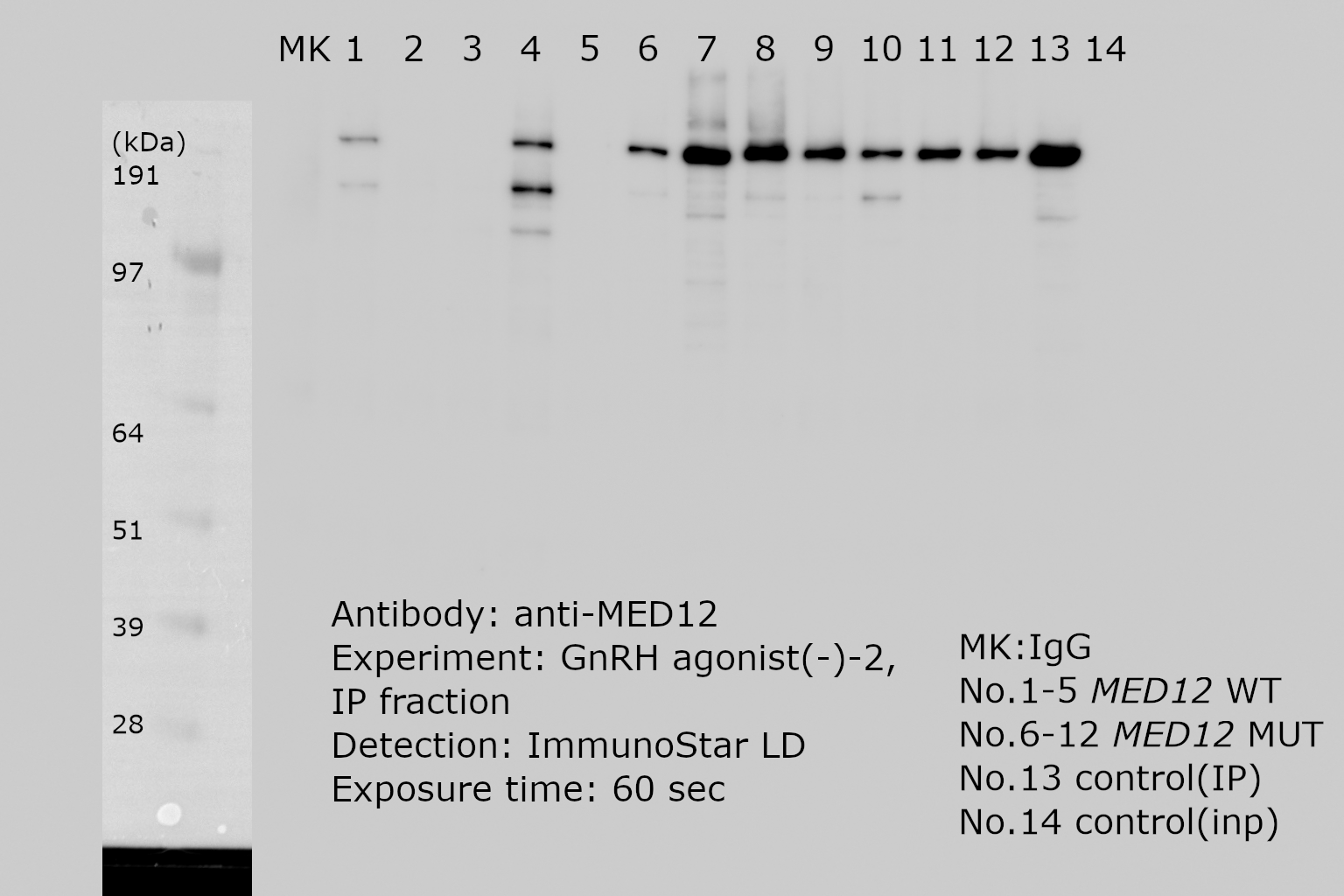

Supplement: S1 Raw Images — (ZIP) [file pone.0338485.s005.zip › IP-MED12 GnRH-2.tif]

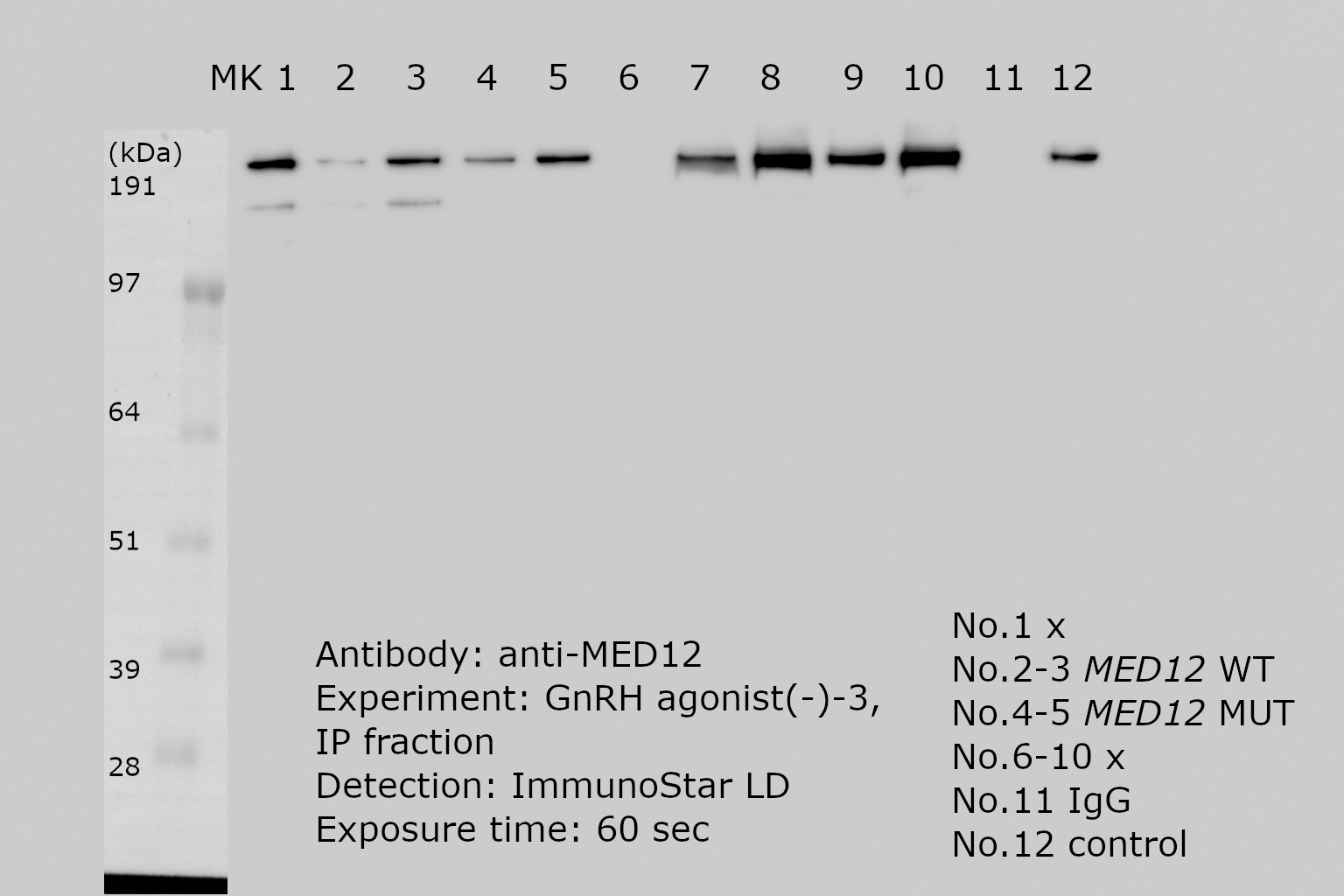

Supplement: S1 Raw Images — (ZIP) [file pone.0338485.s005.zip › IP-MED12 GnRH-3.tif]

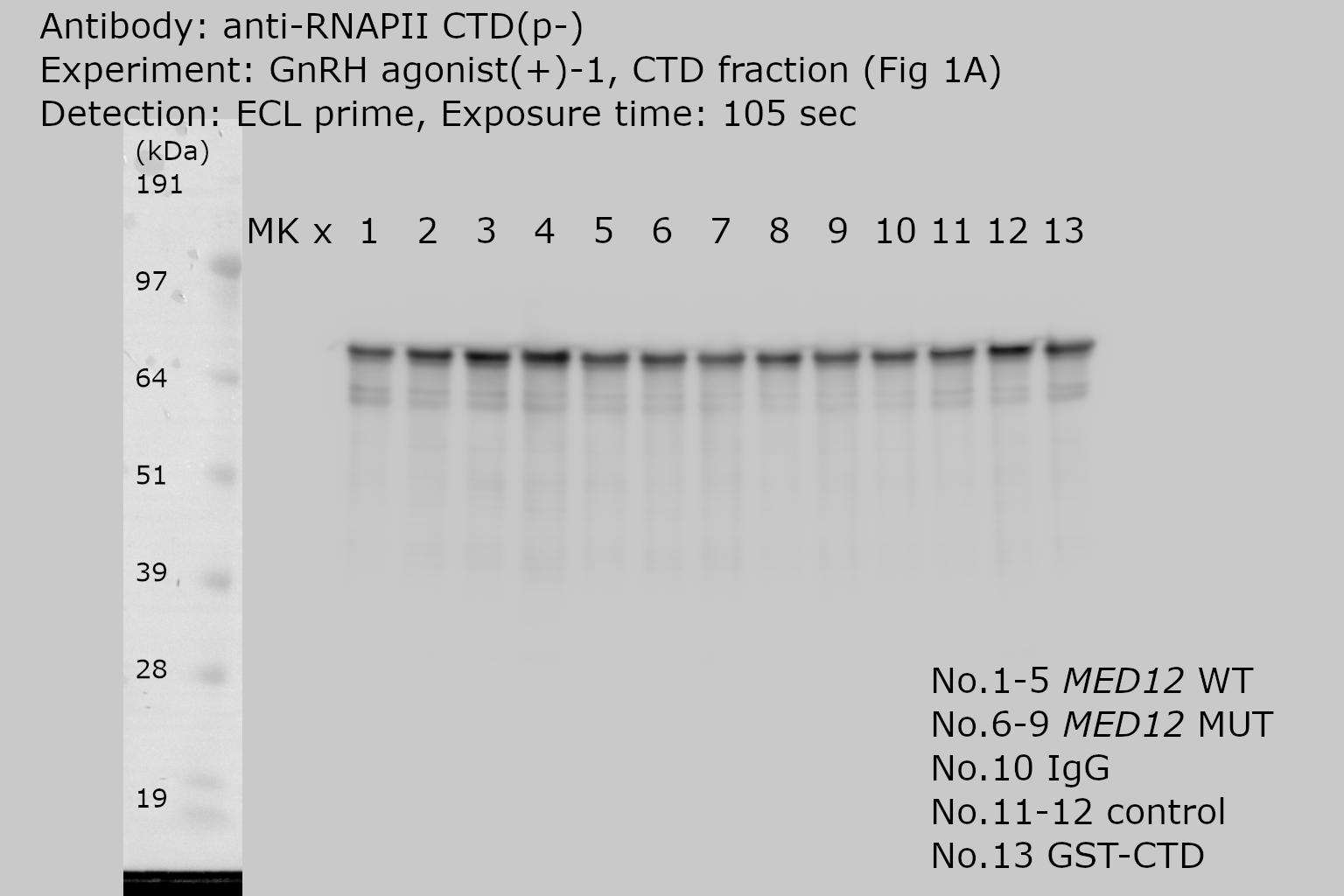

Supplement: S1 Raw Images — (ZIP) [file pone.0338485.s005.zip › CTD-p(-) GnRH+1.tif]

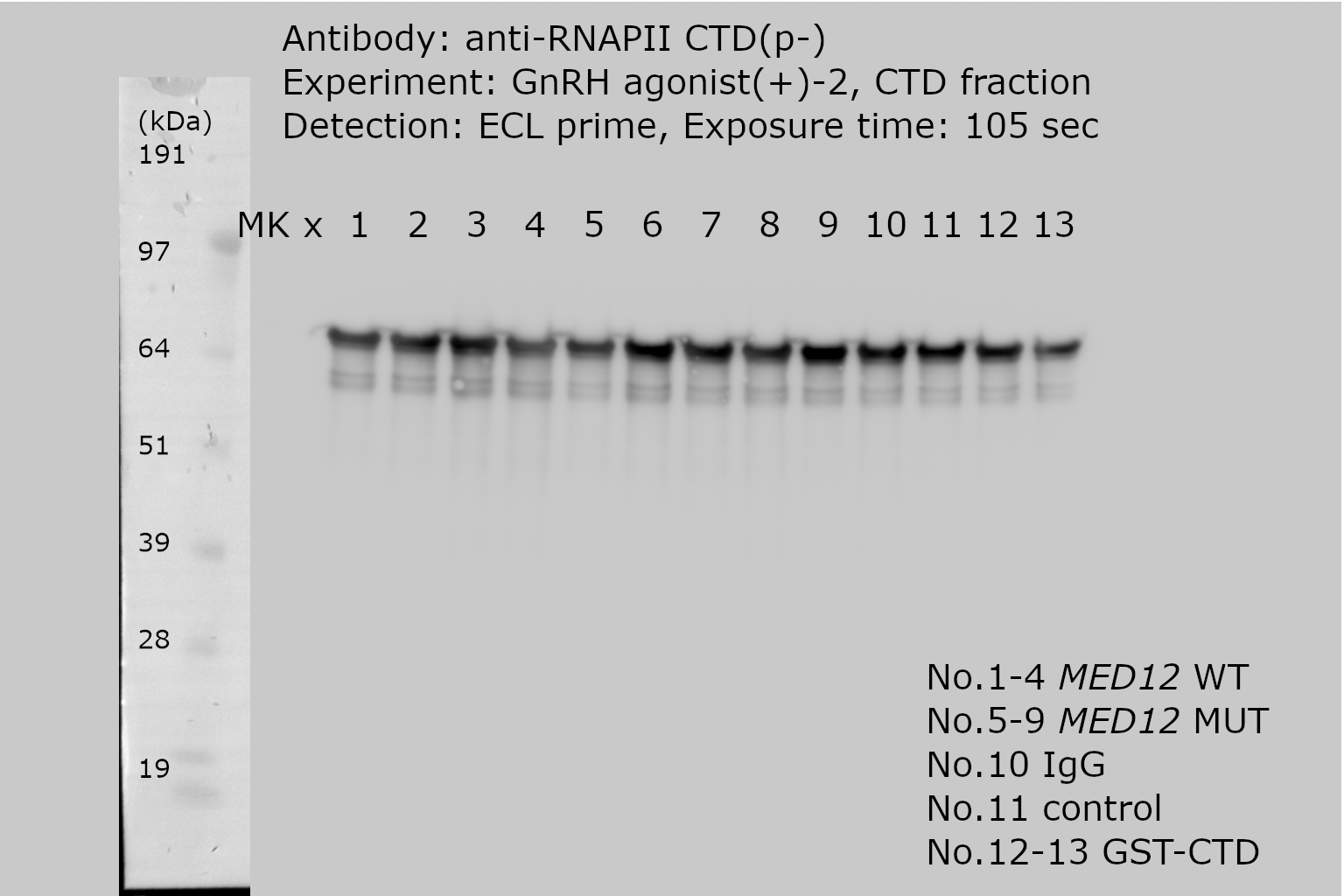

Supplement: S1 Raw Images — (ZIP) [file pone.0338485.s005.zip › CTD-p(-) GnRH+2.tif]

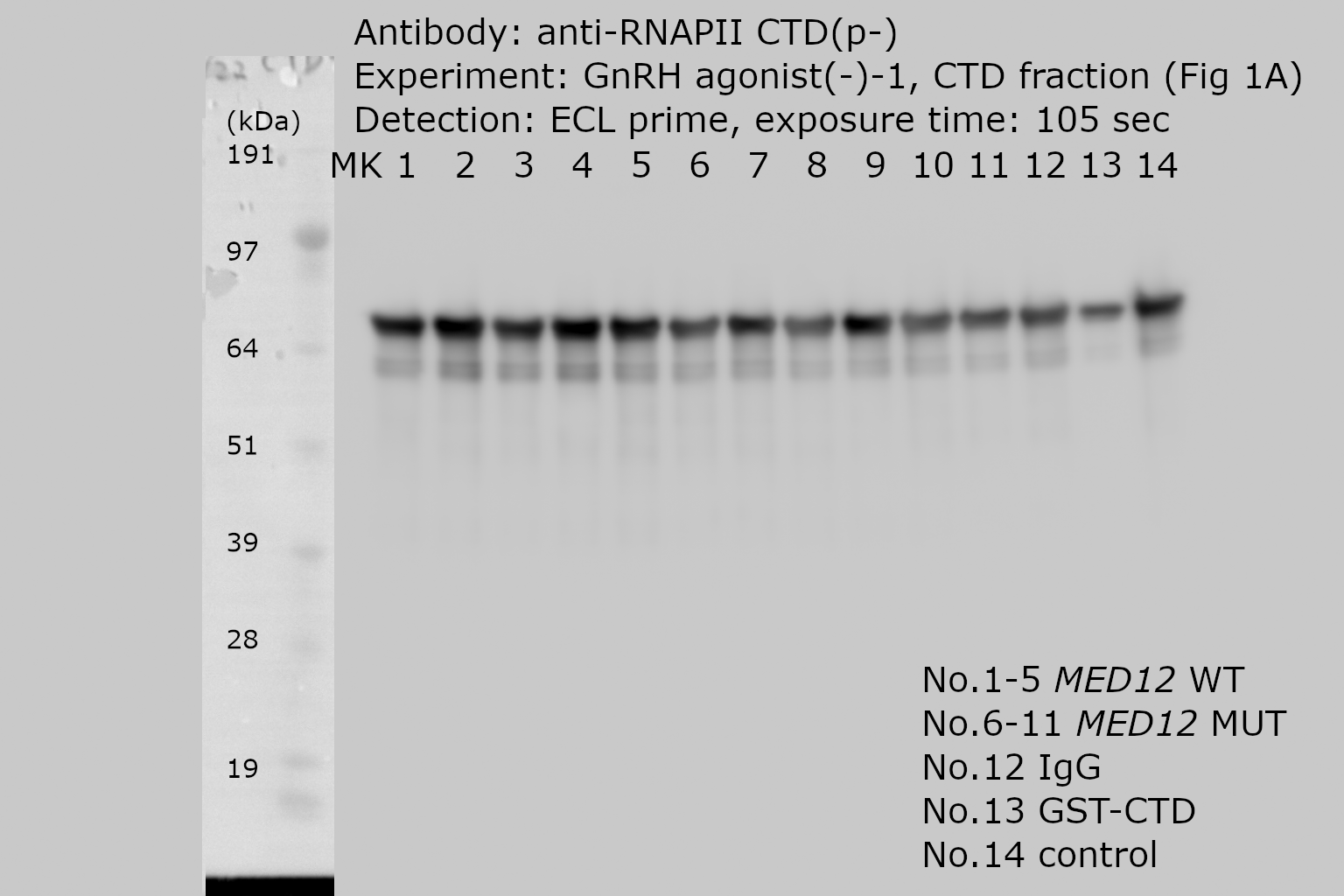

Supplement: S1 Raw Images — (ZIP) [file pone.0338485.s005.zip › CTD-p(-) GnRH-1.tif]

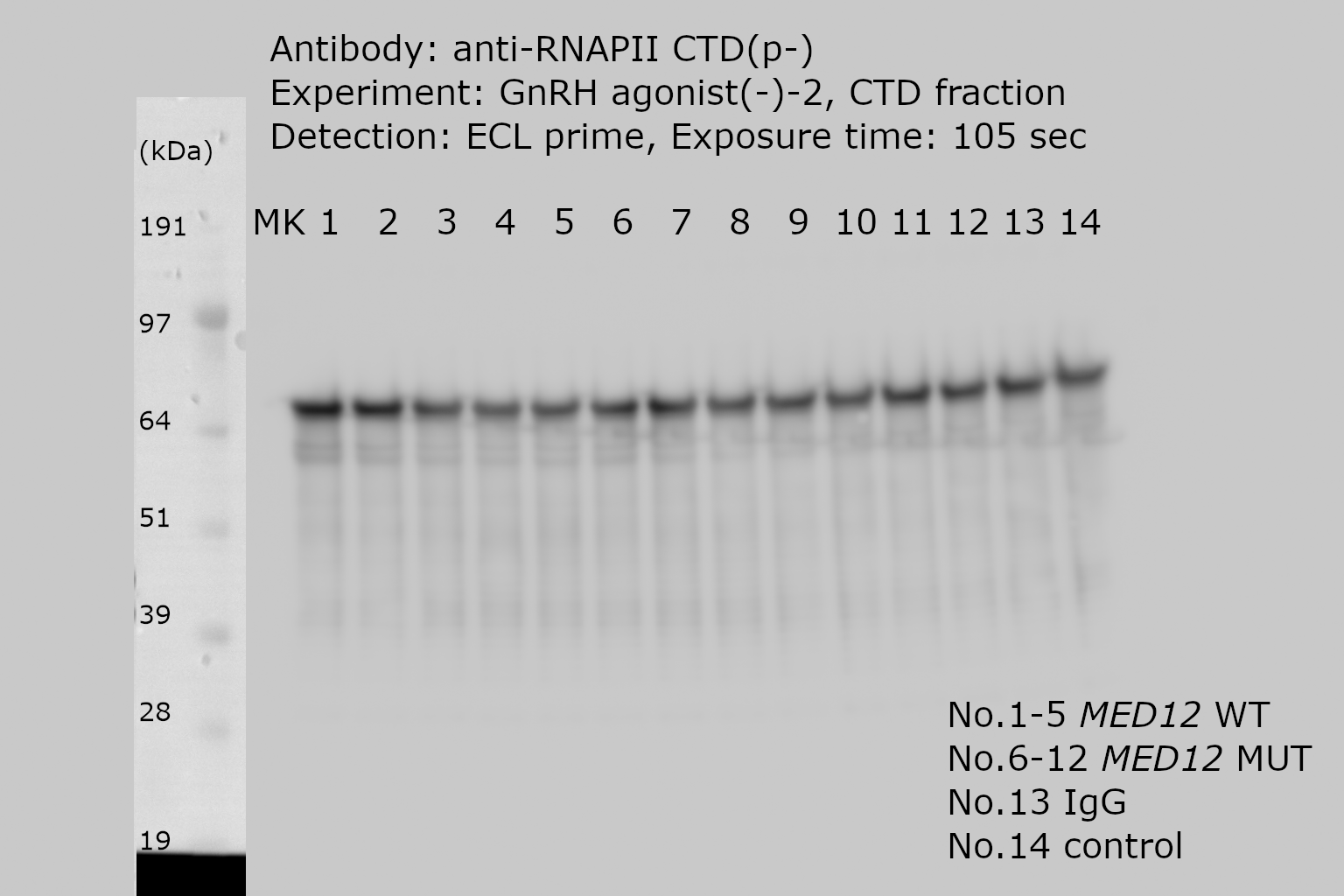

Supplement: S1 Raw Images — (ZIP) [file pone.0338485.s005.zip › CTD-p(-) GnRH-2.tif]

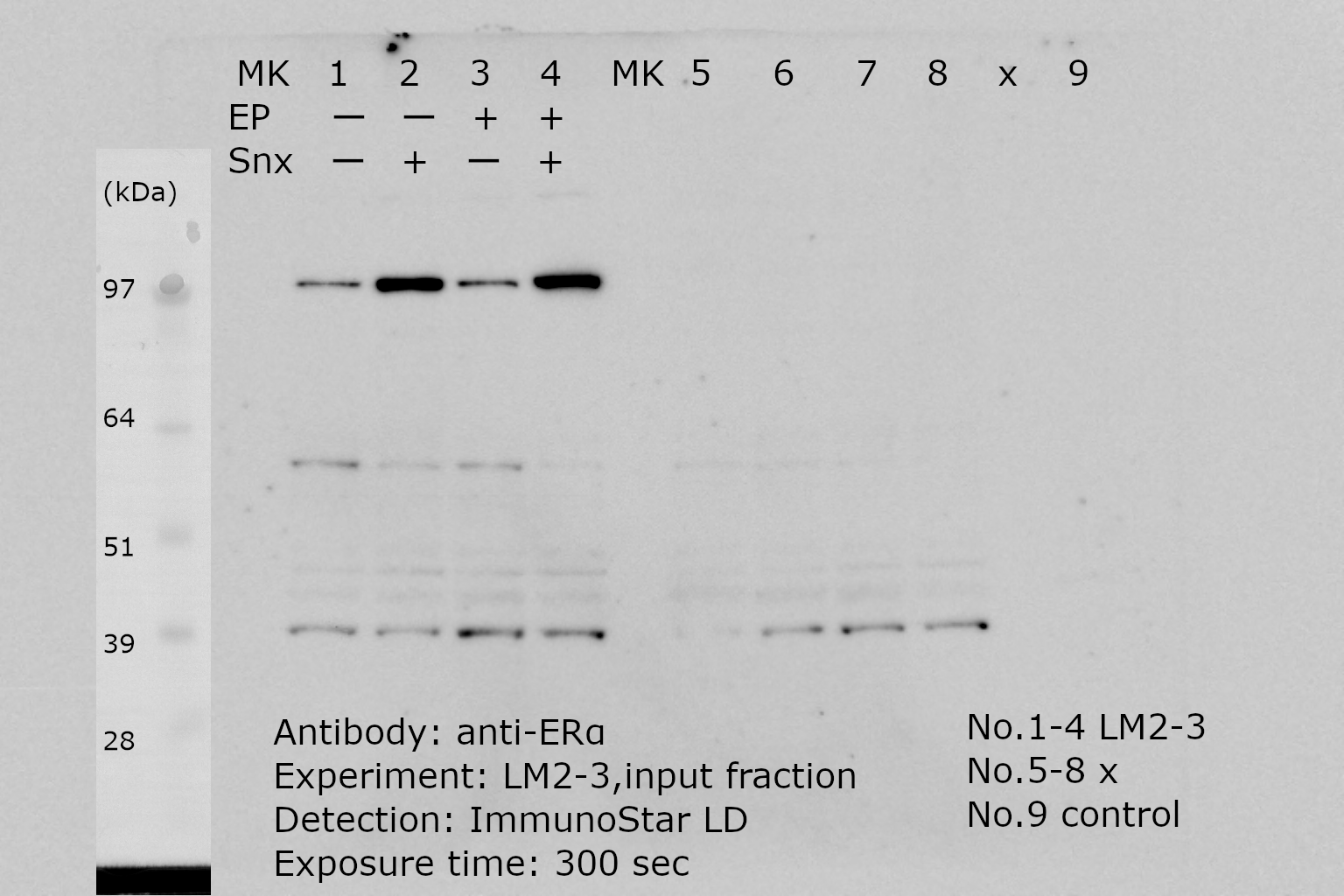

Supplement: S2 Raw Images — (ZIP) [file pone.0338485.s006.zip › LM2-3 ERα.tif]

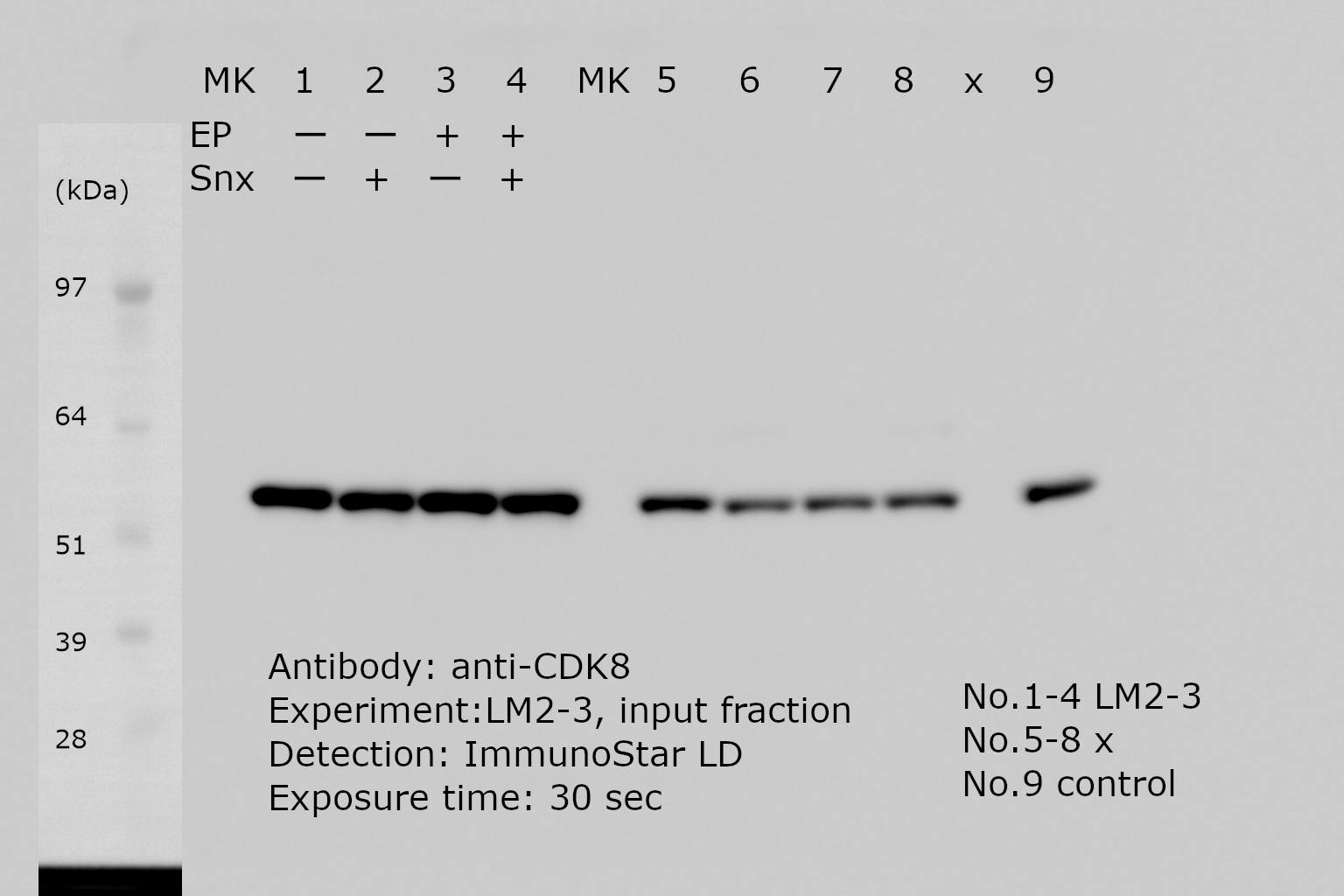

Supplement: S2 Raw Images — (ZIP) [file pone.0338485.s006.zip › LM2-3 input-CDK8.tif]

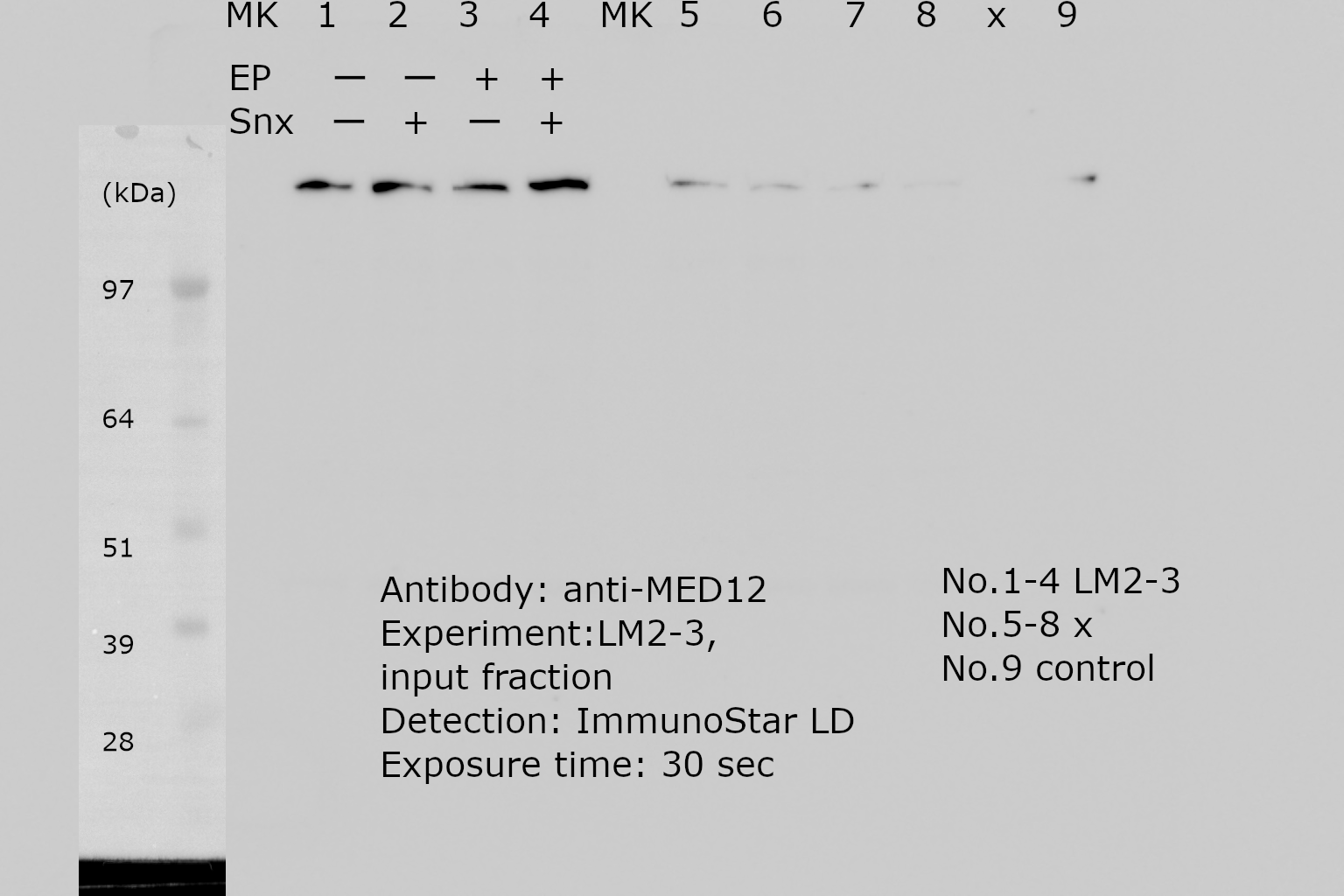

Supplement: S2 Raw Images — (ZIP) [file pone.0338485.s006.zip › LM2-3 input-MED12.tif]

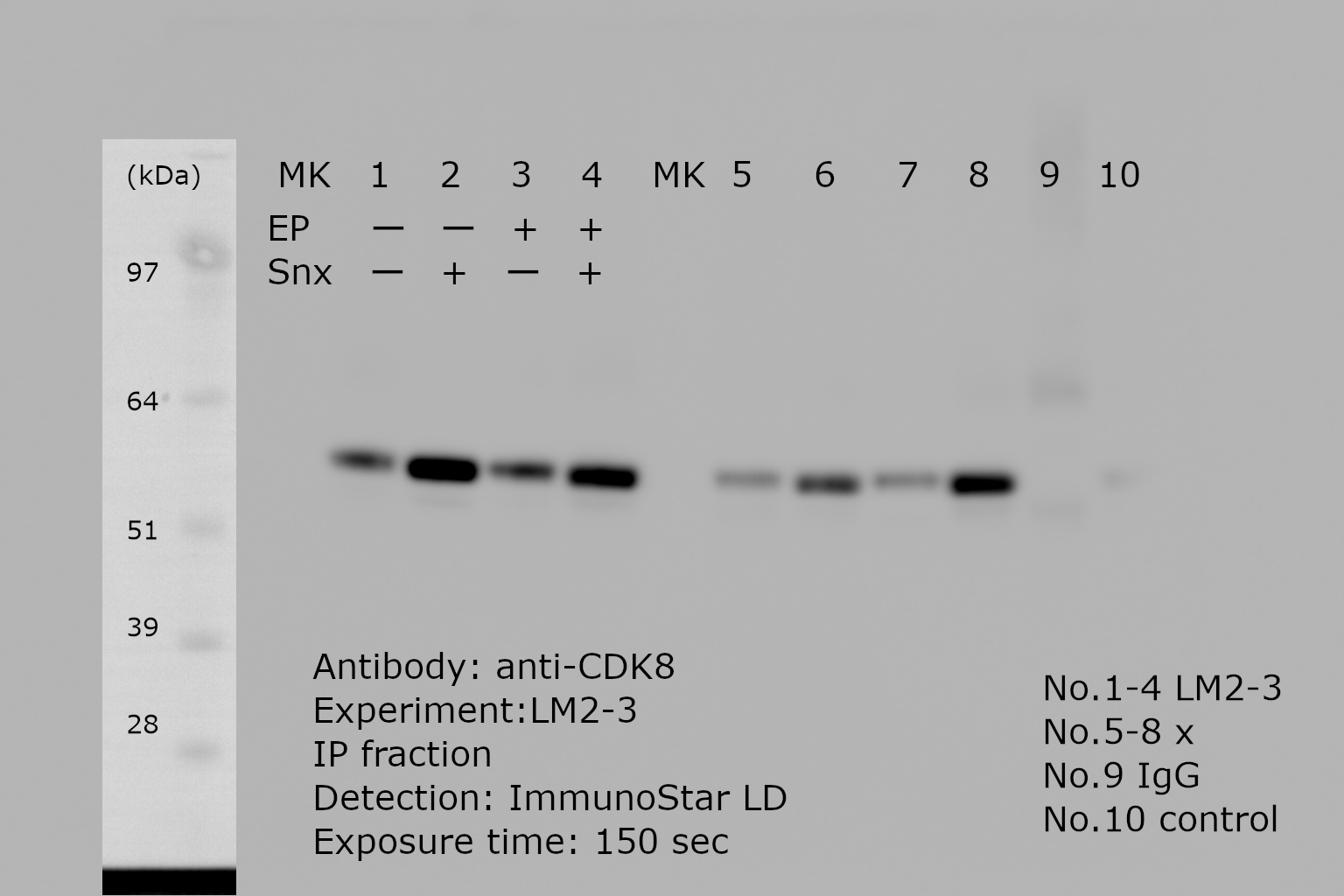

Supplement: S2 Raw Images — (ZIP) [file pone.0338485.s006.zip › LM2-3 IP-CDK8.tif]

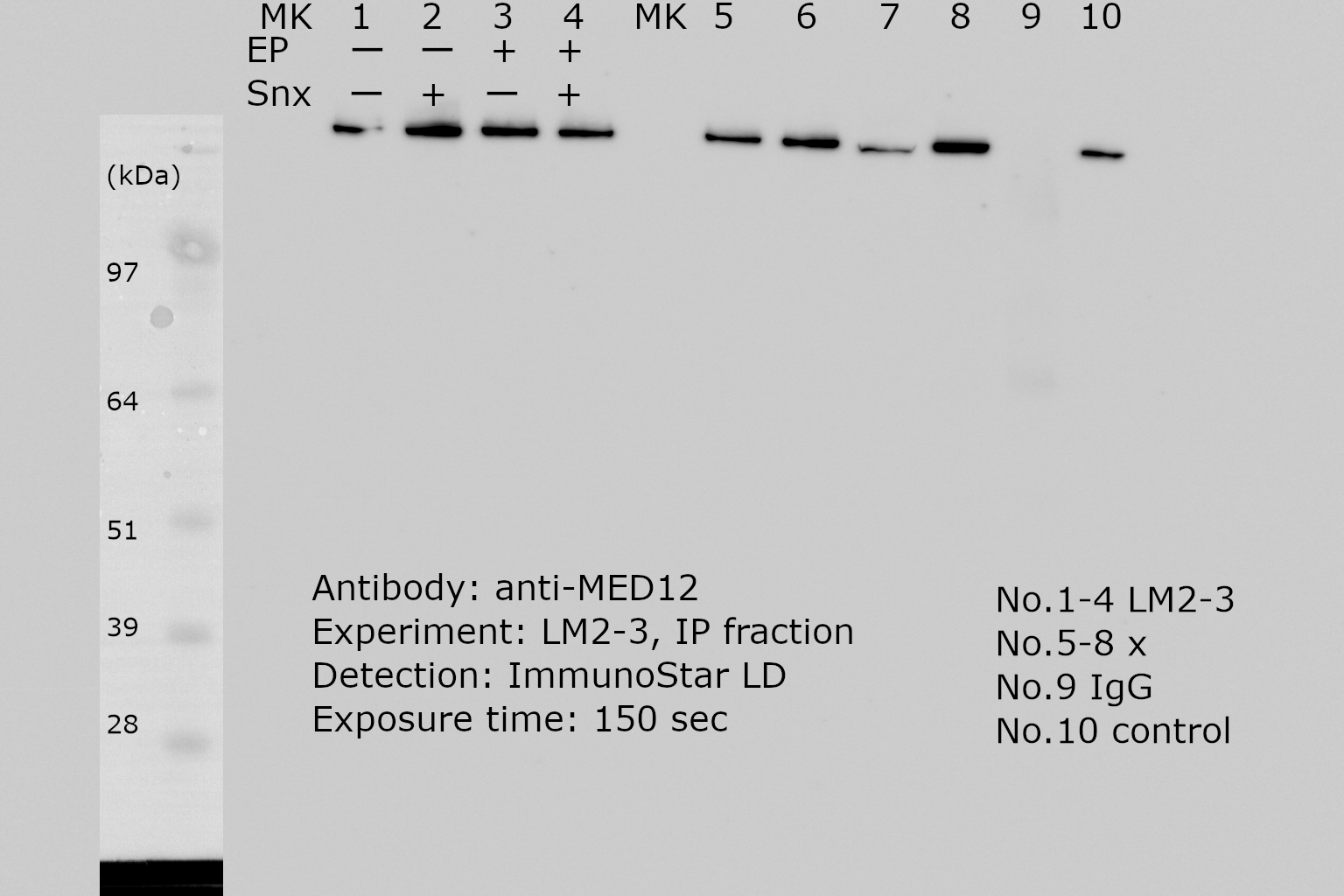

Supplement: S2 Raw Images — (ZIP) [file pone.0338485.s006.zip › LM2-3 IP-MED12.tif]

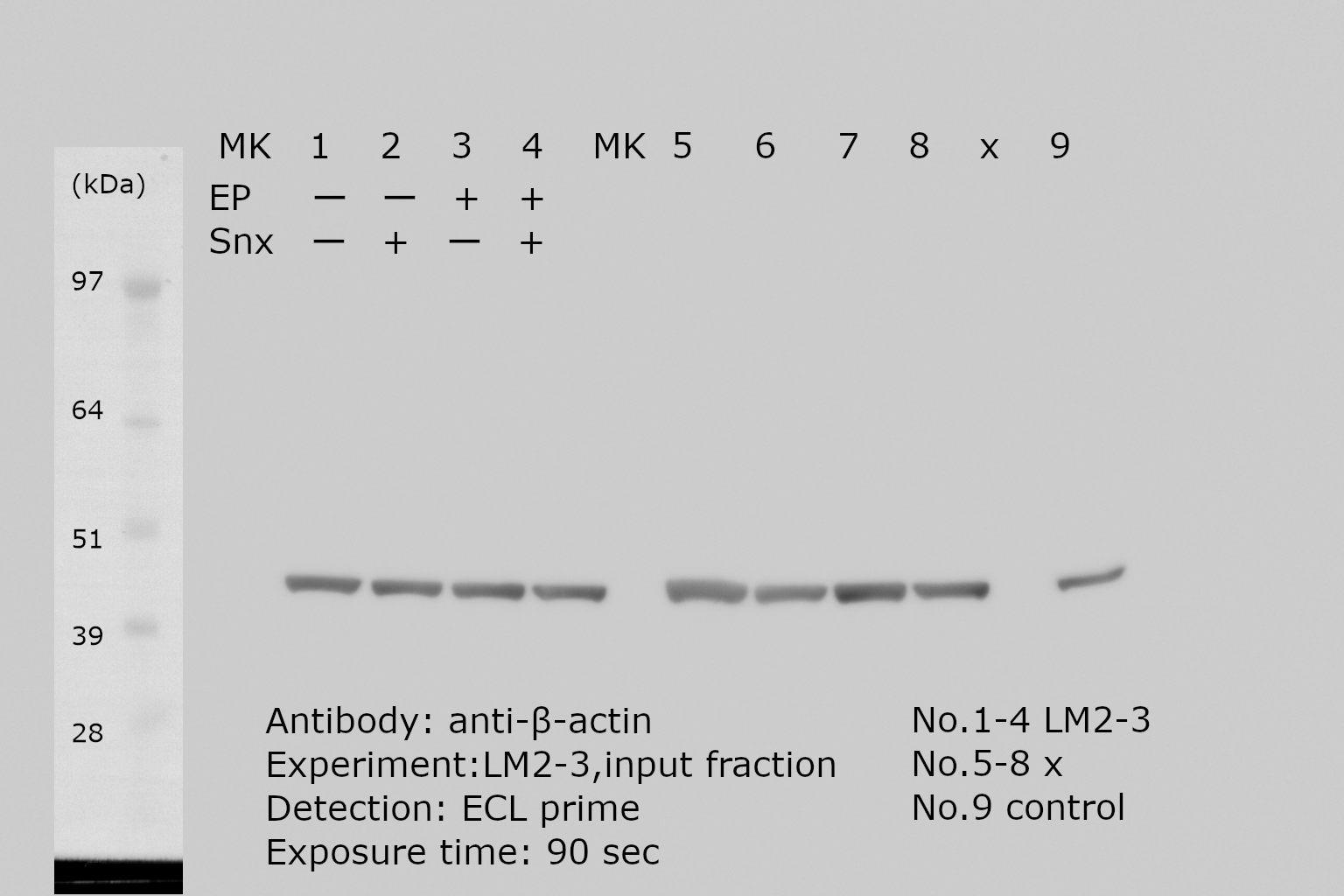

Supplement: S2 Raw Images — (ZIP) [file pone.0338485.s006.zip › LM2-3 βactin.tif]

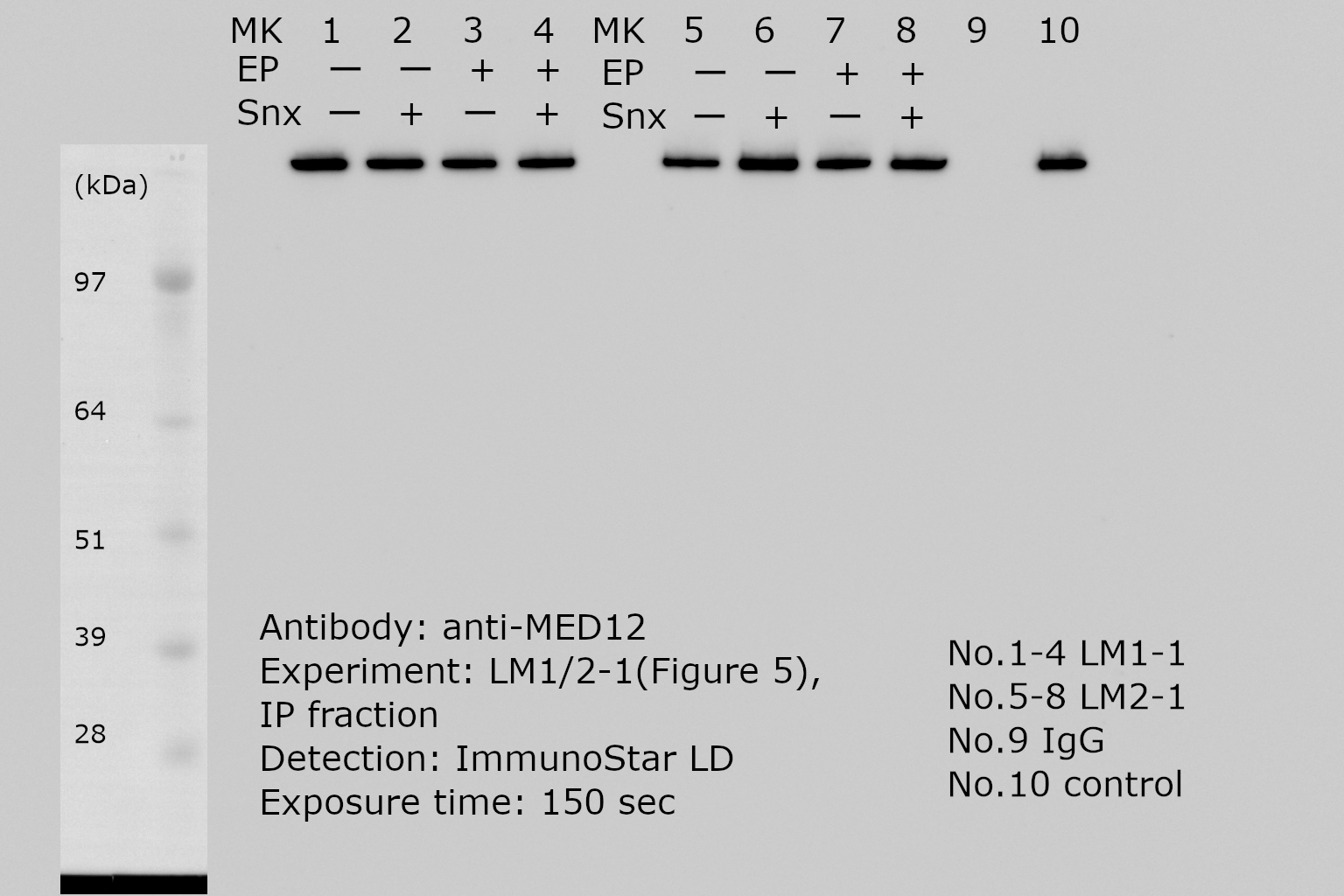

Supplement: S2 Raw Images — (ZIP) [file pone.0338485.s006.zip › LM1,2-1 IP-MED12.tif]

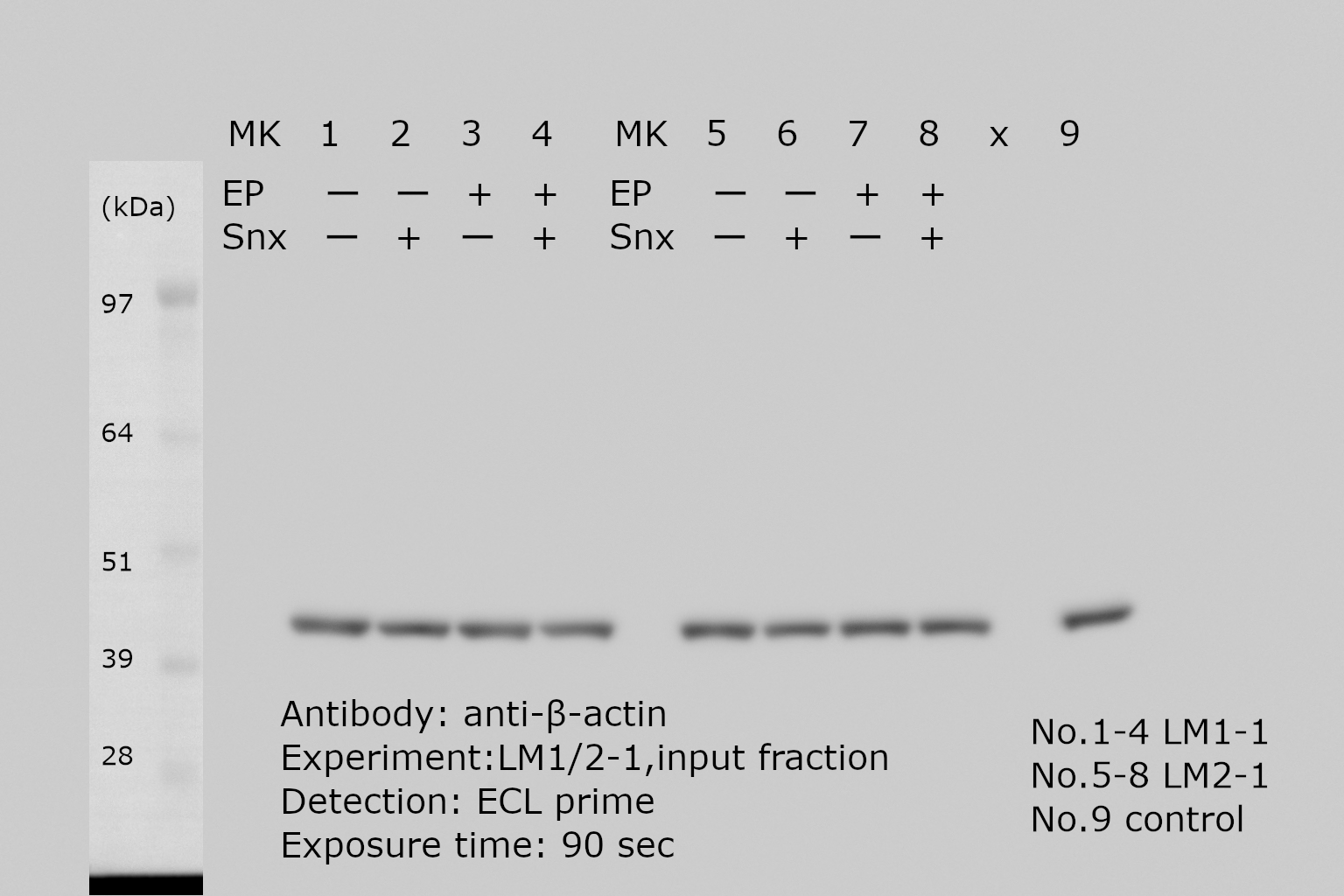

Supplement: S2 Raw Images — (ZIP) [file pone.0338485.s006.zip › LM1,2-1 βactin.tif]

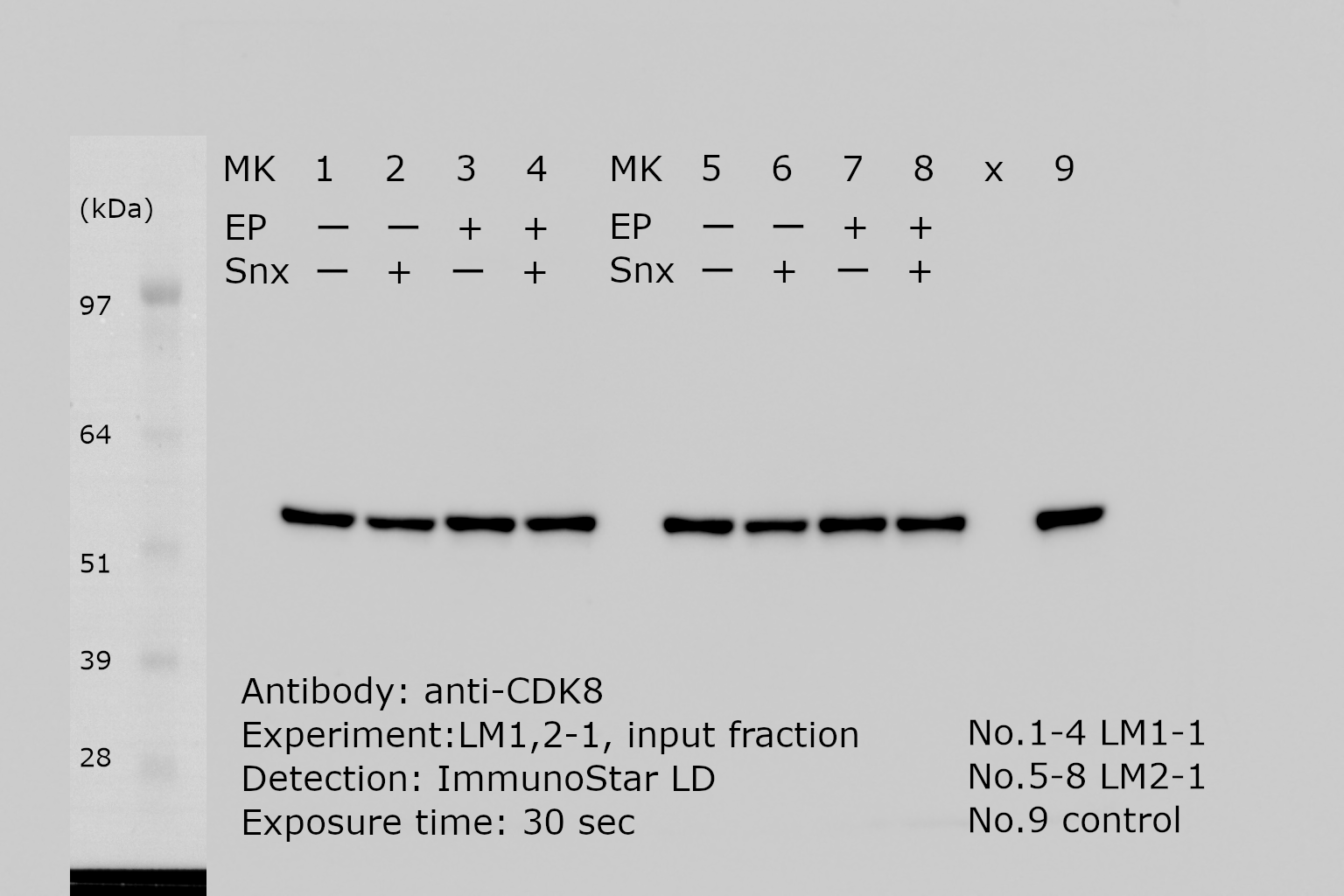

Supplement: S2 Raw Images — (ZIP) [file pone.0338485.s006.zip › LM1,2-1input-CDK8.tif]

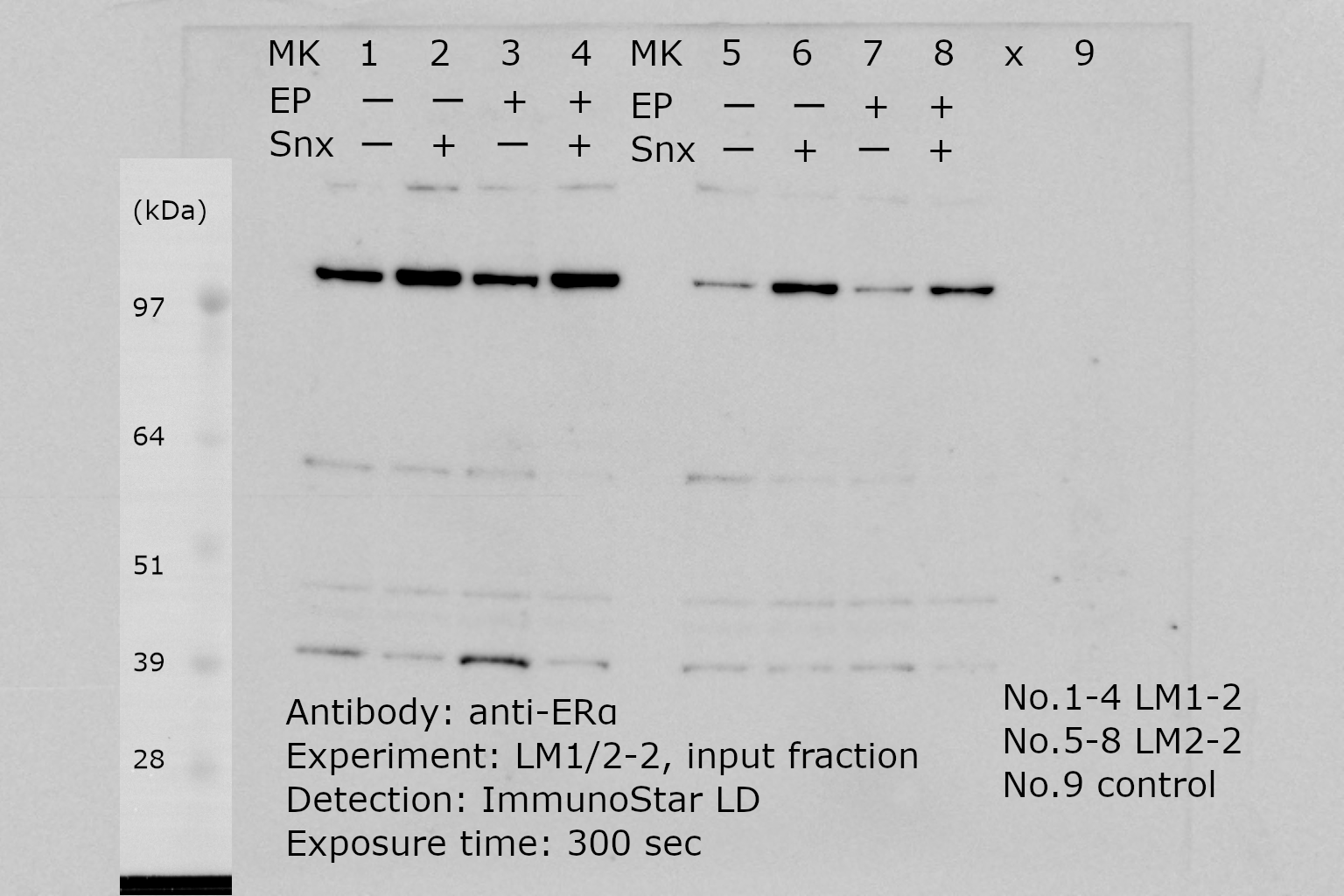

Supplement: S2 Raw Images — (ZIP) [file pone.0338485.s006.zip › LM1,2-2 ERα.tif]

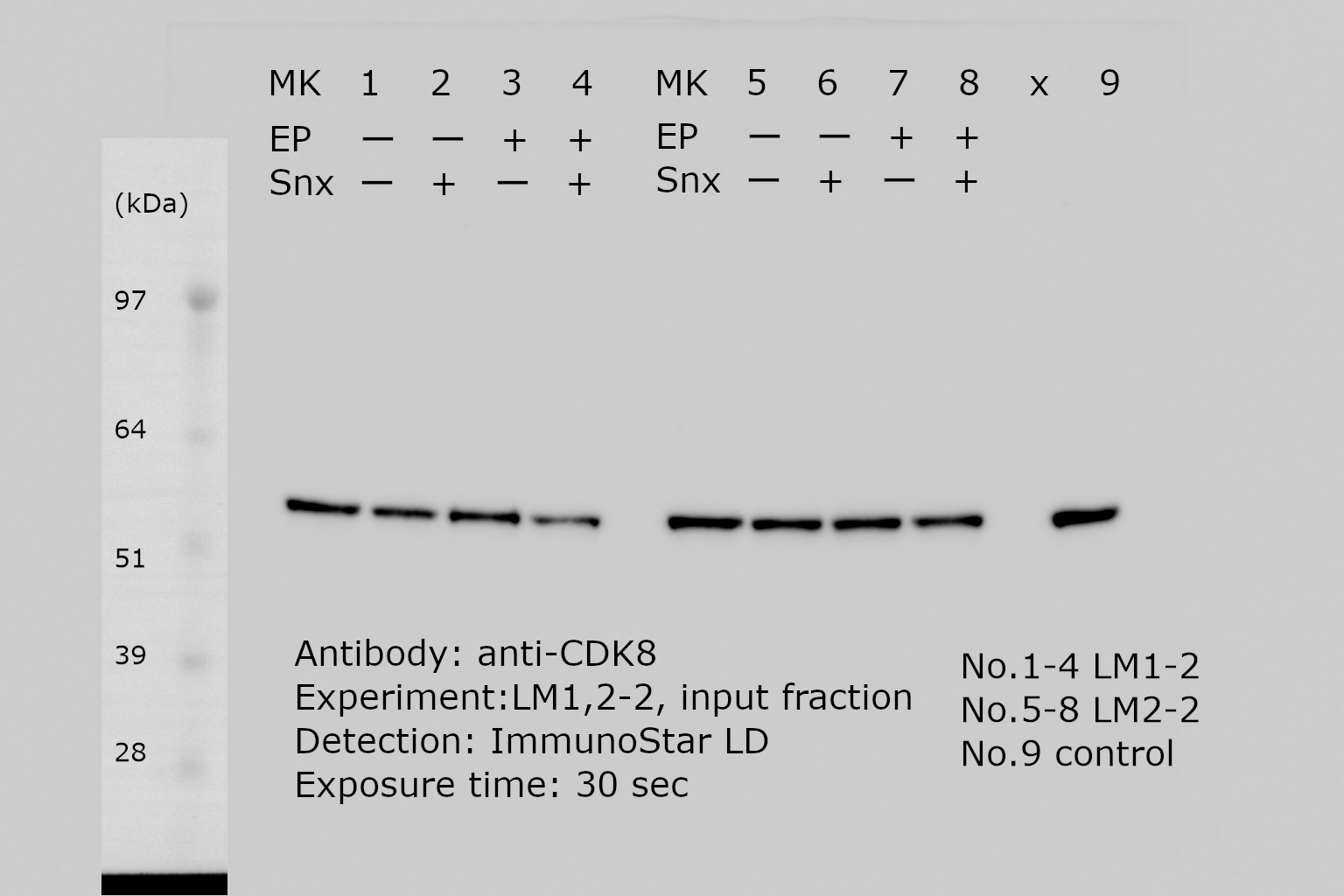

Supplement: S2 Raw Images — (ZIP) [file pone.0338485.s006.zip › LM1,2-2 input-CDK8.tif]

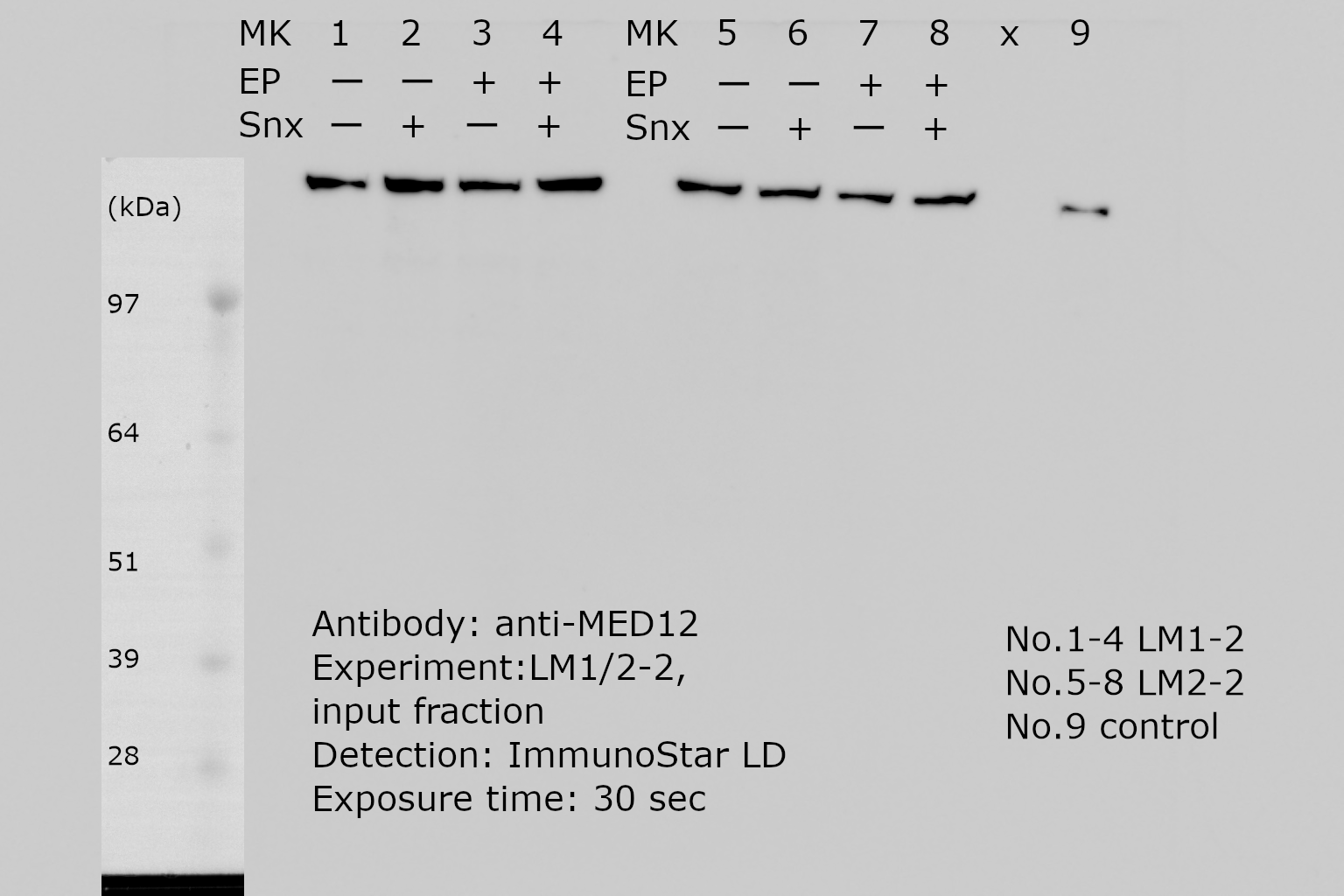

Supplement: S2 Raw Images — (ZIP) [file pone.0338485.s006.zip › LM1,2-2 input-MED12.tif]

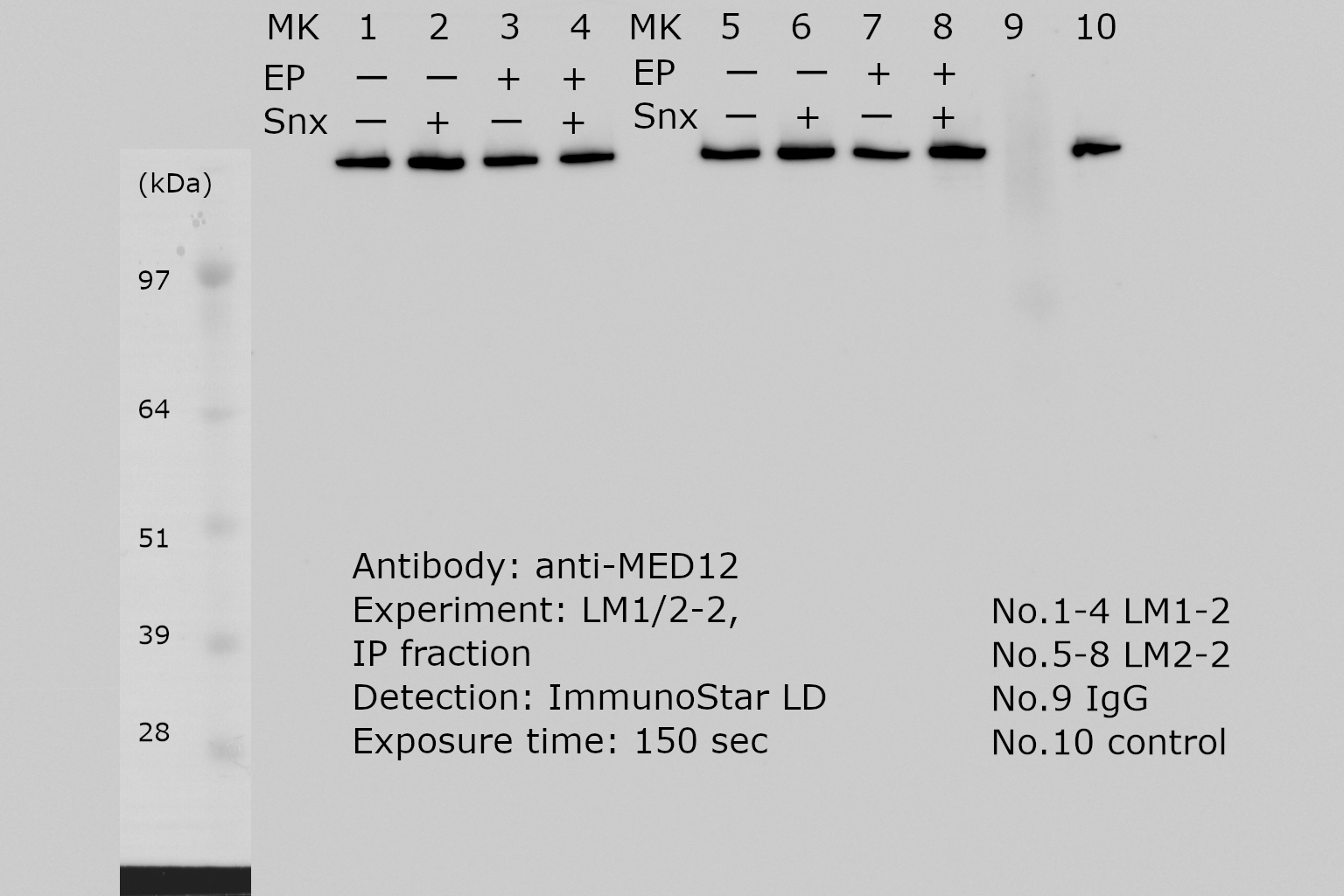

Supplement: S2 Raw Images — (ZIP) [file pone.0338485.s006.zip › LM1,2-2 IP-MED12.tif]

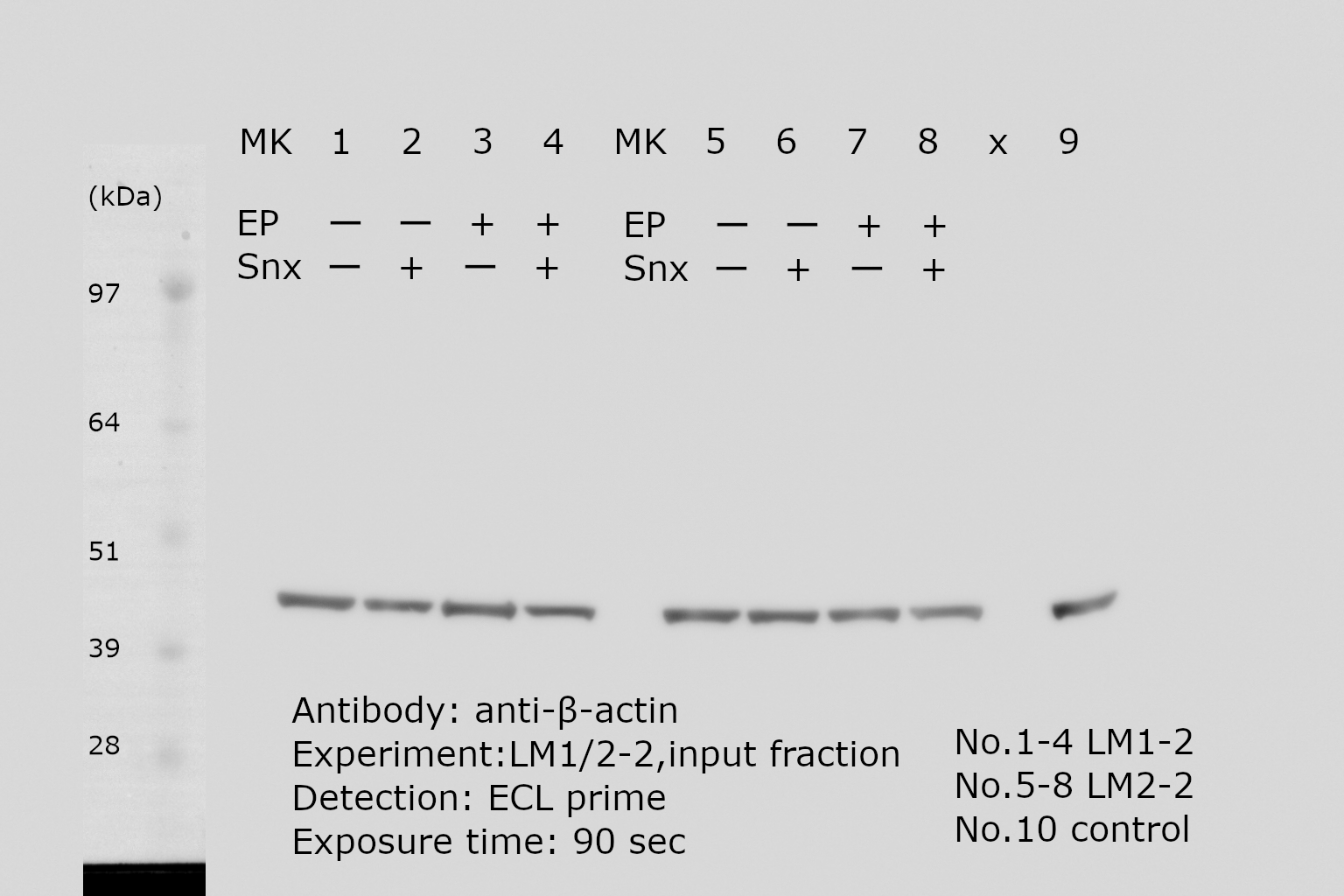

Supplement: S2 Raw Images — (ZIP) [file pone.0338485.s006.zip › LM1,2-2 βactin.tif]

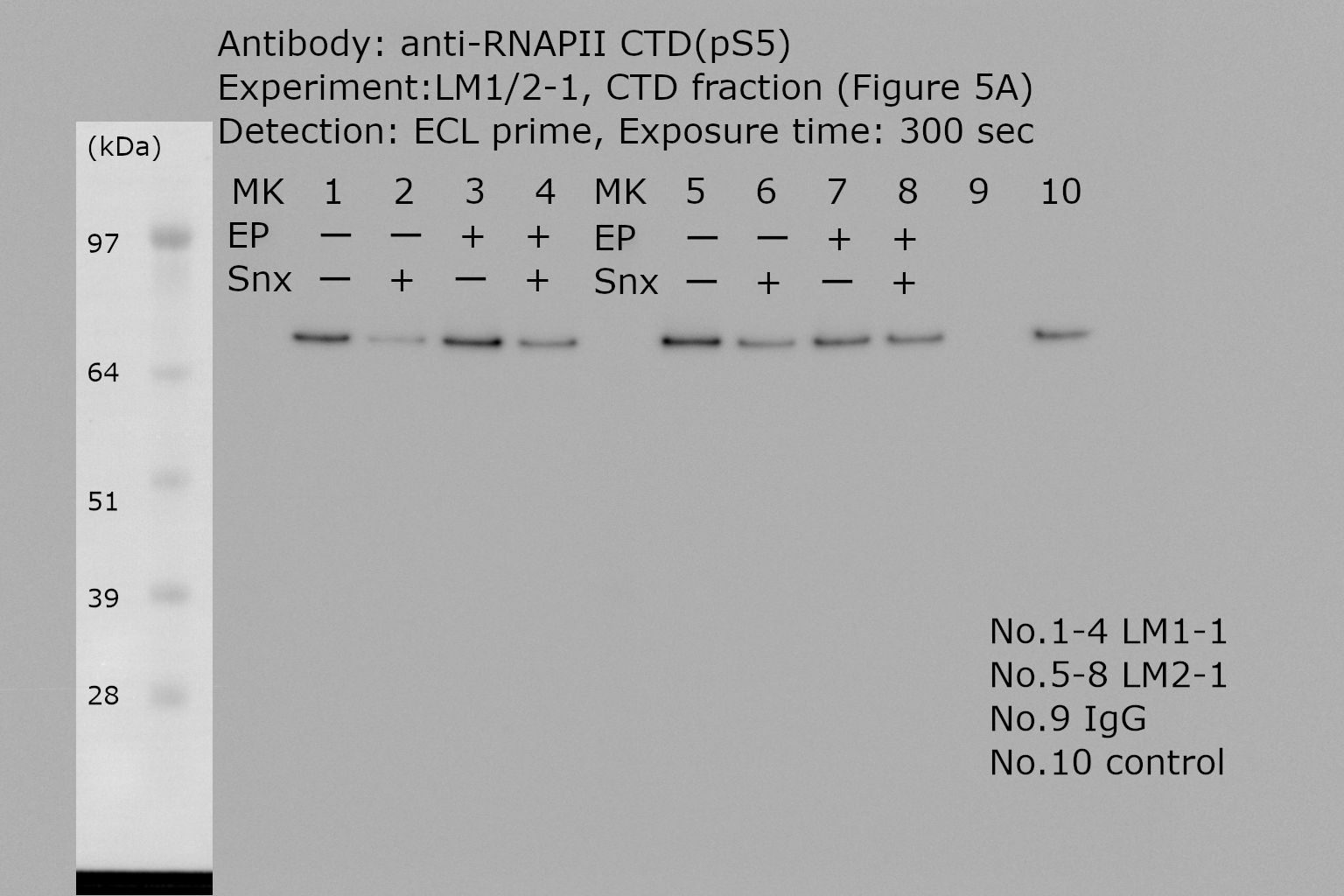

Supplement: S2 Raw Images — (ZIP) [file pone.0338485.s006.zip › LM1.2-1 CTD-pS5.tif]

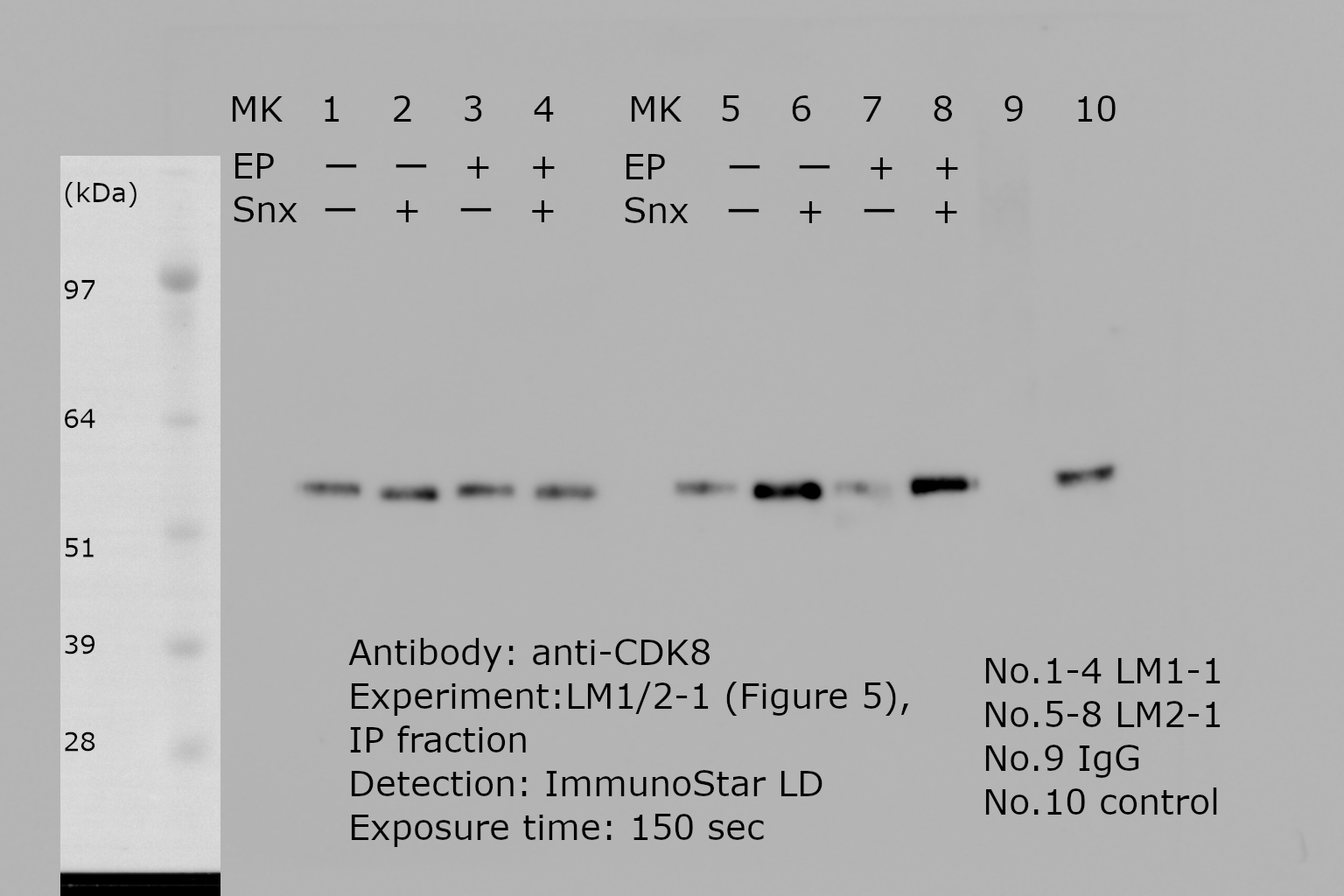

Supplement: S2 Raw Images — (ZIP) [file pone.0338485.s006.zip › LM1.2-1 IP-CDK8.tif]

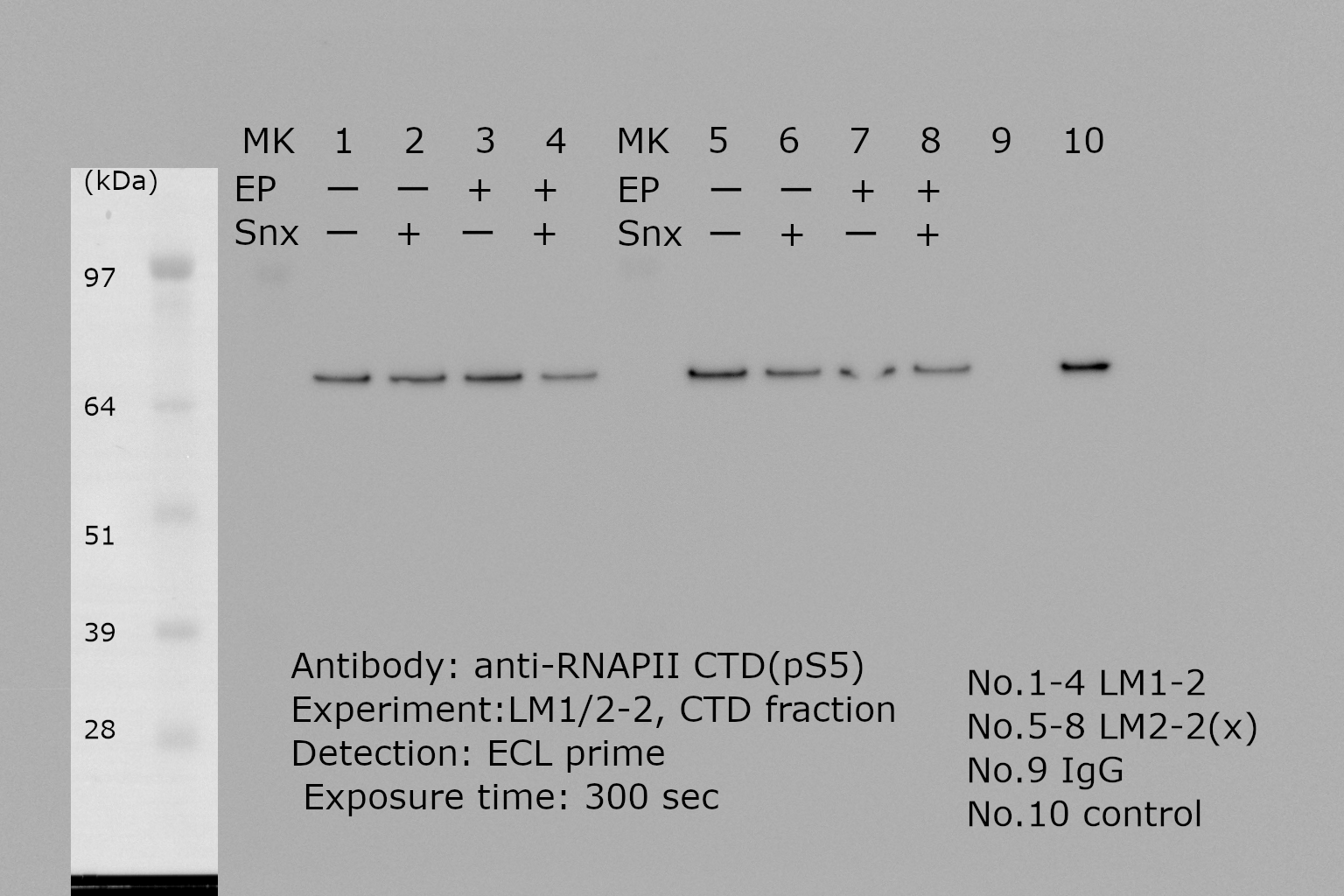

Supplement: S2 Raw Images — (ZIP) [file pone.0338485.s006.zip › LM1.2-2 CTD-pS5.tif]

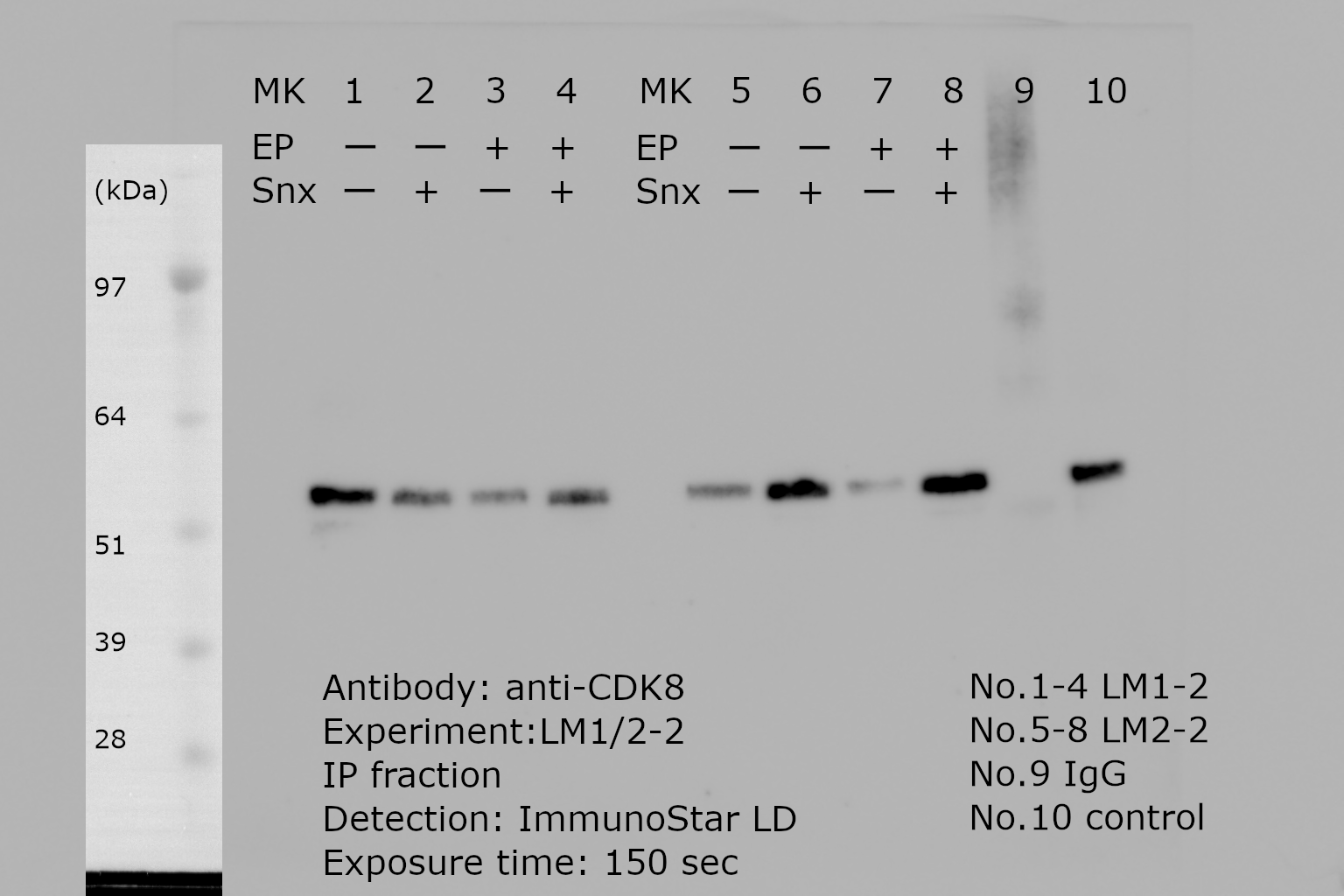

Supplement: S2 Raw Images — (ZIP) [file pone.0338485.s006.zip › LM1.2-2 IP-CDK8.tif]

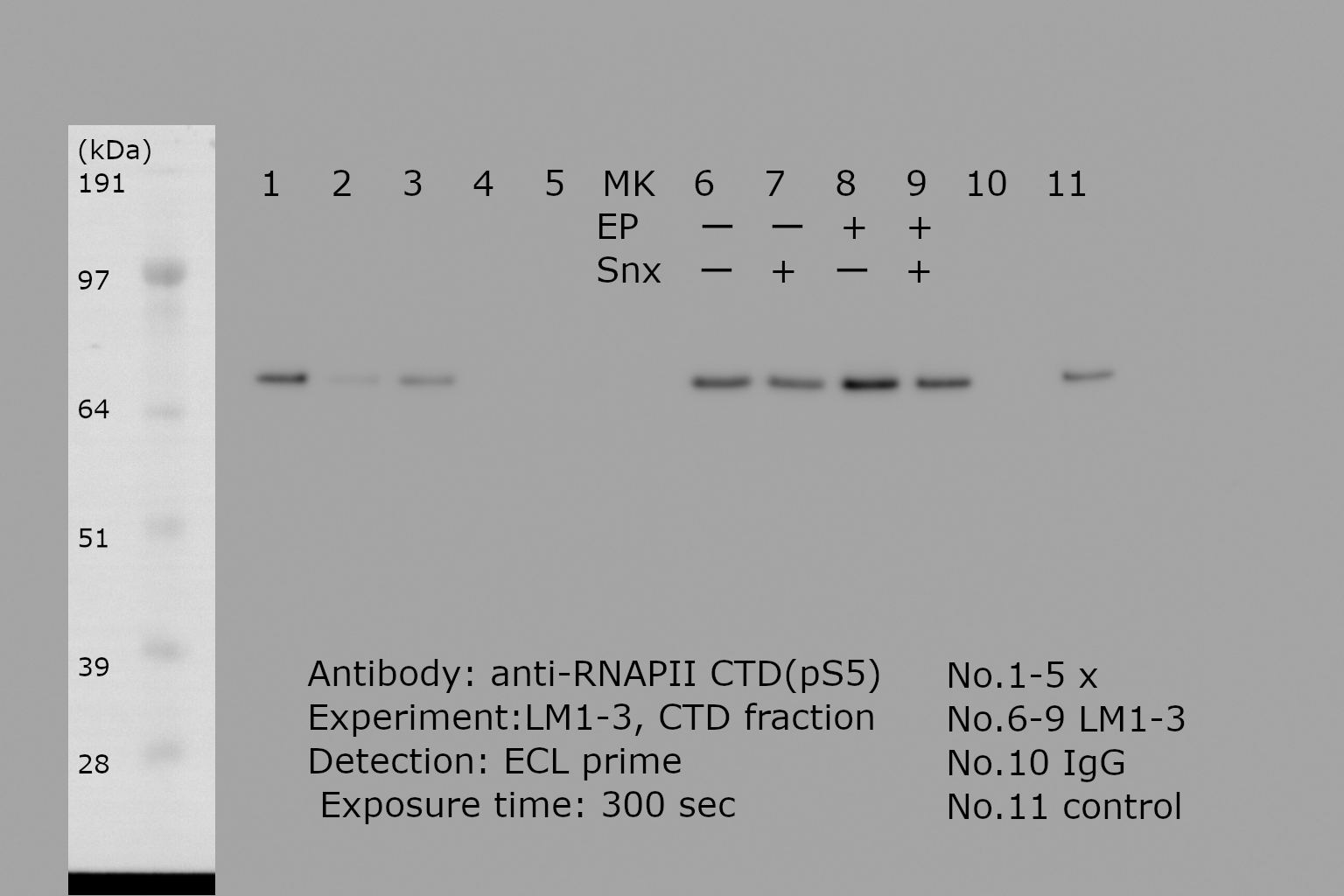

Supplement: S2 Raw Images — (ZIP) [file pone.0338485.s006.zip › LM1-3 CTD-pS5.tif]

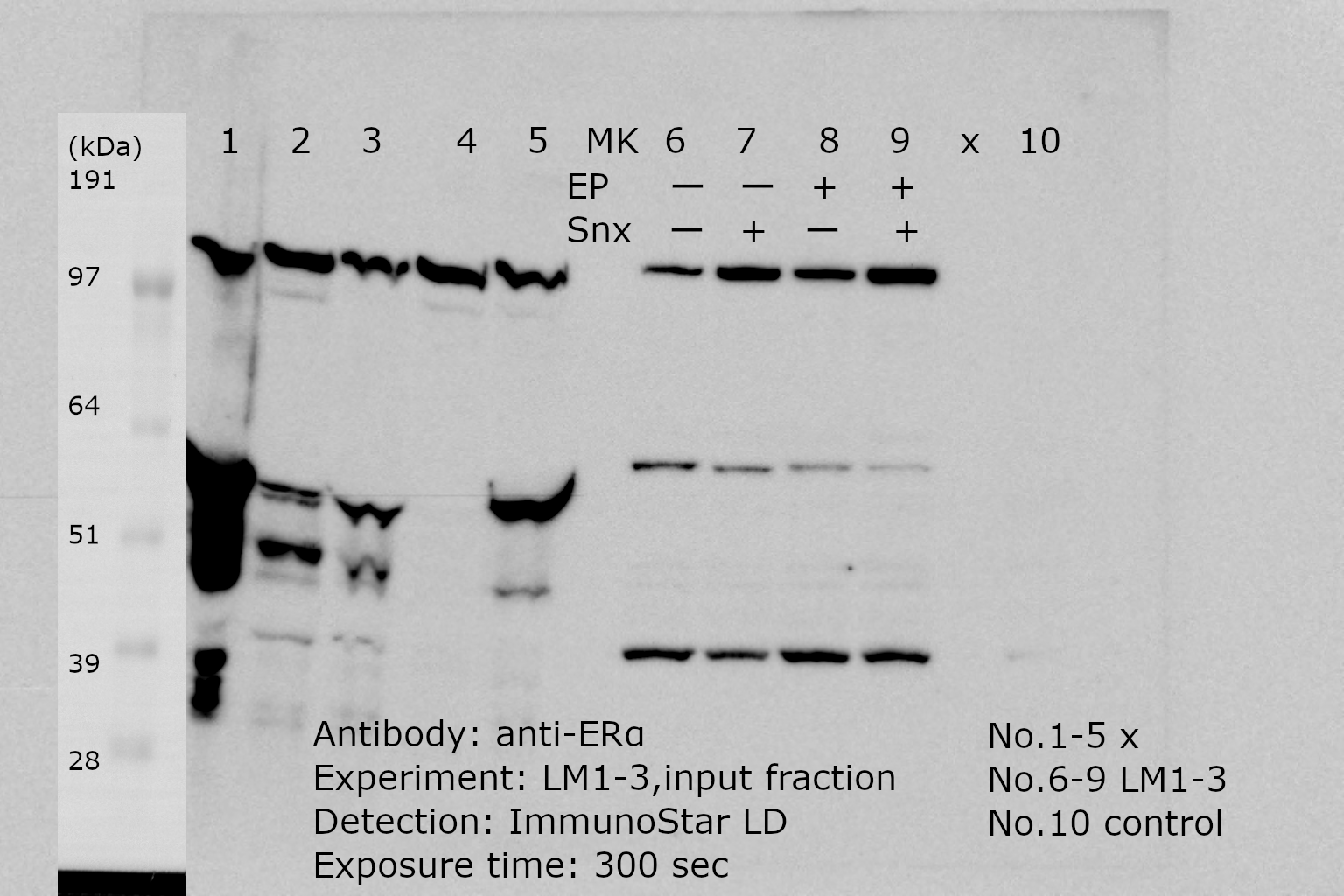

Supplement: S2 Raw Images — (ZIP) [file pone.0338485.s006.zip › LM1-3 ERα.tif]

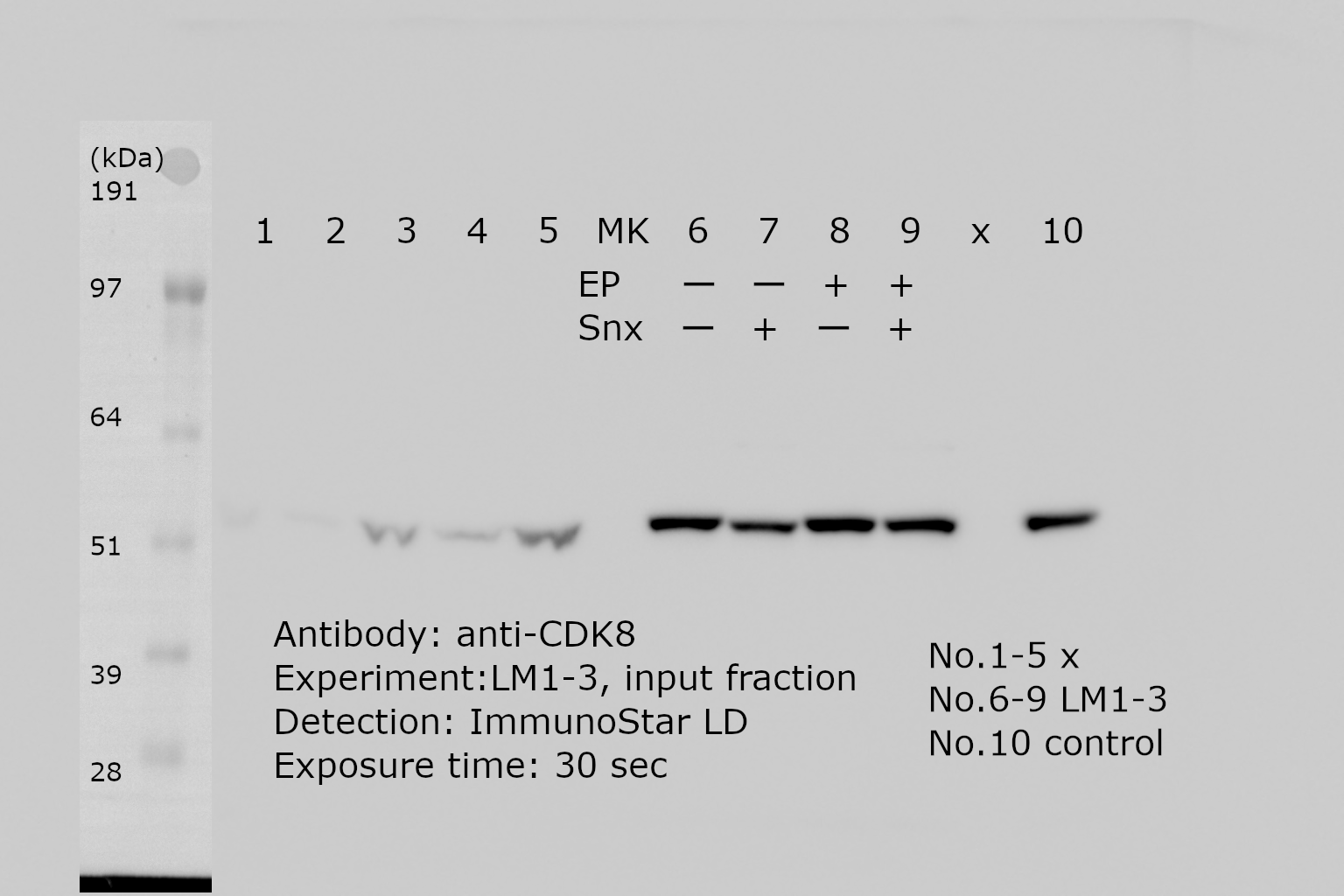

Supplement: S2 Raw Images — (ZIP) [file pone.0338485.s006.zip › LM1-3 input-CDK8.tif]

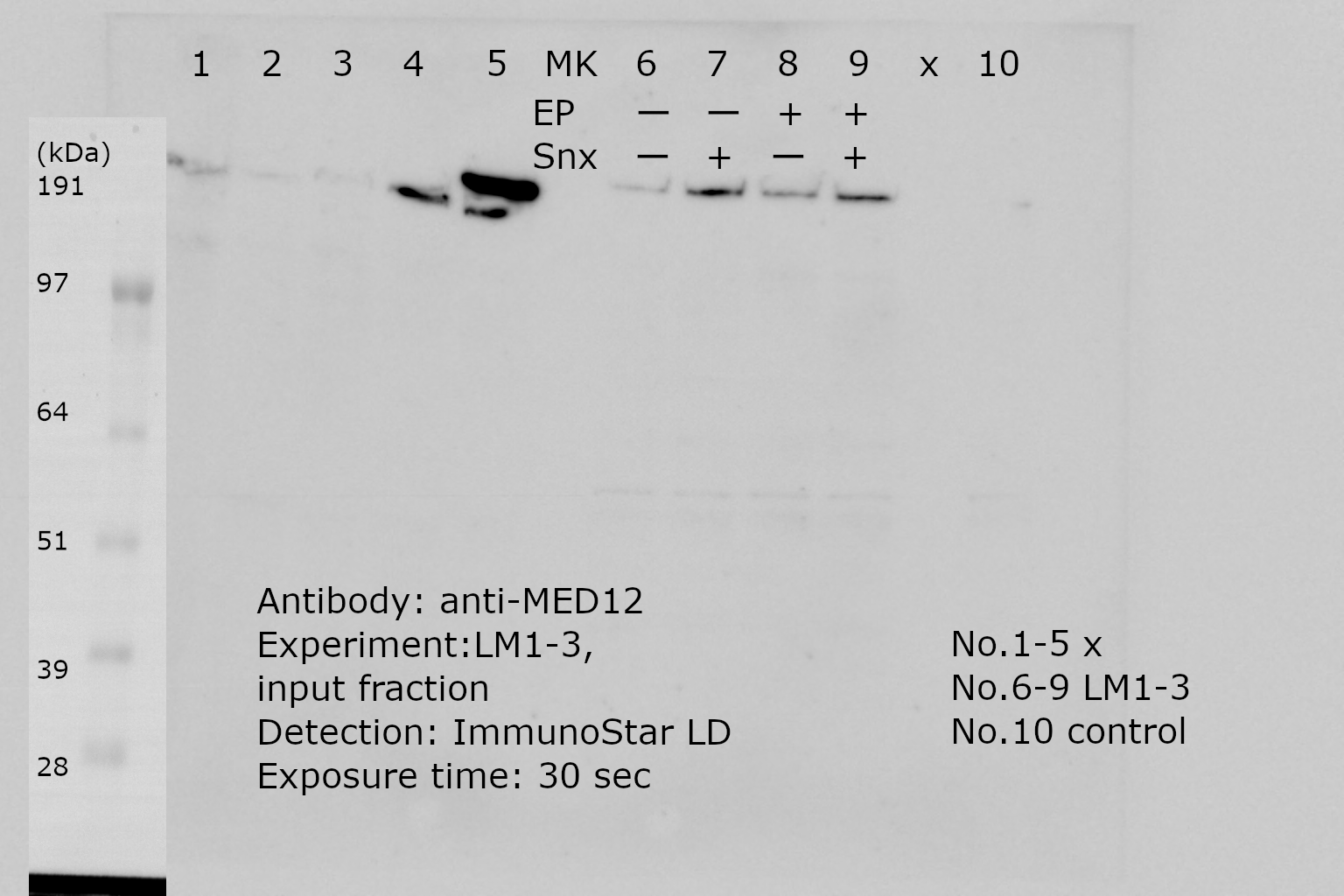

Supplement: S2 Raw Images — (ZIP) [file pone.0338485.s006.zip › LM1-3 input-MED12.tif]

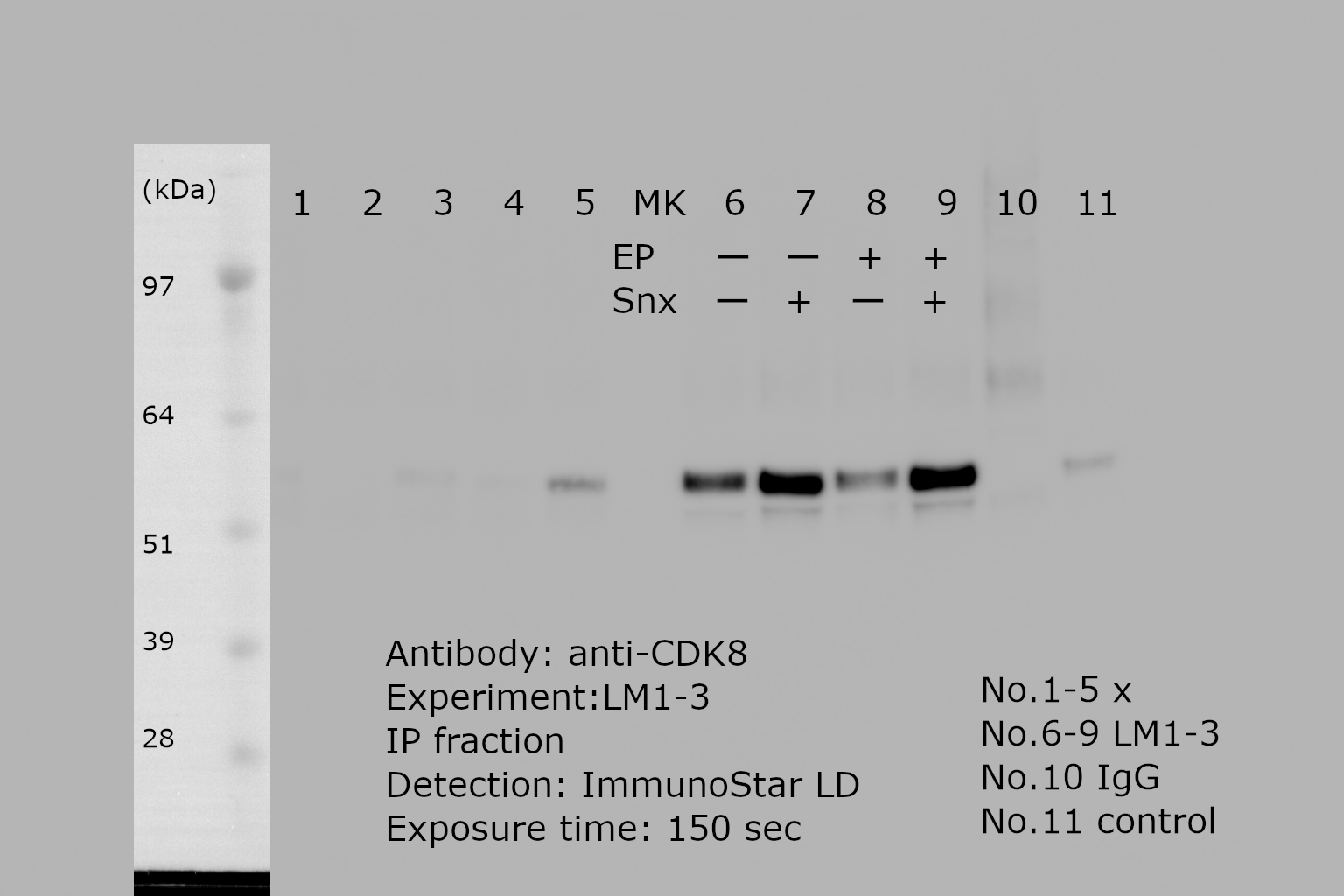

Supplement: S2 Raw Images — (ZIP) [file pone.0338485.s006.zip › LM1-3 IP-CDK8.tif]

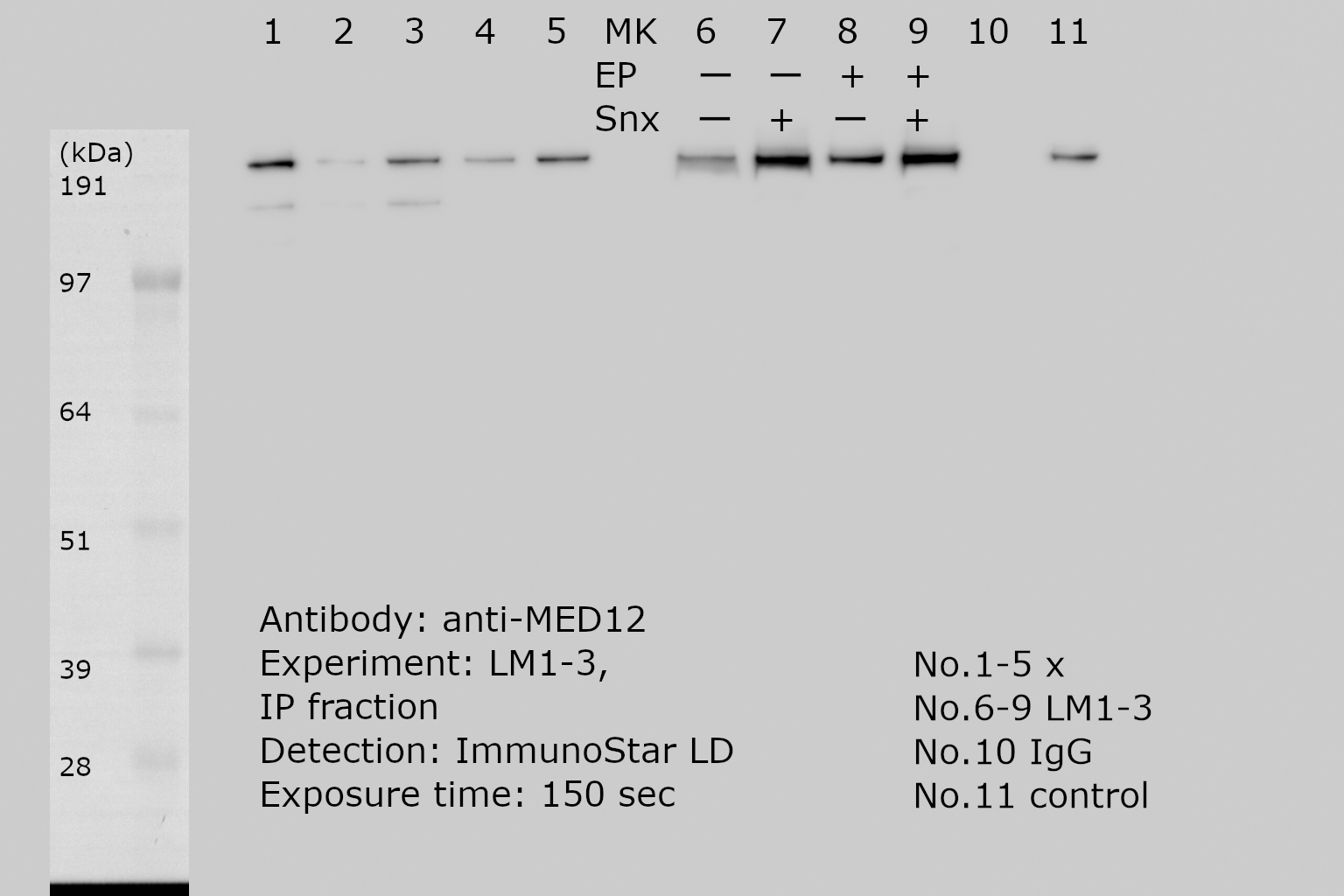

Supplement: S2 Raw Images — (ZIP) [file pone.0338485.s006.zip › LM1-3 IP-MED12.tif]

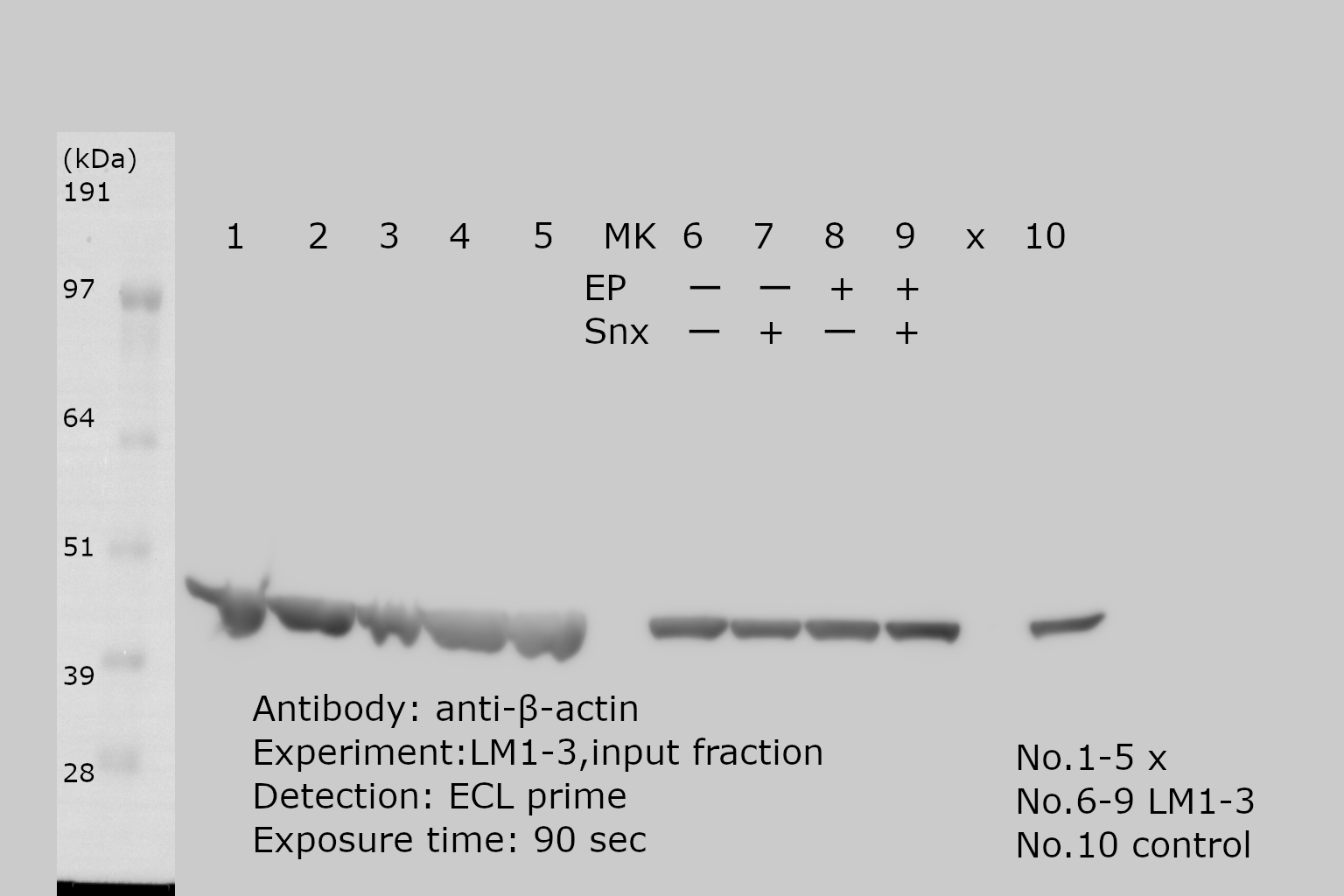

Supplement: S2 Raw Images — (ZIP) [file pone.0338485.s006.zip › LM1-3 βactin.tif]

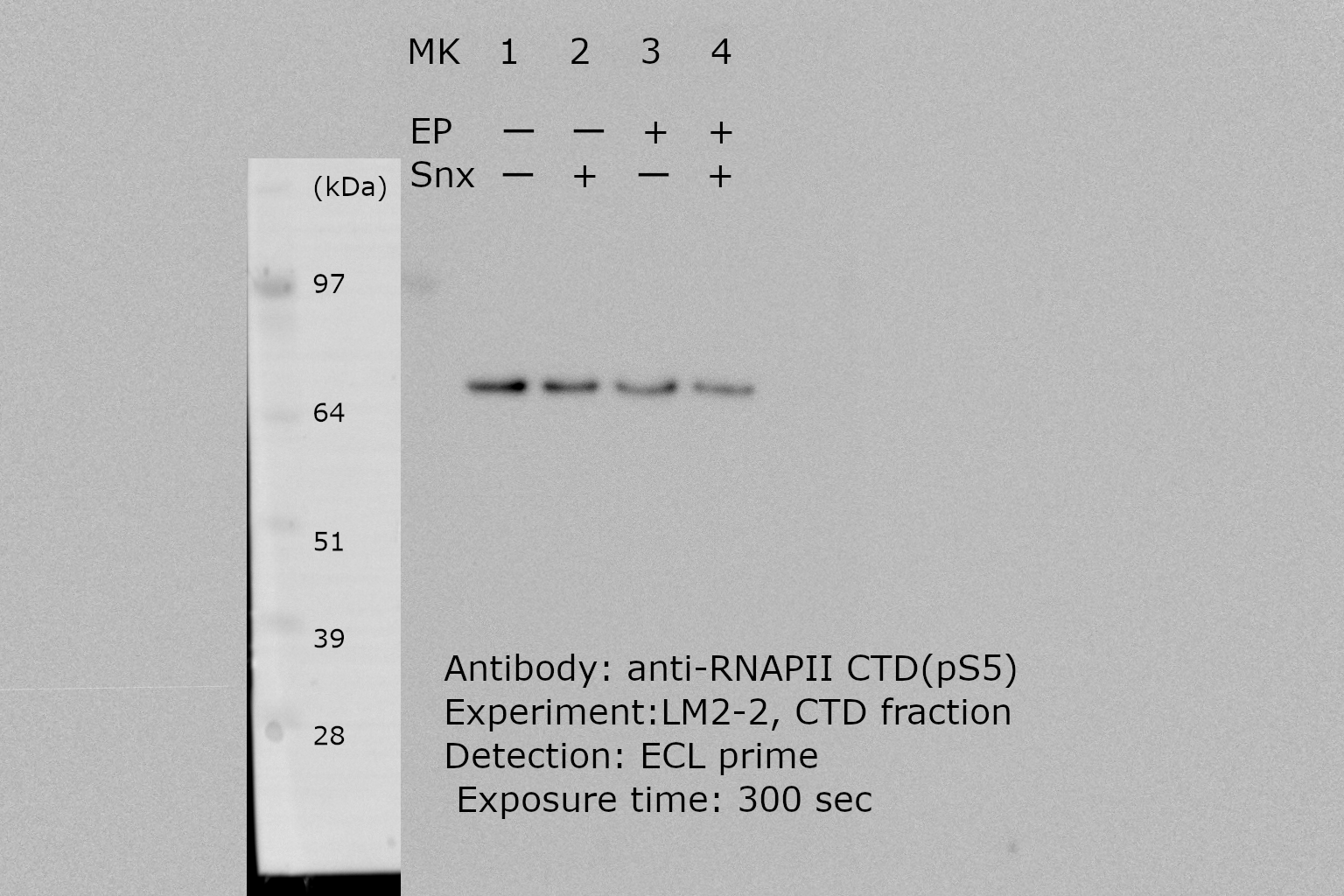

Supplement: S2 Raw Images — (ZIP) [file pone.0338485.s006.zip › LM2-2 CTD-pS5.tif]

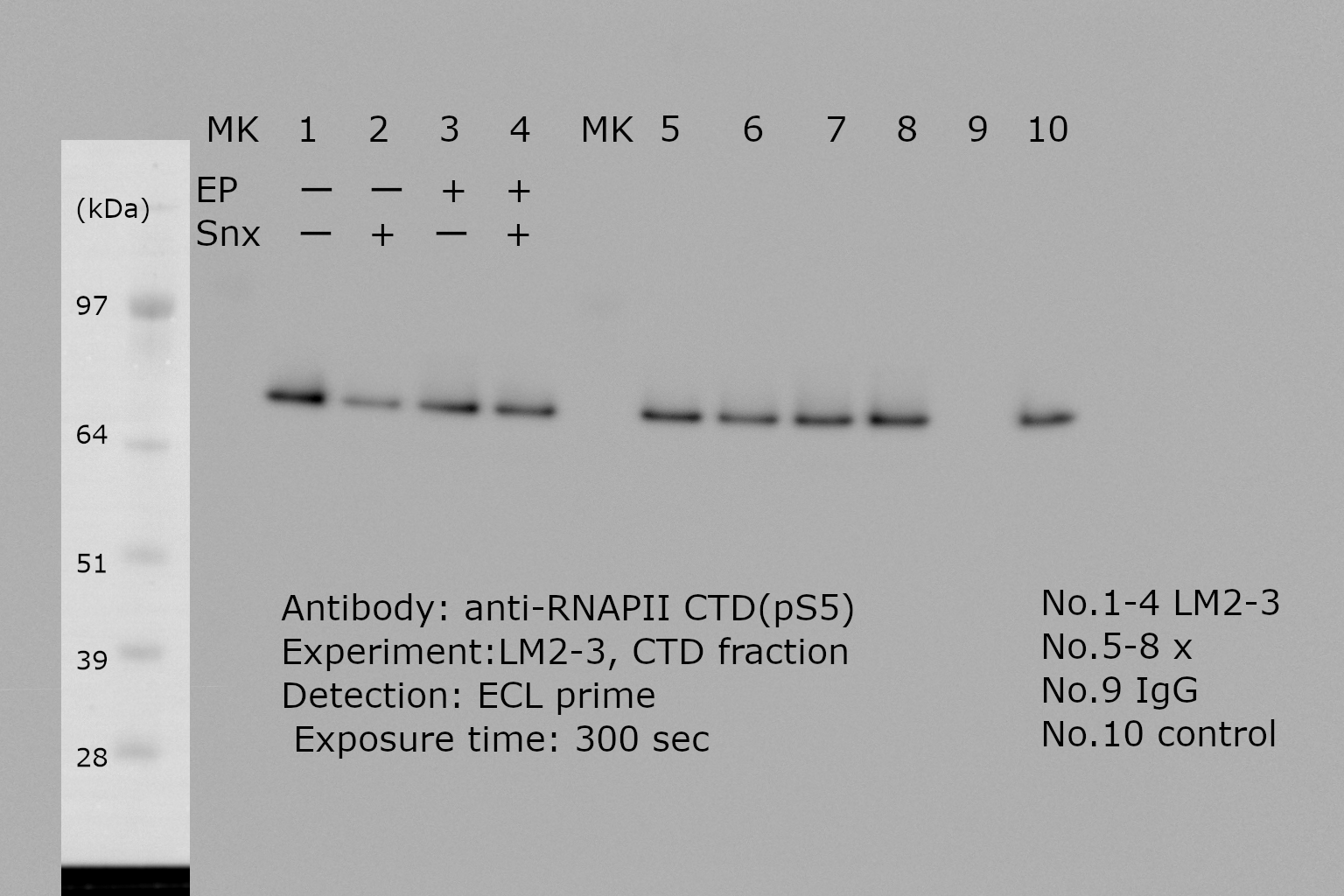

Supplement: S2 Raw Images — (ZIP) [file pone.0338485.s006.zip › LM2-3 CTD-pS5.tif]
